# Cholinesterases inhibition profiles with Ugi-derived peptidemimetics: A combined experimental and computational study

**DOI:** 10.5599/admet.3292

**Published:** 2026-06-23

**Authors:** Alma Ramić, Toni Divjak, Lucija Hadrović, Matej Pavlinić, Ana Matošević, Anita Bosak, Jakov Borovec, Bruna Bakota, Tomica Hrenar, Ines Primožič

**Affiliations:** 1 Department of Chemistry, Faculty of Science, University of Zagreb, Horvatovac 102a, Zagreb, Croatia; 2 Institute for Medical Research and Occupational Health, Ksaverska cesta 2, Zagreb, Croatia

**Keywords:** α-acylaminoacetamides, butyrylcholinesterase inhibition, predictive models of activity, Alzheimer’s disease therapeutics

## Abstract

**Background and purpose:**

Acetylcholinesterase (AChE) and butyrylcholinesterase (BChE) are crucial enzymes implicated in various neurological disorders, including Alzheimer’s disease. Developing selective inhibitors for either enzyme is one of the key therapeutic strategies. This study aimed to synthesize and evaluate a novel series of peptidomimetics for their ability to inhibit both human AChE and BChE, with a focus on identifying compounds exhibiting joint inhibitory activity or selectivity for BChE.

**Experimental approach:**

Eleven peptidomimetics were synthesized using the Ugi four-component reaction. *In vitro* enzyme inhibition assays were performed to determine the dissociation constants (*K*_i_) for both human AChE (hAChE) and human BChE (hBChE). Principal component analysis was employed to analyse the inhibition data and map compound selectivity. To investigate the molecular interactions between the peptidomimetics and the BChE active site, quantum-chemical docking simulations were conducted.

**Key results:**

All synthesized compounds exhibited reversible, micromolar inhibition of both hAChE and hBChE. Two compounds demonstrated significant BChE selectivity, with 279- and 169-fold higher preference for BChE, respectively. Principal component analysis revealed distinct clusters correlating with preferential binding to either enzyme. Docking simulations supported these findings, highlighting key stabilizing interactions (primarily π-π stacking) between the peptidomimetics and BChE, explaining superior selective and joint inhibitory activity.

**Conclusion:**

This work demonstrates the successful synthesis and characterization of novel peptidomimetics with varying degrees of hAChE and hBChE inhibition, including compounds with notable BChE selectivity. The combination of experimental data and computational modelling provides valuable insights into the structural basis of enzyme inhibition and establishes a foundation for rational design of more potent and selective cholinesterase inhibitors based on the designed scaffold.

## Introduction

**Figure 1.**
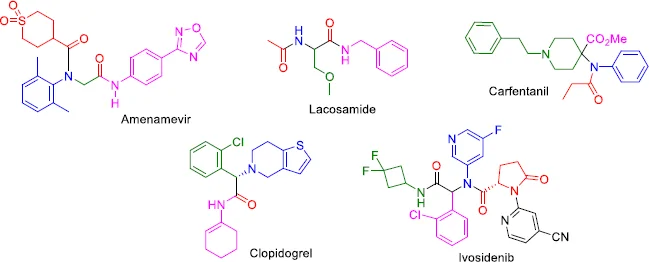
Examples of marketed drugs prepared using Ugi-4CR chemistry; the amine, aldehyde, isocyanide, and acid components are depicted in blue, green, magenta and red, respectively

Peptidomimetics are synthetic compounds designed to mimic the structure and function of peptides. In recent decades, this field has been extensively explored as a promising tool for developing new and effective therapeutic agents [1]. Multicomponent reactions, such as the Ugi four-component reaction (Ugi-4CR), offer significant advantages over traditional multistep reactions, primarily by enabling the synthesis of complex organic molecules at lower cost and faster reaction times [2]. Ugi-4CR is a single-pot synthetic method that involves the condensation of an amine, an aldehyde or a ketone, an isocyanide, and a carboxylic acid to produce a wide range of peptidomimetic compounds [3]. Due to their structural diversity and potential biological activity, these products serve as valuable intermediates in pharmaceutical research and drug discovery. Ugi-4CR was employed to synthesize various active pharmaceutical ingredients, including amenamevir [4], lacosamide [5], carfentanil [6], clopidogrel [7] and ivosidenib [8] (Figure 1).

Various methods for performing Ugi-4CR have been described in the literature, including conventional solution-phase synthesis, mechanochemical, solid-phase, ultrasound, and microwave-assisted methods [9]. Microwave synthesis can be a greener alternative to conventional synthetic methods in organic chemistry because microwave heating is more energy-efficient, directly heating the reaction mixture and leading to shorter reaction times and reduced need for organic solvents [10]. In recent years, microwave-aided synthesis has emerged as a promising synthetic method with broad applications in peptide [11] and polymer [12] synthesis and nanotechnology [13].

Acetylcholinesterase (AChE) and butyrylcholinesterase (BChE) are enzymes that play a critical role in the hydrolysis of various choline-based esters, including the neurotransmitter acetylcholine. AChE is predominantly found in neural tissues, but also in the muscles, heart, and blood, where it is bound to erythrocytes [14]. On the contrary, BChE is widely distributed throughout the body, with the highest levels in plasma and liver, and significant levels also in the brain, heart, lungs, intestines, and kidneys [15-16]. While AChE plays a crucial role in terminating synaptic transmission, the function of BChE is still being unravelled. BChE serves as a crucial backup for AChE by assisting in regulating cholinergic signalling and detoxifying certain drugs and toxins [17]. Dysregulation of AChE activity has been closely associated with neurodegenerative diseases, particularly Alzheimer’s disease. In Alzheimer’s disease, there is a decrease in acetylcholine (ACh) levels and accumulation of amyloid-beta plaques, which contribute to synaptic dysfunction and cognitive decline [18]. To counteract this, cholinesterase inhibitors such as donepezil, rivastigmine, and galantamine are used to enhance cholinergic signalling by preventing the breakdown of acetylcholine [19]. With the progression of Alzheimer’s disease, the ratio of AChE to BChE shifts in favour of BChE, so targeting BChE, as a backup to AChE, offers a promising therapeutic strategy for symptomatic treatment of the disease [20]. While AChE inhibitors have been extensively studied, both progressive (toxic carbamates and organophosphates) and reversible (*e.g.* tacrine, donepezil), BChE inhibitors have only been systematically investigated over the past few decades. For instance, BChE activity and selectivity were assessed for a series of compounds: thiazole analogues, tetrahydroacridine derivatives with a fluorobenzoic acid moiety, 4-dimethylamine flavonoid derivatives, graveolinine derivatives [21] and bambuterol [22-23]. While most compounds showed low micromolar IC_50_ values (1-10 μM), several tetrahydroacridine derivatives also exhibited nanomolar potency (below 10 nM). Furthermore, the inhibitory potential of heterocyclic scaffolds containing nitrogen, oxygen, and sulfur was explored, demonstrating that effective inhibitors can be achieved with diverse structural frameworks [24]. Thus, using the Ugi reaction, a diverse library of oxindole-lactam hybrids was recently synthesized, demonstrating promising BChE inhibitory activity in the low micromolar range [25], as well as dehydroabietylamine-derived bistetrazoles [26]. Moderate activity toward cholinesterase was demonstrated with Ugi products based on *ortho*-tolyl-isocyanide [27], and Ugi cinnamic adduct was discovered in *in silico* study to be a possible binder of butyrylcholinesterase enzyme [28].

Driven by our interest in developing highly selective BChE inhibitors [29-31] and expanding the knowledge of Ugi product-derived cholinesterase inhibitors, we employed Ugi 4-CR to synthesize peptide-like compounds via conventional and microwave-assisted methods. By varying the stereoelectronic properties of reaction components, a series of compounds with novel molecular scaffolds was prepared and evaluated as potential BChE inhibitors. Inhibition of human AChE (hAChE) and human BChE (hBChE) was evaluated, demonstrating that the molecular scaffold of the prepared compounds provides a robust platform for further optimization of selective hBChE inhibitors.

## Experimental

### Materials and methods

Reagents and solvents for compound preparation were purchased from Sigma-Aldrich (St. Louis, MO, USA) and BLD Pharmatech GmbH (Reinbek, Germany). CEM Focused Microwave TM Synthesis System (Discover SP, Matthews, NC, SAD) was used for microwave synthesis. The reactions were monitored by thin-layer chromatography plates coated with silica gel (Sigma-Aldrich, St. Louis, MO, USA). TLC plates were visualized by UV irradiation (254 nm) or by iodine fumes. 1D and 2D ^1^H and ^13^C NMR spectra were recorded on a Bruker Avance III HD 400 MHz/54 mm Ascend spectrometer (Bruker Optics Inc, Billerica, MA, USA) in deuterated chloroform or methanol at 298 K. Chemical shifts are given in ppm downfield from tetramethylsilane (TMS) as an internal standard. Hydrogen and carbon atoms of the phenyl group are marked with a Ph; of the benzyl group with a Bn; of the morpholine ring with a morph; of the *tert*-butyl group as t-bu; of the cyclohexyl ring with a chx; of *iso*-butyl as i-bu; of the formaldehyde group as form; of the acetic group as Ac; and of the tosyl group as ts. Methylene groups are marked with CH_2_ x, where x is the mark from which the component CH_2_ group is formed. Note regarding the ^1^H and ^13^C spectra of compounds **1** to **10** at 298 K, some Ugi adducts may appear as a mixture of conformers (Figures S1-S21). Melting points were determined on a Melting Point B-540 apparatus (Büchi, Essen, Germany) and are uncorrected. All compounds showed purities >97 % by HPLC analyses performed on an Agilent 1260 series instrument equipped with a quaternary pump, autosampler, column compartment, and diode-array detector (DAD). HPLC conditions: Zorbax Extend-C18 column, 4.6×250 mm, 5 μm pore size; column temperature 40 °C; flow rate 1.0 mL min^-1^; mobile phase A: 0.1 % TFA in H_2_O; mobile phase B: 0.1 % TFA in CH_3_CN; linear gradient 10/90/90/10/10 % B in time intervals 0/10/15/20/25; the volume of injection 5 μL; UV detection at 220 nm (Figures S22-S32). HRMS analyses were carried out on a Q Exactive™ Plus Hybrid Quadrupole-Orbitrap™ mass spectrometer.

### Synthesis of compounds

Conventional synthesis - general procedure: Appropriate amine (1 mmol), aldehyde (1 mmol), carbocyclic acid (1 mmol), and isocyanide (1 mmol) were weighed into a round-bottom flask, and methanol (1 mL) was added. The solution was mixed on a magnetic stirrer for 24 h. Methanol was evaporated, and the reaction mixture was made alkaline with sodium hydrogen carbonate solution (pH 8). After extraction with ethyl acetate (2×20 mL), the organic extracts were dried over anhydrous sodium sulphate. Ethyl acetate was evaporated, and the residue was purified by column chromatography (silica gel, DCM : MeOH = 9 : 1).

Microwave-aided synthesis - general procedure: Appropriate amine (1 mmol), aldehyde (1 mmol), carbocyclic acid (1 mmol), and isocyanide (1 mmol) were added to a microwave vial together with methanol (0.5 mL) and a stirrer bar. After the designated time and temperature, methanol was evaporated, and the reaction mixture was made alkaline with the addition of sodium hydrogen carbonate solution (pH 8). After extraction with ethyl acetate (2×20 mL), the organic extracts were dried over anhydrous sodium sulphate. Ethyl acetate was evaporated, and the residue was purified by column chromatography (silica gel, DCM : MeOH = 9 : 1).

*N*-benzyl-*N*-{[(morpholine-4-yl)ethylaminocarbonyl]methyl} benzamide (**1**): yellow oil, yield = 30 %; *R*_f_=0.44; ^1^H NMR (400 MHz, CD_3_OD) *δ*/ppm: 2.39-2.52 (m, 6 H, H2, H6 morph, CH_2_1 morph) 3.34-3.39 (m, 1 H, CH_2_2 morph) 3.64-3.69 (m, 4 H, H3, H5 morph) 3.82 (s, 1 H, CH_2_ form) 4.08 (s, 1 H, CH_2_ form) 4.61 (s, 1 H, CH_2_ Bn) 4.78 (s, 1 H, CH_2_ Bn) 7.20 (m, 1 H, H Bn) 7.27-7.39 (m, 4 H, H Bn) 7.41-7.55 (m, 5 H, H Ph); ^13^C NMR (100 MHz, CD_3_OD) *δ*/ppm: 37.2 (CH_2_2 morph) 50.4 (CH_2_ Bn) 50.7 (CH_2_ form) 52.1 (CH_2_ form) 54.8 (C2, C6 morph) 55.3 (CH_2_ Bn) 58.5 (CH_2_1 morph) 67.7 (C3, C5 morph) 127.8; 127.9; 128.8; 128.9; 129.42; 129.73; 129.9; 131.1; 131.2; 136.7; 137.1 (C1 Bn) 137.5; 137.8 (C1 Ph) 170.3 (C=O) 174.9 (C=O); HPLC: *t*_R_=10.56 min, 98.8 %; HRMS/^+^ESI: C_22_H_28_N_3_O_3_^+^ calculated 382.2125, found 382.2124.

*N*-benzyl-*N*-{[(morpholine-4-yl)ethylaminocarbonyl]methyl}-3-bromobenzamide (**2**): yellow solid, m.p.=109.8 °C, yield=41 %; *R*_f_=0.46; ^1^H NMR (400 MHz, CD_3_OD) *δ*/ppm: 2.40-2.49 (m, 6 H, H2, H6 morph, CH_2_1 morph) 3.37 (t, 1 H, *J*=6.6 Hz, 1 H, CH_2_2 morph) 3.64-3.69 (m, 4 H, H3, H5 morph) 3.81 (s, 1 H, CH_2_ form) 4.09 (s, 1 H, CH_2_ form) 4.58 (s, 1 H, CH_2_ Bn) 4.76 (s, 1 H, CH_2_ Bn) 7.20 (m, 1 H, H Bn) 7.30-7.41 (m, 5 H, H Ph) 7.45-7.50 (m, 1 H, H Bn) 7.62-7.71 (m, 2 H, H Bn); ^13^C (100 MHz, CD_3_OD) NMR *δ*/ppm: 37.2 (CH_2_2 morph) 50.6 (CH_2_ Bn) 52.0 (CH_2_ form) 54.6 (C2, C6 morph) 55.3 (CH_2_ Bn) 58.5 (CH_2_1 morph) 67.8 (C3, C5 morph) 123.5 (C3 Ph) 126.6; 128.3; 128.9; 129.0; 129.5; 129.9; 130.0; 130.8; 131.0; 131.6; 131.6; 134.1; 134.2; 137.3; 137.6 (C1 Bn) 138.9; 139.2 (C1 Ph) 170.1; 170.3 (C=O) 173.0 (C=O); HPLC: *t*_R_=11.69 min, 99.0 %; HRMS/^+^ESI: C_22_H_27_N_3_O_3_Br^+^ calculated 462.1210, found 462.1207.

*N*-benzyl-*N*-{[(morpholine-4-yl)ethylaminocarbonyl]methyl}-3-chlorobenzamide (**3**): yellow solid, m.p.=99.5 °C, yield=33 %; *R*_f_=0.50; ^1^H NMR (400 MHz, CD_3_OD) *δ*/ppm: 2.41-2.49 (m, 6 H, H2, H6 morph, CH_2_1 morph) 3.37 (t, *J*=6.4 Hz, 1 H, CH_2_2 morph) 3.64-3.70 (m, 4 H, H3, H5 morph) 3.82 (s, 1 H, CH_2_ form) 4.09 (s, 1 H, CH_2_ form) 4.58 (s, 1 H, CH_2_ Bn) 4.76 (s, 1 H, CH_2_ Bn) 7.20-7.22 (m, 1 H, H Bn) 7.28-7.32 (m, 1 H, H Bn) 7.34-45 (m, 5 H, H Ph) 7.46-7.56 (m, 2 H, H Bn); ^13^C NMR (100 MHz, CD_3_OD) *δ*/ppm: 37.2 (CH_2_^2^ morph) 49.1 (CH_2_ form) 50.6 (CH_2_ Bn) 52.0 (CH_2_ form) 54.6 (C2, C6 morph) 55.3 (CH_2_ Bn) 58.5 (CH_2_1 morph) 67.7 (C3, C5 morph) 126.2; 127.9; 128.1; 128.3; 128.9; 129.0; 129.5; 129.9; 131.1; 131.3; 131.4; 131.5; 135.6 (C3 Ph) 137.3; 137.6 (C1 Bn) 138.7; 139.0 (C1 Ph) 170.1; 170.3 (C=O) 173.2 (C=O); HPLC: *t*_R_=11.51 min, 99.2 %; HRMS/^+^ESI: C_22_H_27_N_3_O_3_Cl^+^ calculated 416.1735, found 416.1736.

*N*-benzyl-*N*-{[(morpholine-4-yl)ethylaminocarbonyl]methyl}-3-nitrobenzamide (**4**): yellow oil, yield=28 %; *R*_f_=0.48; ^1^H NMR (400 MHz, CD_3_OD) *δ*/ppm: 2.38-2.50 (m, 6 H, H2, H6 morph, CH_2_1 morph) 3.39 (t, *J*=6.4 Hz, 1 H, CH_2_2 morph) 3.64-3.68 (m, 4 H, H3, H5 morph) 3.84 (s, 1 H, CH_2_ form) 4.15 (s, 1 H, CH_2_ form) 4.60 (s, 1 H, CH_2_ Bn) 4.79 (s, 1 H, CH_2_ Bn) 7.20-7.22 (m, 1 H, H Bn) 7.28-7.39 (m, 4 H, H Ph) 7.70 (q, *J*=7.9 Hz, 1 H, H Bn) 7.90-7.92 (m, 1 H, H Ph) 7.32-7.41 (m, 2H, H Bn); ^13^C (100 MHz, CD_3_OD) NMR *δ*/ppm: 37.2 (CH_2_2 morph) 49.3 (CH_2_ form) 50.8 (CH_2_ Bn) 52.0 (CH_2_ form) 54.6 (C2, C6 morph) 55.3 (CH_2_ Bn) 58.5 (CH_2_1 morph) 67.7 (C3, C5 morph) 123.0; 123.2; 125.7; 128.3; 128.9; 129.0; 129.6; 129.9; 130.2; 131.3; 133.9; 134.2; 137.2; 137.5 (C1 Ph) 138.4; 138.7 (C1 Bn) 149.5 (C3 Bn) 170.0; 170.3 (C=O) 172.3 (C=O); HPLC: *t*_R_=10.78 min, 99.3 %; HRMS/^+^ESI: C_22_H_27_N_4_O_5_^+^ calculated 427.1976, found 427.1977.

*N*-benzyl-*N*-[(*tert*-butylamino)carbonylmethyl]benzamide (**5**): white solid, m.p. = 133.7-134.8 °C, yield=85 %; *R*_f_=0.85; ^1^H NMR (400 MHz, CDCl_3_) *δ*/ppm: 1.34 (s, 9 H, CH_3_ t-bu) 3.73 (s, 0.6 H, CH_2_ form) 3.97 (s, 1.4 H, CH_2_ form) 4.64 (s, 1.4 H, CH_2_ Bn) 4.81 (s, 0.6 H, CH_2_ Bn) 5.24 (s, 0.3 H, NH) 6.15 (s, 0.6 H, NH) 7.18 (s, 1 H, H4 Ph) 7.27-7.51 (m, 9 H, H2,H3,H4,H5,H6 Bn, H2,H3,H5, H6 Ph); ^13^C NMR (100 MHz, CDCl_3_) *δ*/ppm: 28.8 (CH_3_ t-bu) 35.7 (C(CH_3_)_3_ t-bu) 50.2, 51.4 (CH_2_ form) 53.0, 54.2 (CH_2_ Bn) 126.9 (C4 Bn) 127.3 (C4 Ph) 128.0 (C2, C6 Bn) 128.7 (C2, C6 Ph) 129.0 (C3, C5 Bn) 130.2 (C3, C5 Ph) 135.4 (C1 Bn) 136.2 (C1 Ph) 167.8 (C=O) 172.8 (C=O); HPLC: *t*_R_=13.14 min, 97.8 %; HRMS/^+^ESI: C_20_H_25_N_2_O_2_^+^ calculated 325.1911, found 325.1909.

*N*-isobutyl-*N*-[(cyclohexylamino)carbonylmethyl]benzamide (**6**): white solid, m.p. = 139.2-140.1 °C, yield=64 %; *R*_f_=0.83; ^1^H NMR (400 MHz, CDCl_3_) *δ*/ppm: 0.75 (bs, 4.7 H, CH_3_ i-bu) 0.99 (bs, 1.3 H, CH_3_ i-bu) 1.11-1.29 (m, 3 H, H2, H4, H6, CH_2_ chx) 1.31-1.45 (m, 2 H, H3, H5, CH_2_ chx) 1.53-1.64 (m, 1H, H4, CH_2_ chx) 1.66-1.78 (m, 2 H, H3, H5, CH_2_ chx) 1.84-1.95 (m, 2 H, H2, H6 CH_2_ chx) 2.00 (bs, 1 H, CH i-bu) 3.09-3.26 (m, 1.5 H, CH_2_ form) 3.28-3.49 (m, 0.4 H, CH_2_ form) 3.74-3.83 (m, 1 H, H1 chx) 3.85-4.00 (m, 0.4 H, CH_2_ i-bu) 4.11 (s, 1.6 H, CH_2_ i-bu) 5.67 (s, 0.2 H, NH) 6.98 (s, 0.7 H, NH) 7.31-7.48 (m, 5 H, H2, H3, H4, H5, H6 Ph); ^13^C NMR (100 MHz, CDCl_3_) *δ*/ppm: 19.9 (CH_3_ i-bu) 24.8 (C3, C5 chx) 25.6 (C4 chx) 27.1 (CH i-bu) 33.0 (C2, C6 chx) 48.1 (C1 chx) 51.9, 53.6 (CH_2_ form) 59.1 (CH_2_ i-bu) 127.1 (C4 Ph) 128.7 (C2, C6 Ph) 129.8 (C3, C5 Ph) 135.9 (C1 Ph) 168.7 (C=O) 173.5 (C=O); HPLC: *t*_R_=14.66 min, 97.8 %; HRMS/^+^ESI: C_19_H_29_N_2_O_2_^+^ calculated 317.2224 found 317.2223.

*N*-isobutyl-*N*-[(tert-butylamino)carbonylmethyl]benzamide (**7**): white solid, m.p.=114.8-116.5 °C, yield=27 %; *R*_f_=0.57; ^1^H NMR (400 MHz, CDCl_3_) *δ*/ppm: 0.76 (s, 4.6 H, CH_3_ i-bu) 0.98 (s, 1 H, CH_3_ i-bu) 1.37 (s, 9 H, CH_3_ t-bu) 2.00 (s, 1 H, CH i-bu) 3.20 (s, 1.6 H, CH_2_ form) 3.39 (s, 0.4 H, CH_2_ form) 3.84 (s, 0.3 H, CH_2_ i-bu) 4.04 (s, 1.7 H, CH_2_ i-bu) 5.41 (s, 0.1 H, NH) 6.87 (s, 0.7 H, NH) 7.32-7.48 (m, 5H, H2, H3, H4, H5, H6 Ph); ^13^C NMR (100 MHz, CDCl_3_) *δ*/ppm: 20.0 (CH_3_ i-bu) 27.2 (CH i-bu) 28.8 (CH_3_ t-bu) 52.8 (CH_2_ form) 59.1 (CH_2_ i-bu) 127.1 (C4 Ph) 128.6 (C2,C6 Ph) 129.8 (C3,C5 Ph) 135.9 (C1 Ph) 168.8 (C=O) 173.3 (C=O); HPLC: *t*_R_=13.05 min, 100.0 %; HRMS/+ESI: C_17_H_27_N_2_O_2_^+^ calculated 291.2068 found 291.2065.

*N*-isobutyl-*N*-[(tosylmethylamino)carbonylmethyl]benzamide (**8**): white solid, m.p.=80.1-80.3 °C, yield=34 %; *R*_f_=0.44; ^1^H NMR (400 MHz, CDCl_3_) *δ*/ppm: 0.56-1.04 (m, 6 H, CH_3_ i-bu) 1.84 (bs, 1 H, CH i-bu) 2.42 (s, 3 H, CH_3_ ts) 3.08 (s, 2 H, CH_2_ form) 4.09 (s, 2 H, CH_2_ i-bu) 4.68 (s, 2 H, CH_2_ ts) 7.24-7.48 (m, 7 H, H3, H5 ts, H2, H3, H4, H5, H6 Ph) 7.66-7.86 (m, 3 H, NH, H2, H6 ts); ^13^C NMR (100 MHz, CDCl_3_) *δ*/ppm: 19.9 20.7 (CH_3_ i-bu) 21.8 22.0 (CH_3_ ts) 27.0 (CH i-bu) 50.1 (CH_2_ form) 58.3 60.4 (CH_2_ i-bu) 61.2 63.9 (CH_2_ ts) 127.2 (C4 Ph) 128.7 (C2, C6 Ph) 128.9 (C2, C6 ts) 129.6 130.0 (C3, C5 Ph) 130.1 130.5 (C3, C5 ts) 134.3 (C1 Ph, C4 ts) 145.4 (C1 ts) 169.1 (C=O) 173.7 (C=O Ts); HPLC: *t*_R_=13.84 min, 99.6 %; HRMS/^+^ESI: C_21_H_27_N_2_O_4_S^+^ calculated 403.1687 found 403.1686.

*N*-benzyl-*N*-{[(morpholine-4-yl)ethylaminocarbonyl]methyl} acetamide (**9**): yellow oil, yield=26 %; *R*_f_=0.44; ^1^H NMR (400 MHz, CD_3_OD) *δ*/ppm: 2.16-2.21 (m, 3 H, CH_3_ Ac) 2.42-2.50 (m, 6 H, H2, H6 morph, CH_2_1 morph), 3.32-3.35 (m, 1 H, CH_2_2 morph), 3.66-3.69 (m, 4 H, H3, H5 morph), 3.97 (s, 2 H, CH_2_ form), 4.60 (s, 1 H, CH_2_ Bn), 4.67 (s, 1 H, CH_2_ Bn), 7.24-7.38 (m, 5 H, H Bn); ^13^C NMR (100 MHz, CD_3_OD) *δ*/ppm: 21.5; 21.6 (CH_3_ Ac) 37.1; 37.2 (CH_2_2), 49.8 (CH_2_ form), 50.7 (CH2 Bn), 51.6 (CH_2_ form), 54.4 (CH_2_ Bn), 54.6 (C2, C6 morph), 58.5 (CH_2_1), 67.7; 67.8 (C3, C5 morph) 128.0; 128.6; 128.9; 129.3; 129.7; 130.0 (Ar C) 137.7 (C1 Bn) 138.1 (C1 Bn) 170.4 (C=O) 171.0 (C=O) 174.4 (C=O); HPLC: *t*_R_=8.22 min, 98.6 %; HRMS/^+^ESI: C_21_H_27_N_2_O_4_S^+^ calculated 320.1969 found 320.1967.

*N*-benzyl-*N*-[(*tert*-butylamino)carbonylmethyl]acetamide (**10**): white solid, m.p.=115.2-115.4 °C, yield=28 %; *R*_f_=0.80; ^1^H NMR (400 MHz, CDCl_3_) *δ*/ppm: 1.16 (s, 3 H, CH_3_ t-bu) 1.32 (s, 6 H, CH_3_ t-bu) 2.12 (s, 1 H, CH_3_ Ac) 2.21 (s, 2 H, CH_3_ Ac) 3.85 (s, 0.7 H, CH_2_ form) 3.86 (s, 1.3 H, CH_2_ form) 4.61 (s, 0.7 H, CH_2_ Bn) 4.66 (s, 1.3 H, CH_2_ Bn) 5.26 (s, 0.3 H, NH) 6.06 (s, 0.6 H, NH) 7.19 (d, *J*=7.0 Hz, 1 H, H4 Bn) 7.28-7.42 (m, 4 H, H2, H3, H5, H6 Bn); ^13^C NMR (100 MHz, CDCl_3_) *δ*/ppm: 21.6 21.9 (CH_3_ Ac) 28.5 28.9 (CH_3_ t-bu) 51.2 51.3 (CH_2_ form) 53.5 53.6 (CH_2_ Bn) 126.8 128.0 (C4 Bn) 128.2 129.0 (C2, C6 Bn) 129.1 129.3 (C3, C5 Bn) 136.1 137.3 (C1 Bn) 167.2 168.2 (C=O) 171.6 171.9 (C=O); HPLC: *t*_R_=11.96 min, 96.2 %; HRMS/^+^ESI: C_15_H_23_N_2_O_4_^+^ calculated 263.1755 found 263.1754.

*N*-benzyl-*N*-[2-(*tert*-butylamino)carbonylprop-2-yl]acetamide (**11**): white solid, m.p.=94.1-95.2 °C, yield=44 %; *R*_f_=0.75; ^1^H NMR (400 MHz, CDCl_3_) *δ*/ppm: 1.34 (s, 9 H, CH_3_ t-bu) 1.41 (s, 6 H, (CH_3_)_2_C) 2.13 (s, 3 H, CH_3_ Ac) 4.64 (s, 2 H, CH_2_ Bn) 5.54 (s, 1 H, NH) 7.27-7.32 (m, 1 H, H4 Bn) 7.35-7.42 (m, 4H, H2, H3, H5, H6 Bn); ^13^C NMR (100 MHz, CDCl_3_) *δ*/ppm: 23.4 ((CH_3_)_2_C) 24.9 (CH_3_ Ac) 28.7 (CH_3_ t-bu) 48.6 51.1 (CH_2_ Bn) 63.0 ((CH_3_)_2_C) 126.2 (C4 Bn) 127.5 (C2, C6 Bn) 129.1 (C3, C5 Bn) 139.0 (C1 Bn) 171.9 (C=O), 174.1 (C=O); HPLC: *t*_R_=13.27 min, 99.6 %; HRMS/^+^ESI: C_17_H_27_N_2_O_4_^+^: calculated 291.2064 found 291.2068.

### Kinetic studies

Sources of AChE and BChE were native human erythrocytes and native human plasma, respectively, from two healthy donors at the Institute for Medical Research and Occupational Health, Croatia, following approval by the Ethics Committee of the Institute. Enzyme substrate acetylthiocholine iodide (ATCh) was purchased from Sigma-Aldrich, Steinheim, Germany, and thiol reagent 5,5′-dithiobis-2-nitrobenzoic acid (DTNB) from Sigma-Aldrich, St. Louis, USA, ATCh and DTNB were dissolved in 0.1 M sodium phosphate buffer (pH 7.4).

The AChE and BChE activity was measured by the Ellman spectrophotometric method [32] at different substrate concentrations (*s*; 0.050 to 0.50 mM) in the absence (*v*_0_) and presence (*v*_i_) of a given compound concentration (*i*) selected to inhibit the enzymes for 20 to 80 %. At least three inhibitor concentrations for each substrate concentration were used in at least two experiments. The apparent inhibition constant (*K*_i,app_) was calculated using the Hunter-Downs equation (1) and the linear regression analysis:



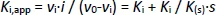

(1)


where the *y*-intercept determines the enzyme-inhibitor dissociation constants (*K*_i_), while the *x*-intercept determines the enzyme-substrate dissociation constant, *K*_(S)_. The equation was used with the assumption that, due to the low substrate concentrations used in the experiments, the substrate binds only to the catalytic site, whereas the inhibitor can bind to both sites, the catalytic and the peripheral site.

No side interactions of the tested compounds with ATCh or DTNB were detected. Measurements were done at 25 °C on a Tecan Infinite M200Pro plate reader (Austria).

### Determination of IC_50_ values

eqBChE (EC, 3.1.1.8), type IV-S lyophilized powder from horse serum (Sigma-Aldrich Chemie GmbH, Taufkirchen, Germany) was used without further purification. The activity of the enzyme was measured using Ellman’s method. [32] ATCh was used as a substrate (2.2 mM), and the reaction was monitored spectrophotometrically at 412 nm using a Molecular Devices SpectraMax iD3 Multi-Mode Microplate Reader. Assays were performed in phosphate buffer (0.1 mmol dm^-3^, pH 7.4) at 25 °C, with a total reaction volume of 0.3 mL in 96-well plates. Residual activity of the enzyme was measured in the presence of six to eight different concentrations of inhibitor [I], selected to inhibit the enzyme 10-90 %. Reagents were added in the following order: buffer, eqBChE, inhibitor (**1** to **11**), DTNB, and ATCh. Inhibitor concentrations (log [I]) were plotted against the percentage of enzyme inhibition, % (Inhibition = [1 - (rate of reaction with I) / (rate of control reaction)]×100), with 50 % inhibition identified by the linear regression method.

### Principal component analysis

Multivariate analyses of inhibition of human AChE and BChE by tested compounds were conducted using the 2^nd^-order tensor decomposition tool principal component analysis (PCA) [33-34]. Data matrix **X** (2), which has a rank *r*, is numerically decomposed into the sum of 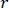 matrices **t***_i_***p***_i_***^τ^** (each one of rank 1):



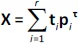

(2)


where **t***_i_* is a score vector, whereas **p***_i_* is a loading vector. PCA provides the best linear projection of multidimensional data by minimizing the least squares objective function. Score values determine the locations of original samples in the coordinate system defined by the calculated principal component axes, while loadings describe the variability in the data. Experimentally determined inhibition data were written in the data matrix **X** and PCA was performed on the data covariance matrix using our parallelized code for multi- and univariate analysis [35-38]. Eigenvector extraction was performed with the non-linear iterative partial least squares (NIPALS) algorithm [39].

### Quantum chemical docking

A comprehensive search of the configurational space of small molecules within the BChE active site was performed using quantum-chemical docking. A parallelized Monte Carlo sampling algorithm was used for the structure generation of configurations of molecules docked into the BChE’s active site (10^7^ structures) [40]. A semi-flexible quantum-mechanical approach to molecular docking was employed, accounting for all translational, rotational, and torsional degrees of freedom of the selected α-acylaminoacetamides, and configurations with atomic overlaps were discarded. Initially, the binding energies within the active site were estimated using single‑point calculations with the PM7 Hamiltonian [41]. The top 1000 local minima, sorted by calculated enthalpies, were further refined by geometry optimization, clustered, and ranked by estimated binding enthalpies. The lowest-energy structures obtained were further analysed and compared with experimental crystal structures.

Single-point quantum-chemical calculations using the PM7 method were performed to estimate binding energies at the active site. For the top 1000 local minima, additional full geometry optimizations were performed. These refined structures with optimized geometries were clustered by similarity and ranked based on the estimated binding energy. The results were analysed using automated molecular interactions search subroutine and further visually inspected. The lowest-energy structures obtained were further analysed and compared with experimental crystal structures of hBChE-tacrine complex (PDB 4BDS) [42].

## Results and discussion

### Synthesis of compounds

**Figure 2.**
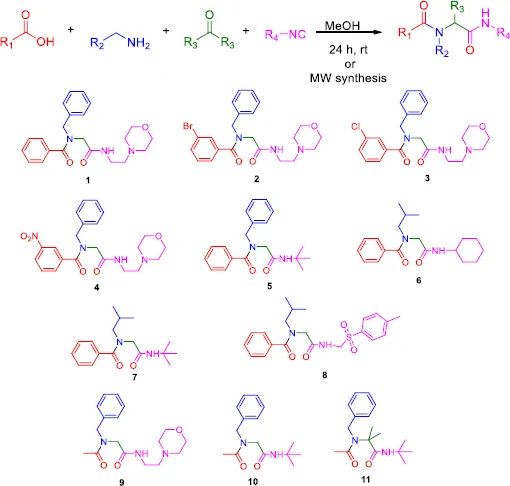
Synthesis of peptidomimetics **1** - **11** and their structure (the amine, aldehyde, isocyanide, and acid components are depicted in blue, green, magenta, and red, respectively)

Ugi-4CR was used to prepare a series of 11 novel peptide-like compounds, which all have an α-acylaminoacetamide skeleton but differ in the acyl part (R1: phenyl and methyl groups), substituents at the acylamino nitrogen atom (R2: benzyl and *iso*-butyl groups), substituent at α-acetamide carbon atom (R3: methyl group, compound **11**), and α-acetamide nitrogen atom (R4: morpholinylethyl, *tert*-butyl, cyclohexyl, and *p*-toluensulfonylmethyl groups). All compounds were synthesised by both conventional and microwave-assisted synthesis (Figure 2).

In the initial approach, a conventional Ugi-4CR was employed to synthesize the compounds under standard conditions reported in the literature, in which the formaldehyde, amine, carboxylic acid, and isocyanide components were mixed in methanol at room temperature for 24 h [43]. However, as conventional Ugi-4CR for some of the desired compounds yielded complex mixtures with low yields, we turned to microwave-assisted synthesis. First, reaction conditions for the microwave synthesis of compound **5** were optimized by varying solvent, temperature, and reaction time (Table 1).

**Table 1.** Reaction condition screening for microwave synthesis of compound **5.**

Entry	Solvent	Temperature, °C	Reaction time, min	Yield, %
1	MeOH	120	10	71
2	EtOH	120	10	61
3	*i*-PrOH	120	10	40
4	MeOH	130	10	58
5	MeOH	140	10	39
6	MeOH	120	5	49
7	MeOH	120	15	60
8	MeOH	110	15	83

Initially, solvent screening revealed that methanol was optimal for the reaction (120 °C, 10 minutes, entries 1 to 3). Increasing the reaction temperature to 130 or 140 °C resulted in yields of 58 and 39 %, respectively, compared to 120 °C (entries 4 and 5). A shorter reaction time of 5 minutes at 120 °C also lowered the yield to 49 % (entry 6), as well as a longer reaction time of 15 minutes (entry 7). Further optimization with methanol as the solvent and a reaction time of 15 minutes established 110 °C as the optimal temperature, yielding an 83 % yield (entry 8).

Compounds **1** to **5** were synthesized using the established reaction conditions. Compound **9** was prepared analogously, substituting acetic acid for benzoic acid. To minimize side-product formation during the synthesis of compounds **6** to **8**, the reaction temperature was increased to 130 °C and the reaction time shortened to 4 minutes. Compound **10**, synthesized from acetic acid, benzylamine, formaldehyde, and *tert*-butyl isocyanide, required a reaction temperature of 130 °C and a reaction time of 10 minutes.

For the synthesis of compound **11**, utilizing acetone as the carbonyl component, several reaction conditions were investigated. As ketones typically require pre-condensation with amines to form an imine before the addition of carboxylic acid and isocyanide in Ugi-4CR reactions [44], imine formation from acetone and benzylamine via thin-layer chromatography within 5 minutes was confirmed. Microwave irradiation at 140 °C for 15 minutes was initially investigated using both methanol and acetonitrile as solvents; methanol proved superior. Lowering the temperature to 130 or 120 °C, with a corresponding increase in reaction time to 25 minutes, resulted in decreased yields of 29 and 10 %, respectively.

The structures of all prepared compounds were deduced and confirmed by 1D and 2D ^1^H and ^13^C NMR experiments and HRMS. Because these compounds contain amide bonds around which slow rotation is possible, they can exist as a mixture of *cis*- and *trans*-conformers [45].

### Inhibition of cholinesterases

All synthesized compounds were evaluated for their ability to inhibit both human acetylcholinesterase (hAChE) and human butyrylcholinesterase (hBChE). Reversible inhibition was observed for all compounds against both enzymes. Initial screening for anticholinesterase activity was performed using commercially available horse butyrylcholinesterase (eqBChE) to determine IC_50_ values, summarized in Table 2.

**Table 2.** Determined reversible inhibition of eqBChE (IC_50_ values), hAChE and hBChE (enzyme-inhibitor dissociation constants *K*_i_ and enzyme-substrate dissociation constant *K_S_*) for all prepared compounds **1** to **11**. All data were obtained from at least three experiments. The selectivity of the corresponding compound is determined as the ratio of *K*_i_ constants for hAChE and hBChE

Compound	eqBChE	hAChE		hBChE		*K*_i(AChE)_/ *K*_i(BChE)_
	IC_50_, μM	*K*_i_ / μM	*K*_S_ / mM^a^	*K*_i_ / μM	*K_S_* / mM^a^	
**1**	10.0 ± 0.6	151 ± 8	0.31 ± 0.03 (m)	5.6 ± 0.3	0.59 ± 0.07 (m)	25.2
**2**	95.7 ± 1.7	210 ± 13	0.34 ± 0.03 (m)	59 ± 3	0.46 ± 0.05 (m)	3.6
**3**	39.3 ± 4.3	185 ± 13	0.46 ± 0.06 (m)	21 ± 1	1.1 ± 0.13 (m)	8.8
**4**	63.0 ± 2.6	196 ± 15	0.27 ± 0.03 (c)	49 ± 2	1.2 ± 0.21 (m)	4.0
**5**	115.9 ± 8.5	559 ± 21	0.94 ± 0.13 (m)	1.9 ± 0.1	0.65 ± 0.06 (c)	279
**6**	425.4 ± 27.8	434 ± 20	0.41 ± 0.03 (c)	70 ± 4	0.33 ± 0.03 (m)	6.2
**7**	224.0 ± 3.3	334 ± 18	0.29 ± 0.02 (c)	55 ± 2	0.46 ± 0.04 (c)	6.0
**8**	45.0 ± 1.8	338 ± 12	0.38 ± 0.02 (c)	2.1 ± 0.1	0.87 ± 0.13 (m)	169
**9**	269.8 ± 0.1	206 ± 12	0.36 ± 0.03 (m)	47 ± 2	0.44 ± 0.04 (c)	4.4
**10**	104.7 ± 0.7	199 ± 8	0.43 ± 0.04 (m)	20 ± 1	1.3 ± 0.26 (c)	9.9
**11**	445.7 ± 9.3	138 ± 10	0.26 ± 0.03 (m)	51 ±2	0.56 ± 0.05 (c)	2.7
Galantamine [47]		0.52 ± 003		1.08 ± 0.08		0.5
Donepezil [47]		0.024 ± 0.007		2.33 ± 0.73		0.01

^a^type of inhibition: mixed (m) and competitive (c)

Despite differences in potency and selectivity, the fundamental inhibitor mechanisms for eqBChE, hBChE, and hAChE are generally conserved. Commercially available equine BChE was used as an enzyme of choice for initial inhibitor screening due to its high homology in primary structure with human BChE [46]. The primary structure of equine and human BChE differ in 15 amino acids, and comparison of the crystal structure of human BChE and modelled structure of equine BChE suggests that only Thr69, located close to the peripheral site of equine BChE and 150 nm far from the acetylation site, could affect the different inhibitory activity determined for carbamates [46]. Therefore, eqBChE was used as a useful, preliminary model to efficiently flag compounds worthy of further investigation against both human cholinesterases.

The compounds demonstrated varying potency against equine (eqBChE), with inhibition ranging from 10.0 μM (compound **1**) to 445.7 μM (compound **11**), with compound **1** being the most potent, followed by compounds **8** (45.0 μM) and **3** (39.3 μM).

Following confirmation that all compounds inhibited eqBChE in the micromolar range (Table 2), their ability to inhibit human BChE and human AChE was assessed by determining enzyme-inhibitor dissociation constants, *K*_i_ (Table 2).

All compounds reversibly inhibited both hBChE and hAChE, with *K*_i_ values ranging from 1.9 to 70 μM for hBChE and 138 to 559 μM for hAChE. Compounds **5** and **8** were the most potent hBChE inhibitors and compounds **1** and **11** (*K*_i_ = 138 μM) hAChE inhibitors. Benzoic acid, as an acid component in the Ugi reaction, generally provided higher inhibition potency toward hBChE than acetic acid derivatives **9** to **11**. Notably, benzoic acid derivative **1** exhibited four to eight times higher affinity for hBChE than compounds **2**, **3**, and **4**, which contained *meta*-substituted bromo, chloro, or nitro groups. Electron-withdrawing groups on the benzene ring (Br, Cl, NO_2_) of the benzoic acid generally increased the BChE *K*_i_ values, indicating that unfavourable electrostatic interactions were introduced besides the expected steric factors.

At the same time, acetic acid derivative **9** showed comparable hBChE inhibition to compounds **2** and **4** with *meta-*substituted bromo and nitro benzoic acid. As expected, acetic derivative **11** proved to be the best inhibitor for hAChE (isostructural to ACh). The switch from formaldehyde to acetone (compound **10** to compound **11**) increased the *K*_i_ values by 2.6-fold for hBChE, but it is reversed for hAChE. This suggests that the acetone's methyl groups introduced steric bulk that is unfavourable in hBChE but had favourable hydrophobic interactions within the hAChE active site. Isocyanide and amine components in compounds that were the best inhibitors of both enzymes were different and had a lesser amount of influence on inhibition potency.

Overall, the compounds exhibited higher affinity for hBChE, with selectivity ratios (*K*_i_(AChE)/*K*_i_(BChE)) ranging from 2.7 to 279. Benzoic acid derivative **5** displayed the strongest selectivity for hBChE (ratio of 279), followed by compound **8** (ratio of 169), suggesting that these two compounds are promising candidates for further development of hBChE-selective inhibitors.

Generally, compounds from the series bind to both enzymes, occupy the catalytic centre of both enzymes and compete for the binding with substrate ATCh, and more often form additional interactions with amino acids of the peripheric aromatic site, demonstrating the mixed type of inhibition. Inhibition potency of compounds **5** and **8** towards BChE corresponded to that of currently approved Alzheimer’s disease drugs galantamine and donepezil, pointing to their potential use as BChE selective anti-AD drug.

### Principal component analysis

**Figure 3.**
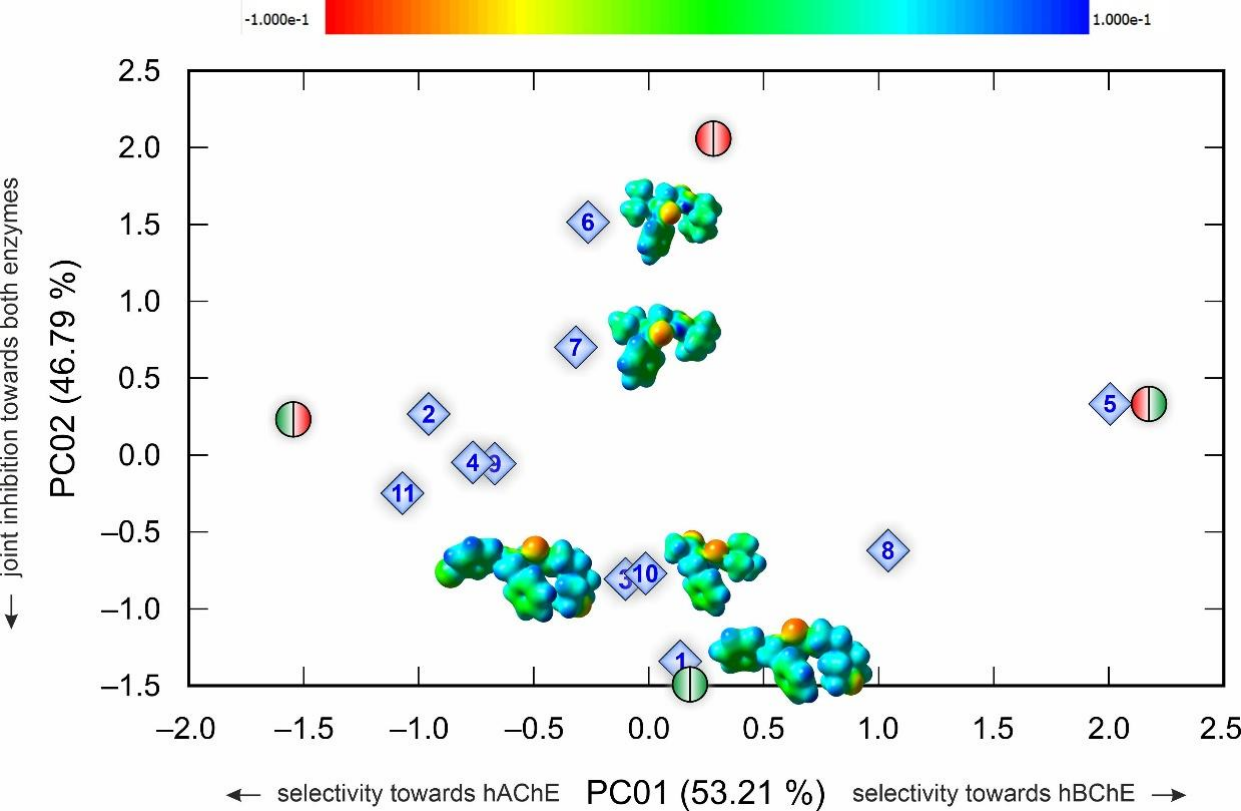
Factor scores of the reversible inhibition data for compounds **1** to **11** spanned by the first two principal components. (Red/red circle: bad for hAChE and bad for hBChE, green/green circle: good for hAChE and good for hBChE, green/red circle: good for hAChE and bad for hBChE, red/green circle: bad for hAChE and good for hBChE). Molecular charge distribution for compounds **1**, **3**, **10**, **7** and **6** visualized by electrostatic potential mapped on the total electronic density calculated at B3LYP-D3BJ/6-31G(d) level of the theory (isovalue for density: 0.01)

To better understand the selectivity and joint activities of the investigated compounds, we performed data-augmented PCA of the inhibition data. PCA can reveal distinct selectivity patterns among chemical compounds by mapping their interactions with two enzymes into a lower-dimensional space, thereby separating compounds that preferentially bind one enzyme from those that bind the other. Joint activities where a compound affects both enzymes will cluster together in the PCA space, indicating a possible similar mechanism or binding profile. By visualizing compound distributions in a rotated, reduced space spanned by augmented data, PCA highlights which compounds are selective for a single enzyme and which exhibit activity towards both enzymes. Augmenting the data with the obtained min/max values for each enzyme, we created a more robust representation with clear physical meaning, where the first principal component (PC1) aligns with a predefined "maximum" direction. This is an advantage for understanding the data because the reduced space spanned was visualized based on individual enzymatic activities. Classification based on analysis performed for hAChE and hBChE data from Table 2 is presented in Figure 3. Selectivity/joint inhibition reduced space was spanned by 4 distinct points that define the *selectivity directions* and the *joint inhibition direction*. The direction from the green/red circle (good for hAChE and bad for hBChE) on the left to the red/green circle (bad for hAChE and good for hBChE) on the right along the 1^st^ principal component represents a path in the rotated reduced space where selectivity changes from hAChE to the selectivity toward hBChE. Direction from red/red circle (bad for hAChE and bad for hBChE) on the top to green/green circle (good for hAChE and good for hBChE) on the bottom in a negative direction of the 2^nd^ principal component represents a path in the reduced space from overall bad joint activities to good joint activities. The augmented points are not perfectly aligned perpendicularly (or horizontally) because the principal components are influenced by all points, and their directions represent statistical weights and variance across them. These data can be used in the future to establish inhibition/property regression models.

Among the investigated compounds, compound **5** shows the best selective inhibition of hBChE, and, based on the value of the 1^st^ principal component score, compound **11** shows the worst selective inhibition of hBChE (but the best selective inhibition of hAChE). Score values from the 1^st^ principal component are directly connected to the selectivity of investigated compounds, which is shown by green/red and red/green circles. In the analysis of joint inhibition activities, based on the 2^nd^ principal component scores, compound **1** is the best joint inhibitor and compound **6** is the worst joint inhibitor towards both enzymes. Score values from the 2^nd^ principal component are directly connected to the joint inhibition. To connect the molecular structure with the experimental data, the molecular charge distributions for compounds **1**, **3**, **10**, **7** and **6** are presented in Figure 3. The compounds are listed in order of their joint inhibitory potency. Although this activity is governed by a combination of steric and electronic effects, Figure 1 reveals that regions of neutral charge distribution (depicted in green) are detrimental to inhibition (compounds **6** and **7**), while positively charged groups contribute favorably (compounds **1**, **3** and **10**). These findings prompted further investigation via quantum-chemical docking to characterize the specific interactions occurring within the active sites.

### Quantum chemical docking

Both hAChE and hBChE are serine hydrolases sharing a similar globular form, possessing a conserved catalytic triad (Ser-His-Glu) vital for choline ester hydrolysis [48]. This triad resides within a deep active site gorge, influencing substrate access and binding. The active site comprises key domains: a) an esteratic site with the catalytic serine triad (Ser, His, Glu), b) an acyl pocket - a hydrophobic region hosting the ester’s acyl group (**hAChE** (pdb 4ey4): Phe295, Phe297, Phe338, Trp236; **hBChE** (pdb 1p0i): Leu286, Phe329, Phe398, Trp231), c) a choline subsite recognizing the substrate’s quaternary ammonium group (**hAChE**: Trp86, Tyr337; **hBChE**: Trp82, Ala328), and d) an oxyanion hole formed by N-H dipoles stabilizing the substrate’s carbonyl oxygen atom (**hAChE**: Gly121, Gly122, Ala204; **hBChE**: Gly116, Gly117, Ala119). Generally, hAChE possesses a narrower, deeper, and more defined catalytic gorge optimized for ACh binding. It has a prominent peripheric aromatic site (PAS, Trp286, Tyr124, Tyr72, Tyr341, Asp74) critical for ACh binding. hBChE has a wider, more open catalytic gorge that accepts a broader range of substrates, including butyrylcholine, succinylcholine and cocaine, with a less defined PAS (Tyr332, Asp70). Overall, the differing ligand binding specificities of AChE and BChE can be largely explained by a significant reduction in aromatic residues within the hBChE catalytic gorge. While the hAChE gorge contains 10 such residues, hBChE has only four conserved [49].

Using quantum-chemical docking and the PM7 Hamiltonian, compounds **1**-**11** were successfully docked into the active sites of hAChE and hBChE. The resulting structures were observed to be located deep within the active site gorges (Figures 4-7 and Figures S45-S55).

**Figure 4.**
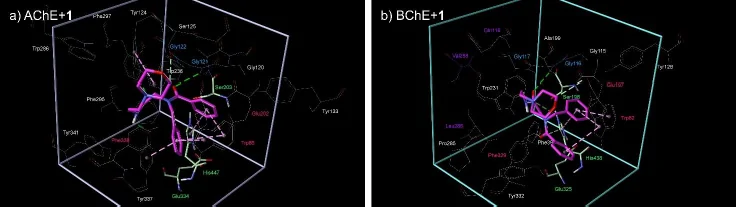
Placement of compound **1** inside the active site of **a)** AChE and **b)** BChE obtained by extensive quantum-chemical docking and geometry optimization at the PM7 semiempirical level of the theory. (Violet color for carbon atoms: one of the docked structures of minimal energy, non-polar hydrogen atoms are omitted for clarity)

**Figure 5.**
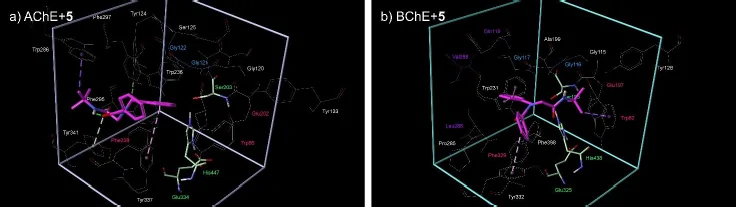
Placement of compound **5** inside the active site of **a)** AChE and **b)** BChE obtained by extensive quantum-chemical docking and geometry optimization at the PM7 semiempirical level of the theory. (Violet color for carbon atoms: one of the docked structures of minimal energy, non-polar hydrogen atoms are omitted for clarity)

**Figure 6.**
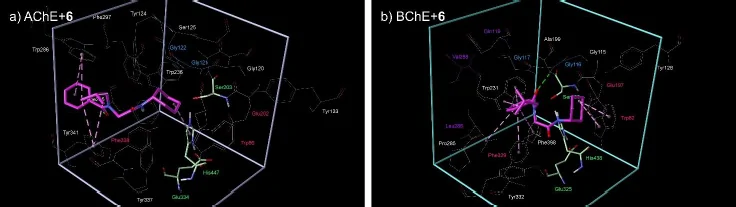
Placement of compound **6** inside the active site of **a)** AChE and **b)** BChE obtained by extensive quantum-chemical docking and geometry optimization at the PM7 semiempirical level of the theory. (Violet color for carbon atoms: one of the docked structures of minimal energy, non-polar hydrogen atoms are omitted for clarity)

**Figure 7.**
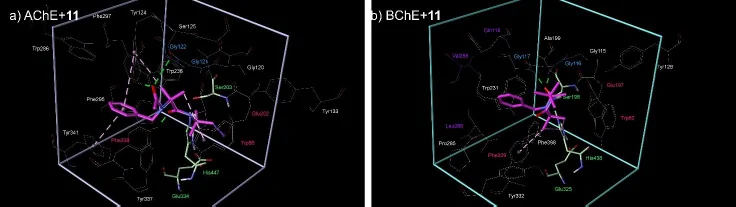
Placement of compound **11** inside the active site of **a)** AChE and **b)** BChE obtained by extensive quantum-chemical docking and geometry optimization at the PM7 semiempirical level of the theory. (Violet color for carbon atoms: one of the docked structures of minimal energy, non-polar hydrogen atoms are omitted for clarity)

The most important interactions between all α-acylaminoacetamides and hAChE/hBChE are hydrophobic, π-π interactions and/or Tshape complexes/hydrogen bonds with various amino acids. Within the active site of hAChE, **1** interacts *via* π-π interactions with Trp86 and Try337 and hydrogen bonds with Tyr124 and Gly121/122 (Figure 4a, Table 3). It is similarly placed in hBChE where it interacts with Trp82 and Gly116/117 (Figure 4b, Table 4). Compound **5** was interacting with hAChE *via* a T-shape complex with Tyr337 and a strong hydrogen bond with Phe295, CH_3_-π interactions with Trp286, and additional electrostatic interactions with Tyr124/341 (Figure 5a, Table 3). Within the larger active site of hBChE, **5** is better placed and interacts with Trp82 (CH_3_-π), T-shape complex with Tyr332 and Trp231 (Figure 5b, Table 4). Within the hAChE active site, compound **6** creates a strong hydrogen bond with Tyr124 and forms a T-shaped complex with Trp286 and CH_2_-π interactions with Tyr341 (Figure 6a, Table 3). In the hBChE **6** interacts electrostatically with Trp82, forms a hydrogen bond with Gly116/117, a T-shape complex with Trp231 and Phe329, and interacts additionally with Leu286 (Figure 6b, Table 4). Compound **11** is interacting with hAChE *via* CH_3_-π interactions with Trp86, a hydrogen bond with Tyr124 and Tyr337, and π-π interactions with Tyr341 (Figure 7a, Table 3). Within the hBChE active site, interactions are a three-centred hydrogen bond with Gly116/117 and CH_3_-π interactions with Phe329 (Figure 7b, Table 4).

**Table 3.** The most important molecular interactions between α-acylaminoacetamides and selected amino acids in the active site of hAChE (interatomic distances for complexes optimized at the PM7 level of theory are given as the smallest ring centroid distances (π-π) or heteroatom distances (H-bond) between docked molecules and amino acids in the active site)

Comp.	*r* / nm					
	Trp86	Tyr124	Trp236	Phe338	Tyr337	Tyr341
**1**	0.4138 (π-π)	-	-	-	0.2471, 0.3670 (H-bond, π-π)	-
**2**	0.3757 (electrostatic)	0.2663 (H-bond)	-	-	0.4583 (electrostatic)	0.3828 (π-π)
**3**	-	-	-	-	-	0.4924 (T-shape)
**4**	0.4763 (electrostatic)	0.3150 (H-bond)	-	0.5041 (T-shape)	0.2616 (H-bond)	0.5114 (T-shape)
**5**	-	0.2396 (H-bond)	-	-	0.2797 (H-bond)	0.2966 (electrostatic)
**6**	-	0.2582 (H-bond)	-	-	-	0.4080 (CH_3_-π)
**7**	-	0.2657 (H-bond)	-	-	-	0.4350 (CH_3_-π)
**8**	-	-	-	-	0.2659 (H-bond)	0.3765 (T-shape)
**9**	-	0.2765 (H-bond)	-	-	-	0.4526 (T-shape)
**10**	0.3376 (CH_2_-π)	-	-	-	0.2644 (H-bond)	-
**11**	0.3334 (CH_3_-π)	0.2862 (H-bond)	-	-	0.2782 (H-bond)	0.4392 (π-π)

**Table 4.** The most important molecular interactions between α-acylaminoacetamides and selected amino acids in the active site of hBChE (interatomic distances for complexes optimized at the PM7 level of theory are given as the smallest ring centroid distances (π-π) or heteroatom distances (H-bond) between docked molecules and amino acids in the active site)

Comp.	*r* / nm					
	Trp82	Trp231	Leu286	Phe329	Tyr332	Phe398
**1**	0.4075 (π-π)	-	-	0.2546 (CO-π)	-	-
**2**	-	-	-	0.5447 (T-shape)	0.4511 (π-π)	-
**3**	0.3754 (π-π)	0.5350 (electrostatic)	0.5003 (electrostatic)	-	-	-
**4**	0.5259 (π-π)	0.4528 (T-shape)	-	0.5429 (T-shape)	-	-
**5**	0.3508 (CH_3_-π)	0.5023 (T-shape)	-	-	0.4724 (T-shape)	-
**6**	0.4221 (electrostatic)	0.4537 (T-shape)	0.5322 (electrostatic)	0.5293 (T-shape)	-	0.5301 (T-shape)
**7**	0.4541 (T-shape)	0.3148 (CH_3_-π)	-	0.5183 (CH_3_-π)	-	-
**8**	0.4265 (T-shape)	0.5062 (T-shape)	0.5211 (electrostatic)	0.5041 (T-shape)	-	-
**9**	-	0.4853 (T-shape)	0.5166 (electrostatic)	0.5388 (T-shape)	-	-
**10**	0.3791 (CH_2_-π)	-	-	-	-	-
**11**	-	-	-	0.4215 (CH_2_-π)	-	-

Analysis of calculated standard Gibbs binding energies for optimized complexes at the PM7 semiempirical level of theory for all compounds (Figures 4 to 7 and Figures S45 to S55, Table 5) partially supported the experimental data. In the case of joint inhibition, where compound **1** is the best overall joint inhibitor and compound **6** is the worst, for hAChE, the standard Gibbs energy of binding for **1** is the 2^nd^-lowest, whereas for **6** it is the highest. For hBChE, the data do not fully support the experimental findings; better insight can be gained from interaction analysis within the active site.

**Table 5.** Calculated standard enthalpies, entropy contributions (at room temperature), and Gibbs free energies of binding for optimized inhibitor complexes within the active sites of hAChE and hBChE at the PM7 semiempirical level of theory

Comp.	hAChE			hBChE		
	Δ_b_*H*^○^ / kJ mol^-1^	*T*_298.15 K_·Δ_b_*S*^○^ / kJ mol^-1^	Δ_b_*G*^○^ / kJ mol^-1^	Δ_b_*H*^○^ / kJ mol^-1^	*T*_298.15 K_·Δ_b_*S*^○^ / kJ mol^-1^	Δ_b_*G*^○^ / kJ mol^-1^
**1**	−368.84	−96.53	−272.31	−282.25	−80.05	−202.19
**2**	−340.96	−94.92	−246.04	−330.96	−80.90	−250.05
**3**	−353.17	−98.94	−254.24	−407.73	−90.06	−317.67
**4**	−350.32	−98.32	−252.00	−401.27	−89.89	−311.38
**5**	−319.62	−84.28	−235.35	−279.52	−66.77	−212.75
**6**	−272.60	−80.54	−192.06	−311.28	−62.39	−248.89
**7**	−293.48	−86.28	−207.21	−349.59	−69.21	−280.38
**8**	−336.22	−92.29	−243.93	−365.83	−95.28	−270.55
**9**	−357.16	−80.37	−276.79	−332.72	−66.92	−265.80
**10**	−279.57	−71.49	−208.08	−380.15	−75.21	−304.94
**11**	−272.61	−77.71	−194.90	−296.90	−72.42	−224.49

### Physico-chemical properties, lipophilic and ligand efficiency

**Figure 8.**
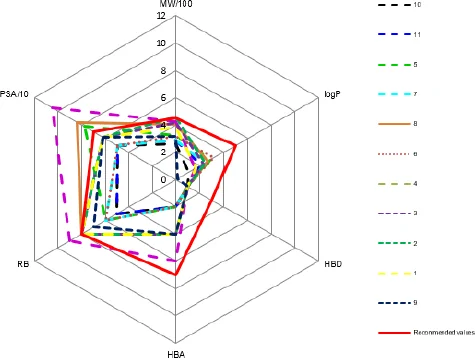
Radar plot of physico-chemical properties of prepared compounds. MW stands for molecular weight, log*P* for hydrophobicity, HBD for number of hydrogen bond donors, HBA for hydrogen bond acceptors, RB for the number of rotatable bonds and PSA for polar surface area. The recommended values for the CNS-active drugs are presented by a red line

The potential of compounds to cross the blood-brain barrier (BBB) was assessed *in silico* by comparing their calculated physico-chemical properties (molecular weight (MW), hydrophobicity (log *P*), the number of hydrogen bond donors (HBDs) and acceptors (HBAs), rotatable bonds (RBs), and polar surface area (PSA)) against recommended thresholds for passive transport into the central nervous system (CNS) by known CNS-active drugs [50-52]. Based on the calculated values (Figure 8, Table S1), most of the tested compounds are predicted to penetrate the BBB following oral administration. Compounds **3**, **5** and **8** had a higher number of PSA than recommended and therefore should have a moderate possibility for passing the BBB. Also, compound **3** had a slightly higher number of RB than recommended.

Analysis of the calculated log *P* parameter allows for correlation with initial activity and calculation of Ligand Efficiency (LE) and Lipophilic Efficiency (LLE). These metrics are crucial for identifying promising initial hits and guiding the optimization of compounds into viable leads, as detailed in Table S1. Generally, higher LE and LLE values are preferred, indicating a favourable balance between potency and lipophilicity. However, this relationship isn't always straightforward; very high lipophilicity (high log*P*) can negatively impact solubility and bioavailability, even with good LE/LLE values. Consequently, compound **1** represents the strongest lead in this series, exhibiting a relatively high p*K*_i_ for hBChE (5.25) and a moderate log *P* (1.7), resulting in a good LLE value for hBChE (3.56). In contrast, compounds **5** and **8** displayed the high p*K*_i_ values for hBChE, but their significantly higher log*P* values (2.94 and 2.68, respectively) reduce their LLE values compared to compound **1**. This suggests that reducing lipophilicity could be a beneficial optimization strategy for these compounds. With a p*K*_i_ of 3.86, a log*P* of 2.09, and a resulting LLE of 2.20 for hAChE, compound **11** represents a candidate that could be further optimized for potency.

## Conclusions

In summary, this study successfully synthesized and characterized a novel series of peptidomimetics demonstrating reversible, micromolar inhibition of both human acetylcholinesterase (hAChE) and butyrylcholinesterase (hBChE). The biological evaluation of the synthesized compounds revealed a distinct preference for hBChE over hAChE. This indicates that the studied scaffold possesses an inherent selectivity for the BChE active site, which may be attributed to the larger active site of BChE compared to the more constricted gorge of AChE. Notably, compounds **5** and **8** exhibited significant selectivity for hBChE, displaying 279- and 169-fold higher preference, respectively. However, the lipophilicity of those compounds is high and the introduction of polar functional groups or substituents to decrease log *P* while maintaining or improving potency is necessary. Therefore, compound **1** can be considered the primary lead for both enzymes in this series, as it has a good balance of potency and lipophilicity.

Principal component analysis effectively differentiated compounds based on their enzyme selectivity or joint inhibition, and these findings were supported by quantum-chemical docking simulations, which revealed key driving interactions. This integrated approach, combining experimental enzyme inhibition assays with computational modelling, not only elucidates the structural basis of peptidomimetic scaffold binding to cholinesterases but also establishes a strong foundation for the rational design of more potent and selective cholinesterase inhibitors with potential therapeutic applications in neurological disorders such as Alzheimer’s disease.

## Supplementary material

Additional data are available at https://pub.iapchem.org/ojs/index.php/admet/article/view/3292, or from the corresponding author on request.


