# Supplementary material for: Cholinesterases inhibition profiles with Ugi-derived peptidemimetics: A combined experimental and computational study
Source: ADMET DMPK. 2026 Jun 23;14:3292. doi: 10.5599/admet.3292 (PMC13499666; doi:10.5599/admet.3292)
Supplement: Supplementary file 1 [file ADMET-14-3292-S1.pdf]

ADMET & DMPK **14** (2026) S3292

Open Access : ISSN : 1848-7718
http://www.pub.iapchem.org/ojs/index.php/admet

Supplementary material to

Cholinesterases inhibition profiles with Ugi-derived peptide-mimetics: A combined experimental and computational study

Alma Ramić[1], Toni Divjak[1], Lucija Hadrović[1], Matej Pavlinić[1], Ana Matošević[2], Anita Bosak[2], Jakov Borovec[1], Bruna Bakota[1], Tomica Hrenar[1] and Ines Primožič1[1]

[1]*Department of Chemistry, Faculty of Science, University of Zagreb, Horvatovac 102a, Zagreb, Croatia*
[2]*Institute for Medical Research and Occupational Health, Ksaverska cesta 2, Zagreb, Croatia*

ADMET & DMPK **14** (2026) 3292; https://doi.org/10.5599/admet.3292

NMR spectra

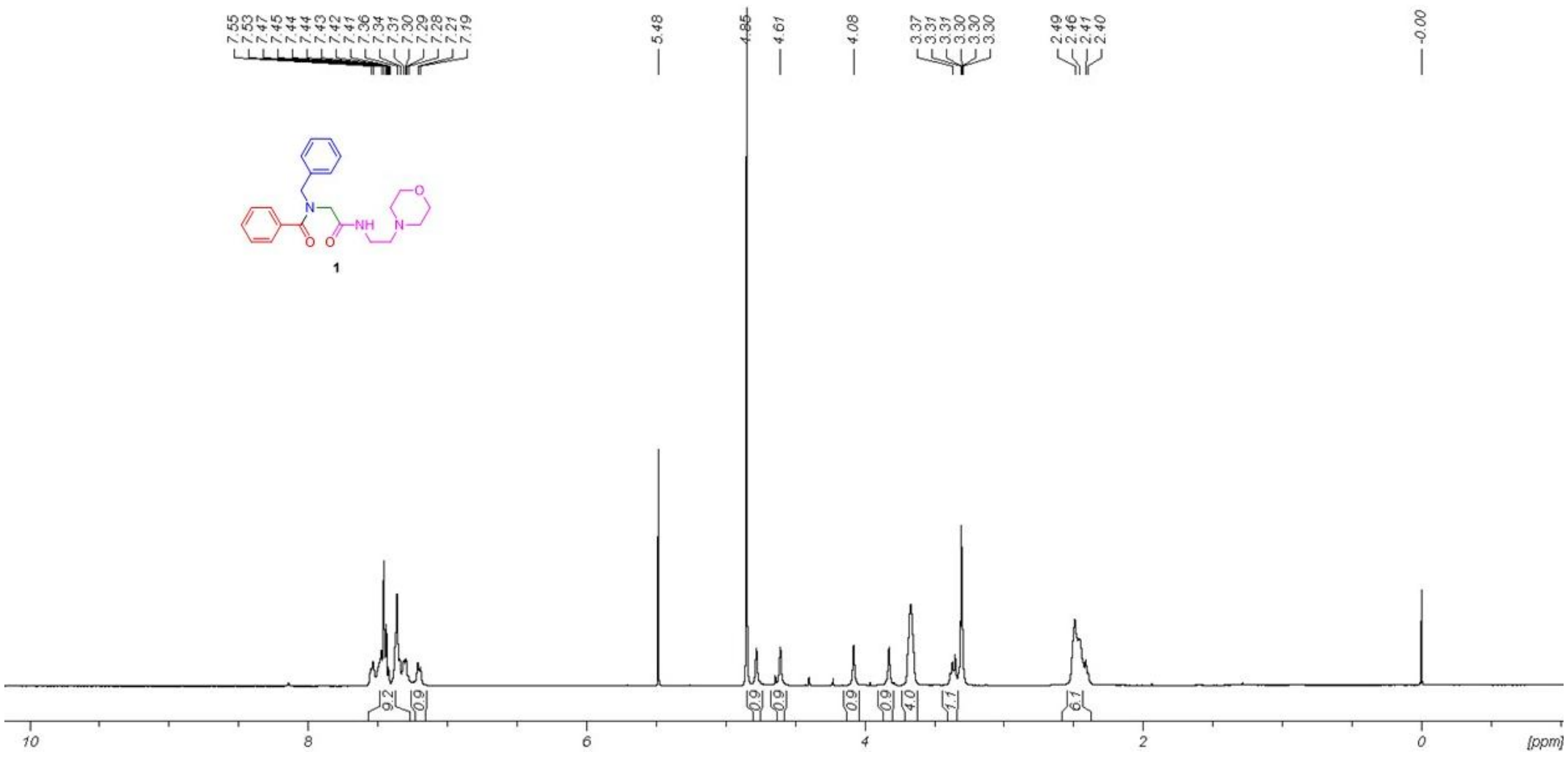

Figure S1. ^{1}H NMR (400 MHz, CD_3OH) of compound **1**

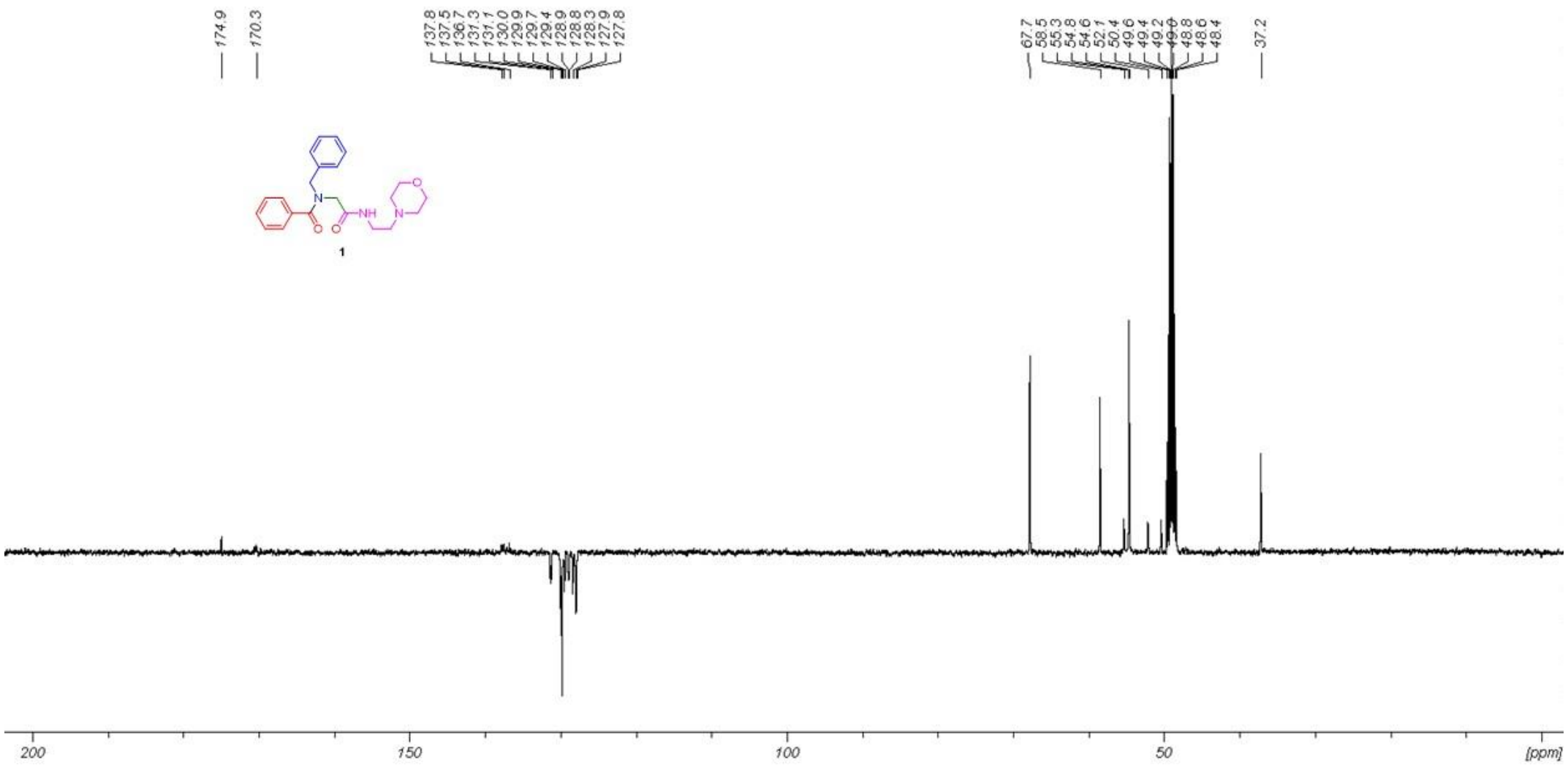

Figure S2. ^{13}C NMR (100 MHz, CD_3OH) of compound **1**

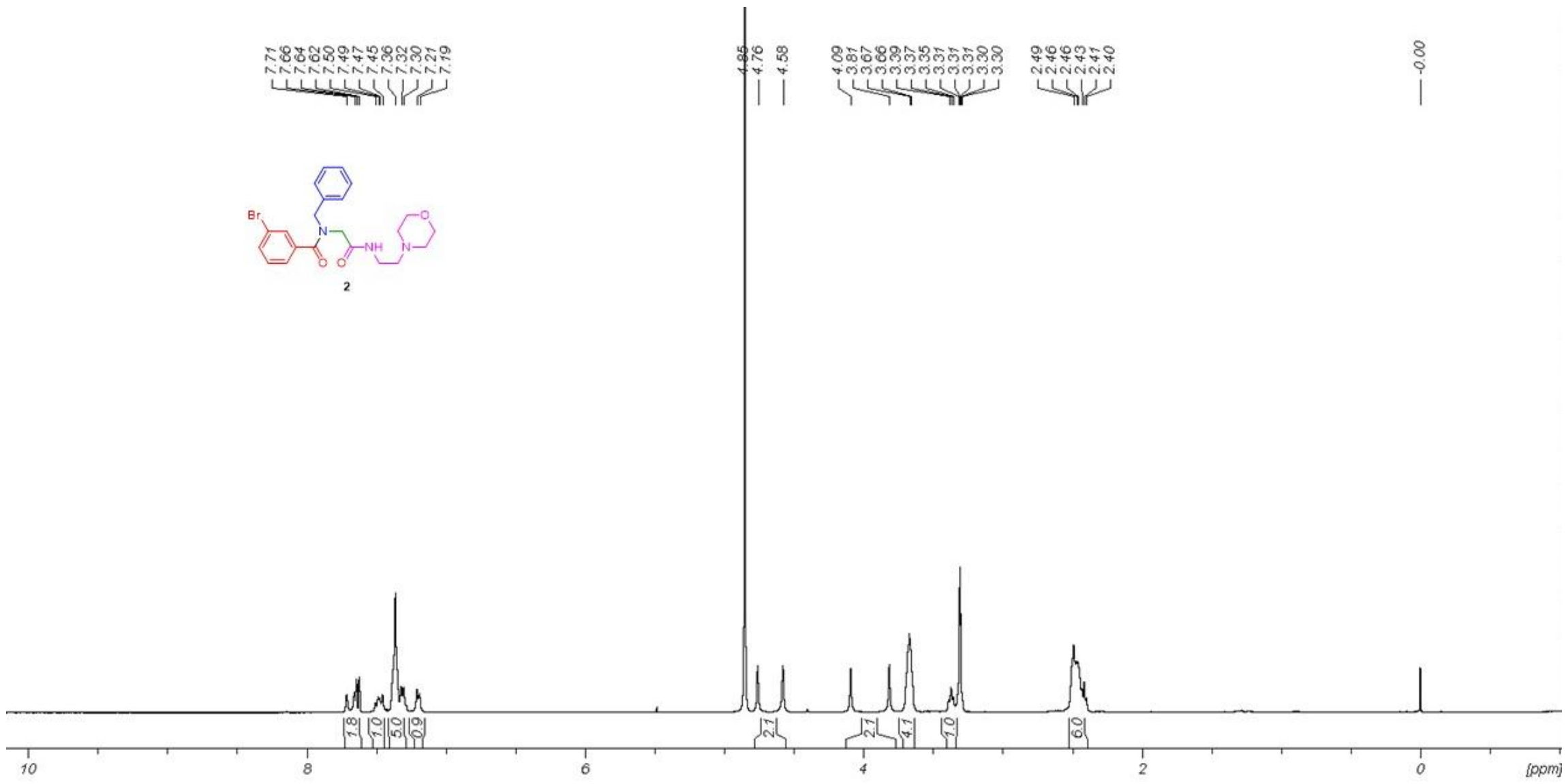

Figure S3. ^{1}H NMR (400 MHz, CD_3OH) of compound **2**

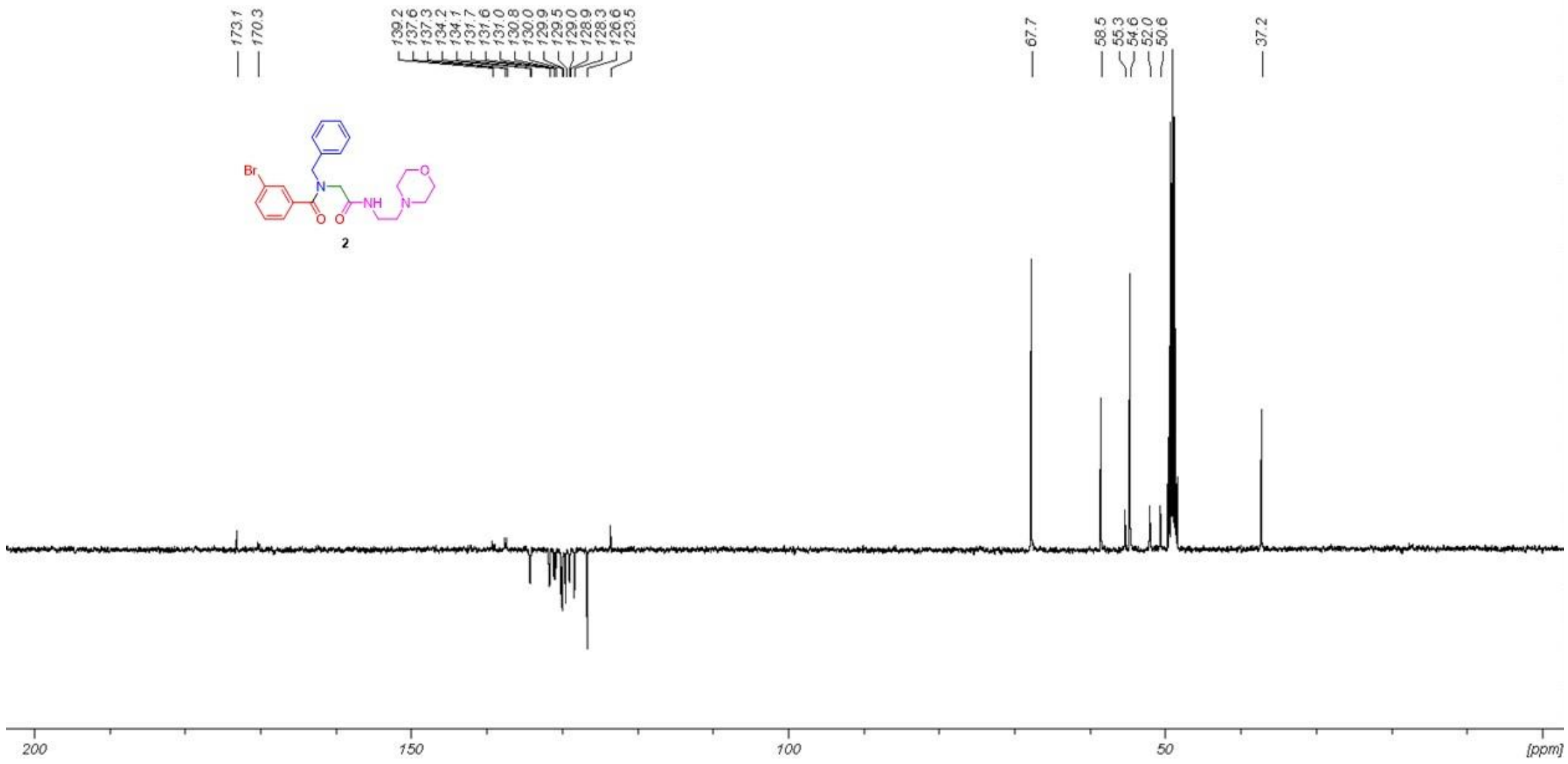

Figure S4. ^{13}C NMR (100 MHz, CD_3OH) of compound **2**

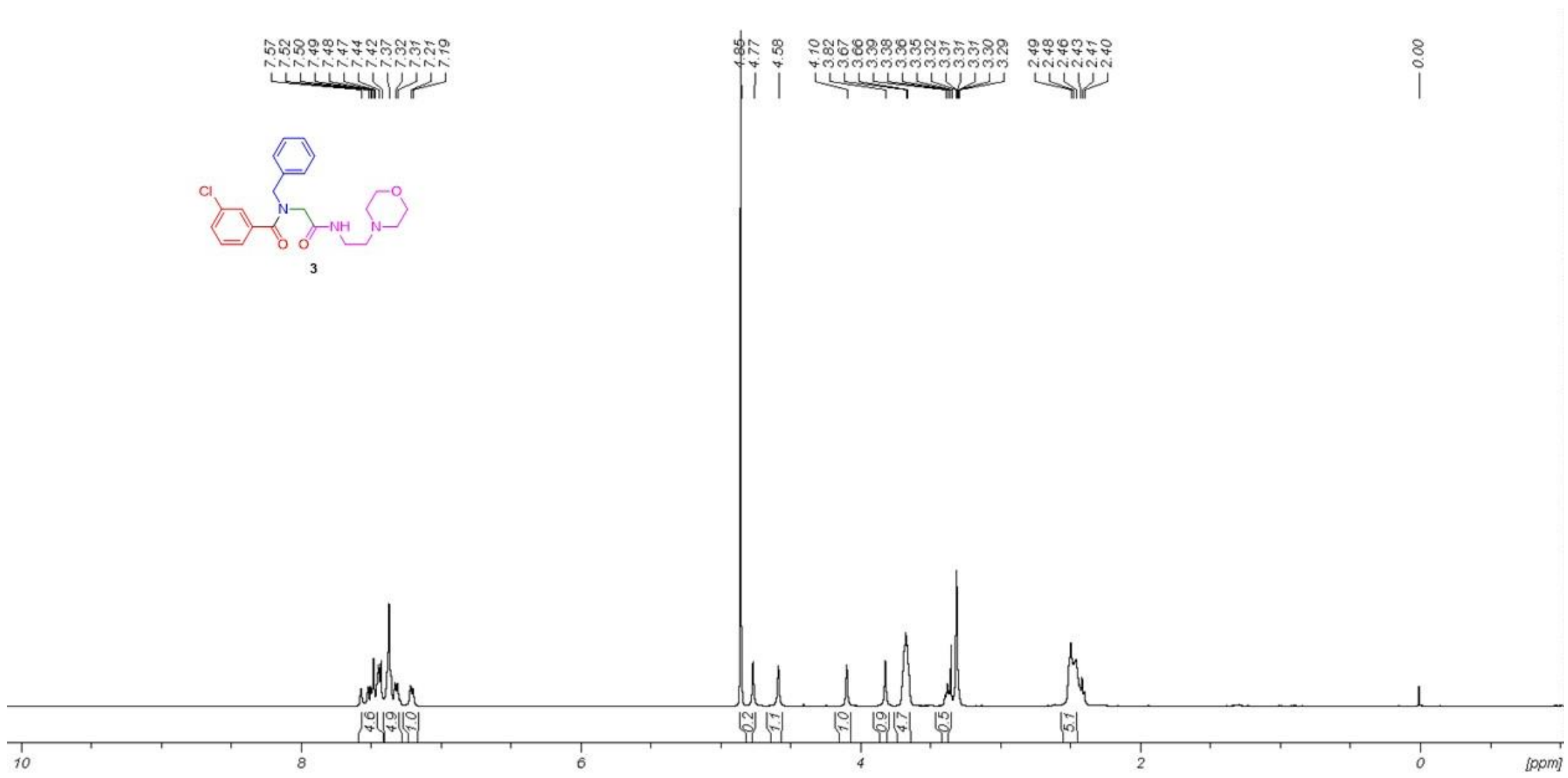

Figure S5. ^{1}H NMR (400 MHz, CD_3OH) of compound **3**

CC BY

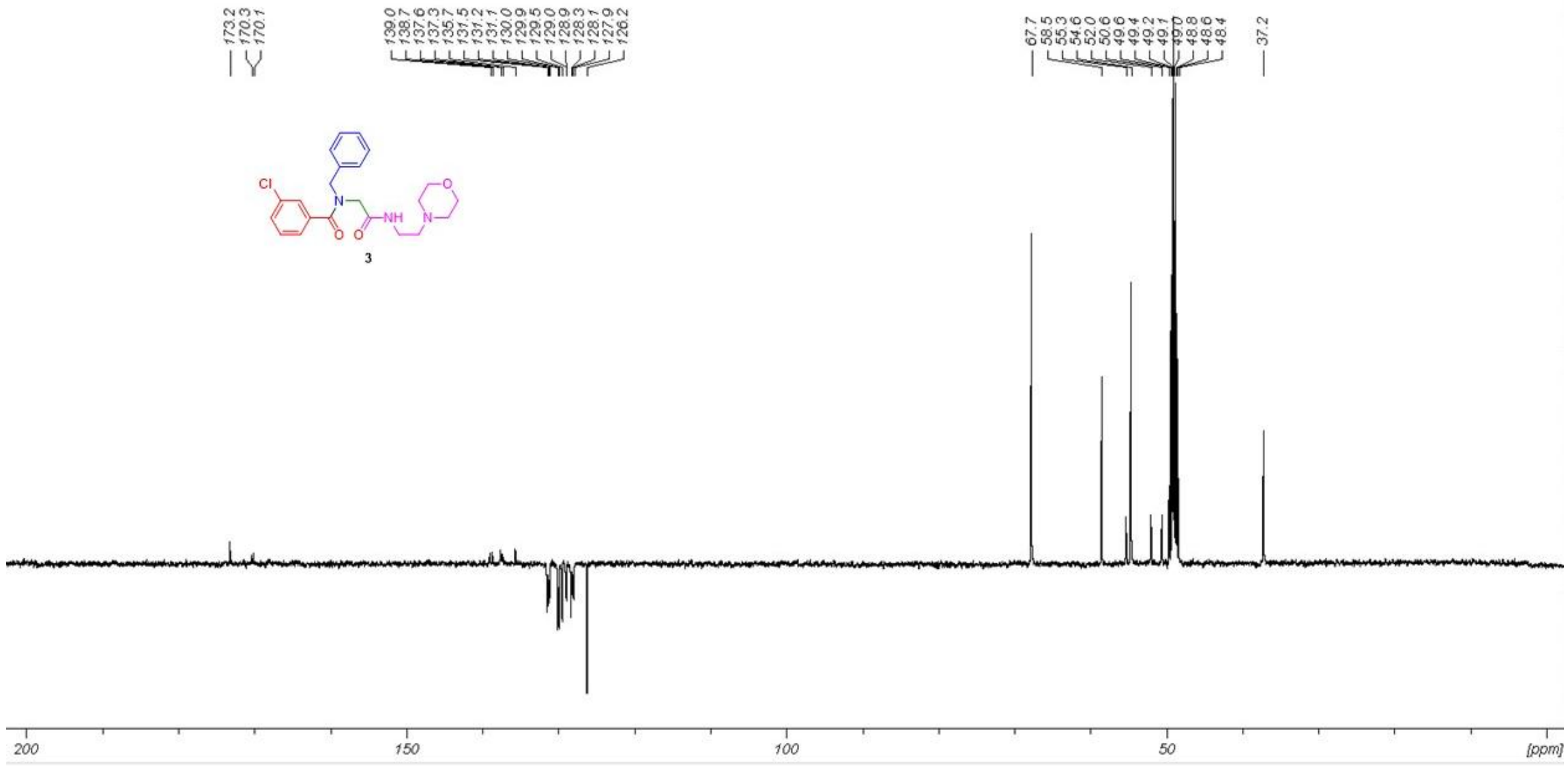

Figure S6. ^{13}C NMR (100 MHz, CD_3OH) of compound **3**

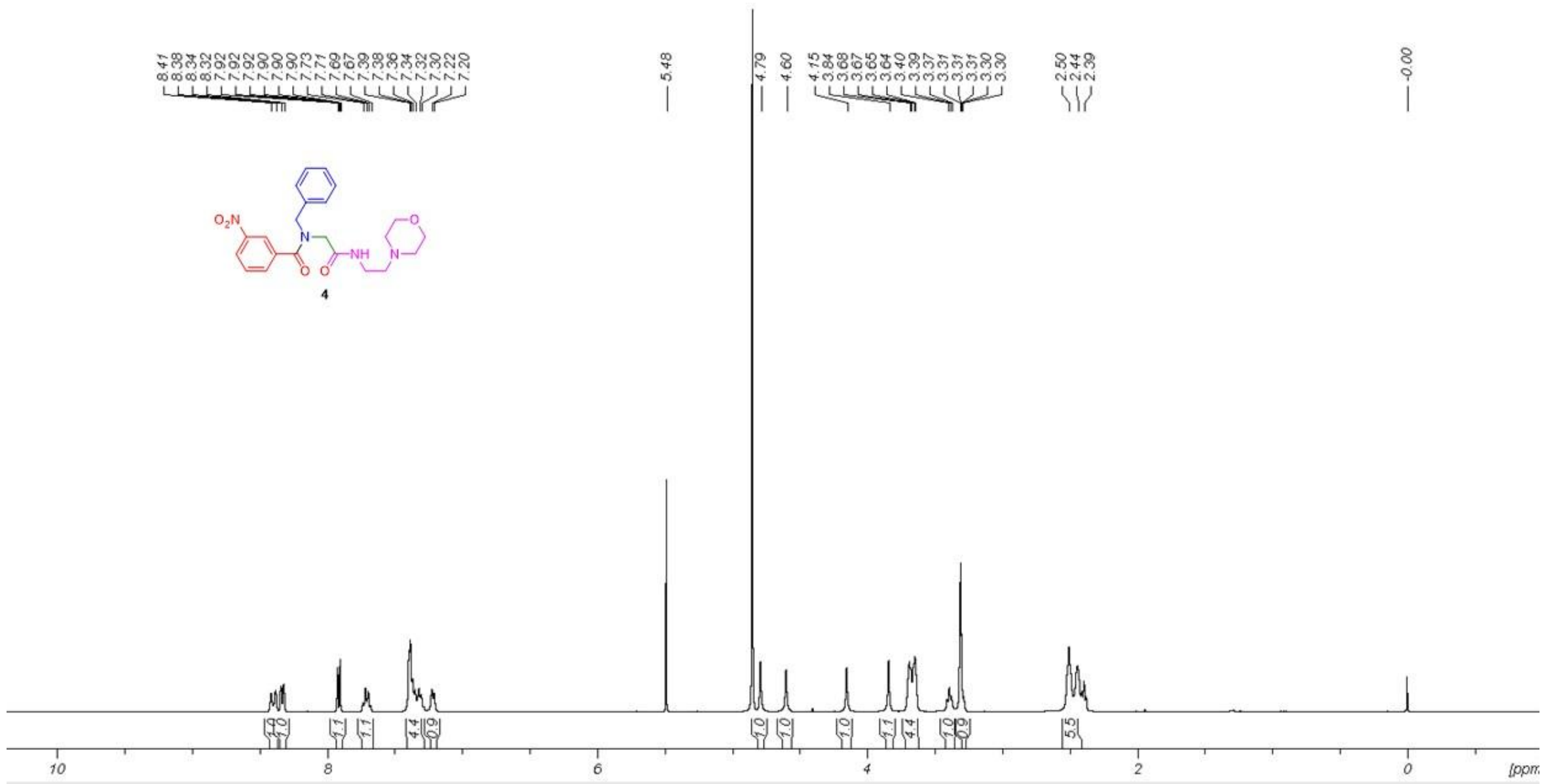

Figure S7. ^{1}H NMR (400 MHz, CD_3OH) of compound **4**

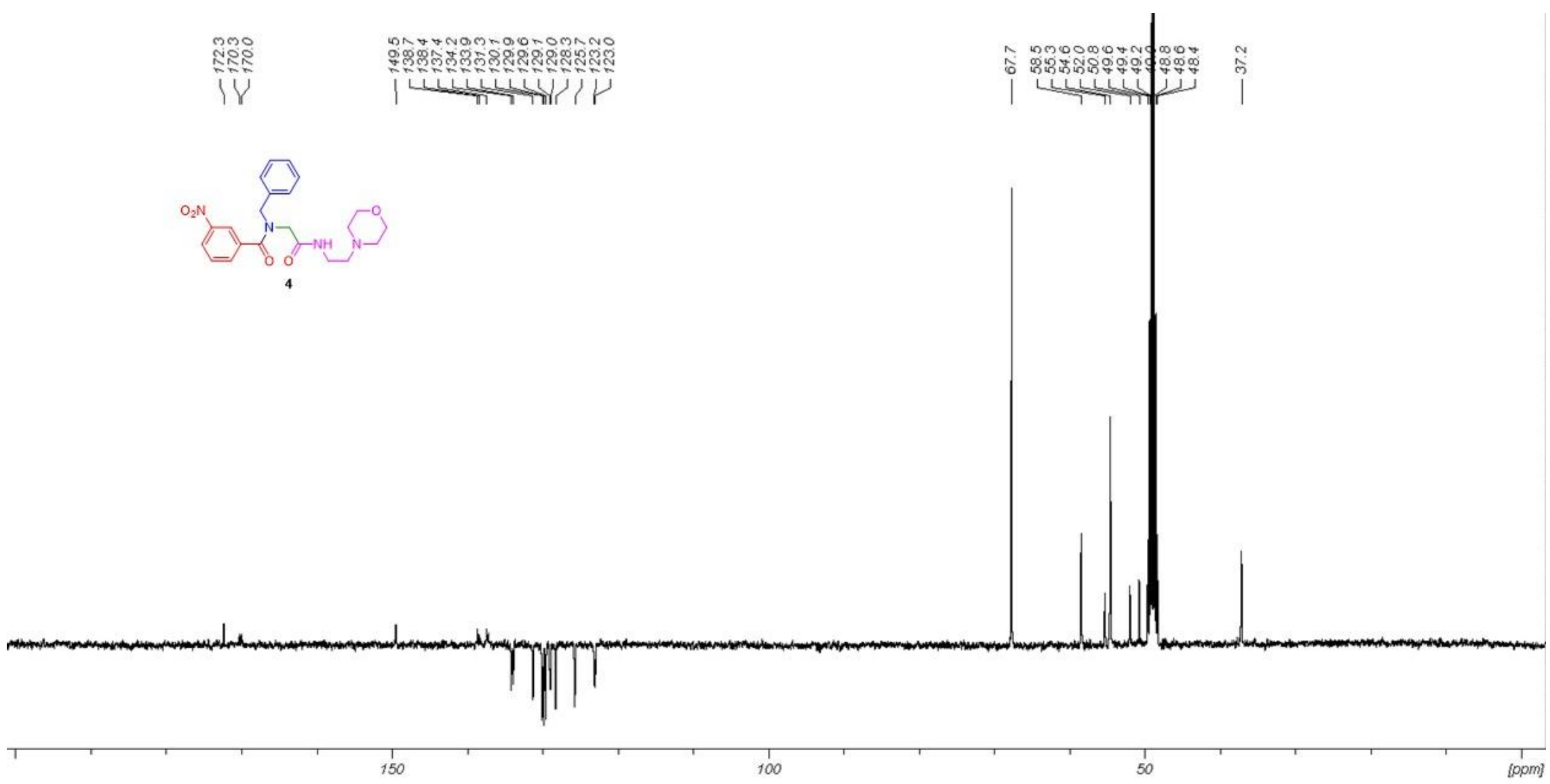

Figure S8. ^{13}C NMR (100 MHz, CD_3OH) of compound **4**

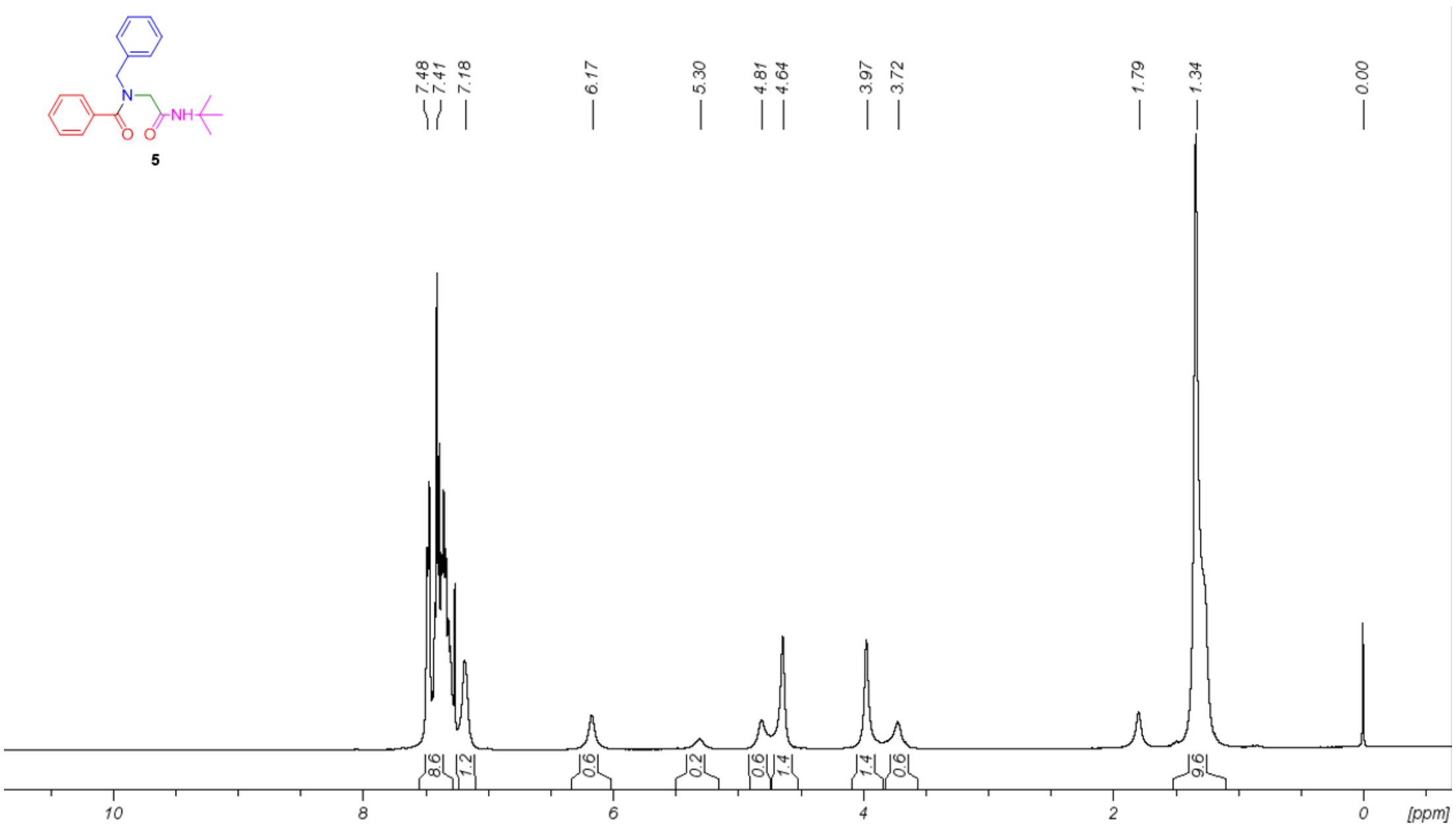

Figure S9. ^{1}H NMR (400 MHz, CDCl$_3$) of compound **5**

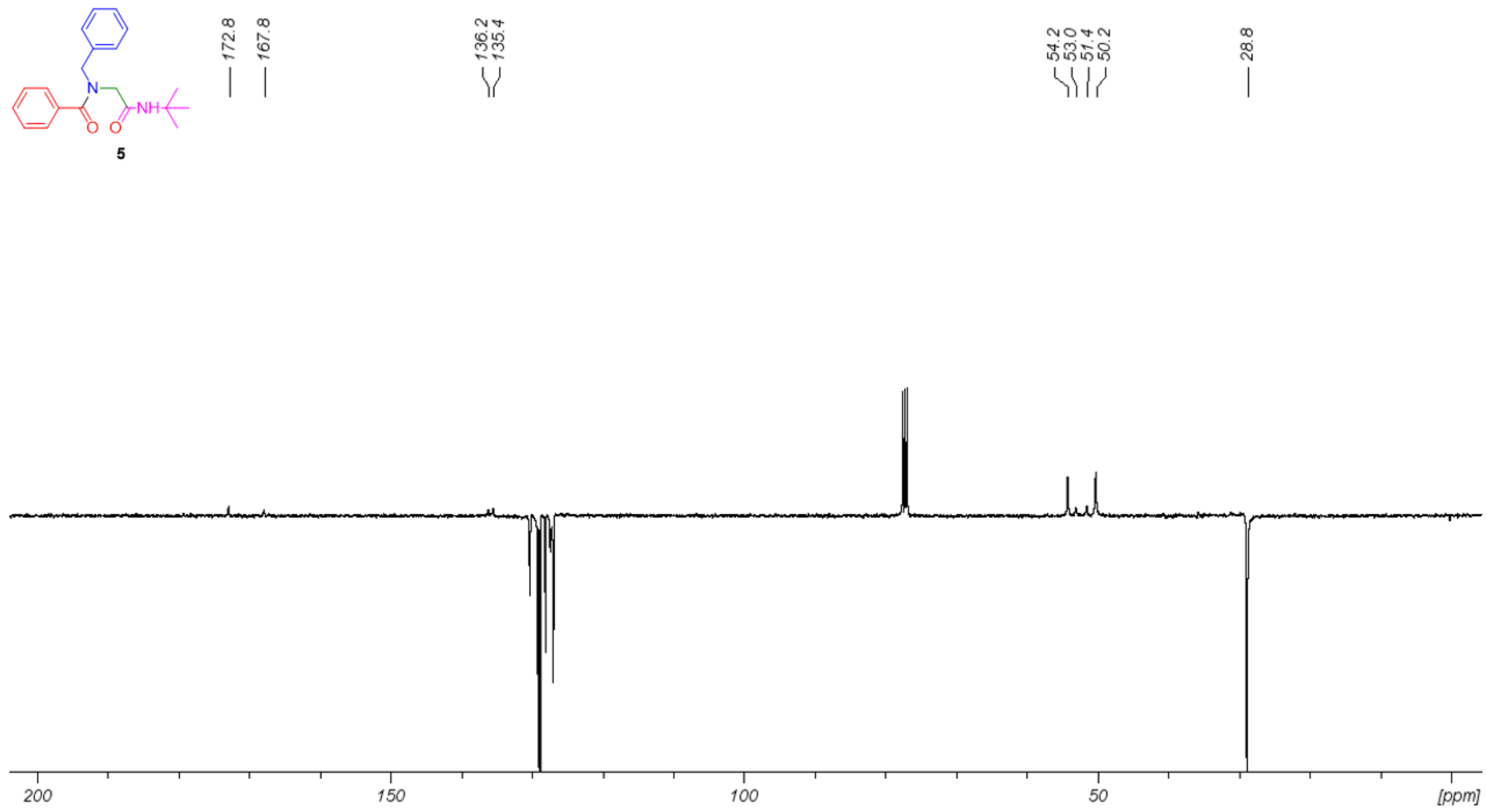

Figure S10. ^{13}C NMR (100 MHz, CDCl$_3$) of compound **5**

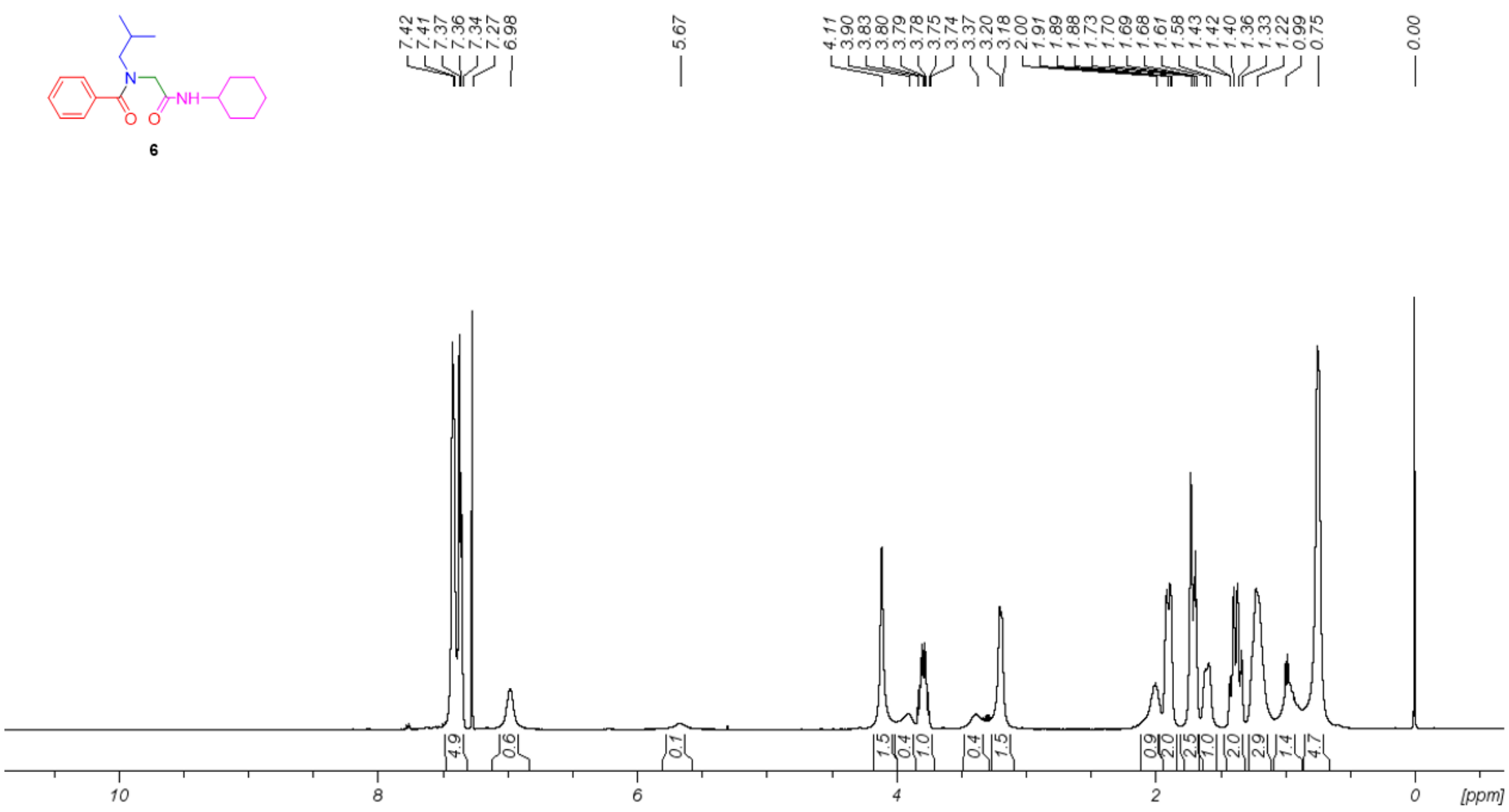

Figure S11. ^{1}H NMR (400 MHz, CDCl$_3$) of compound **6**

CC BY

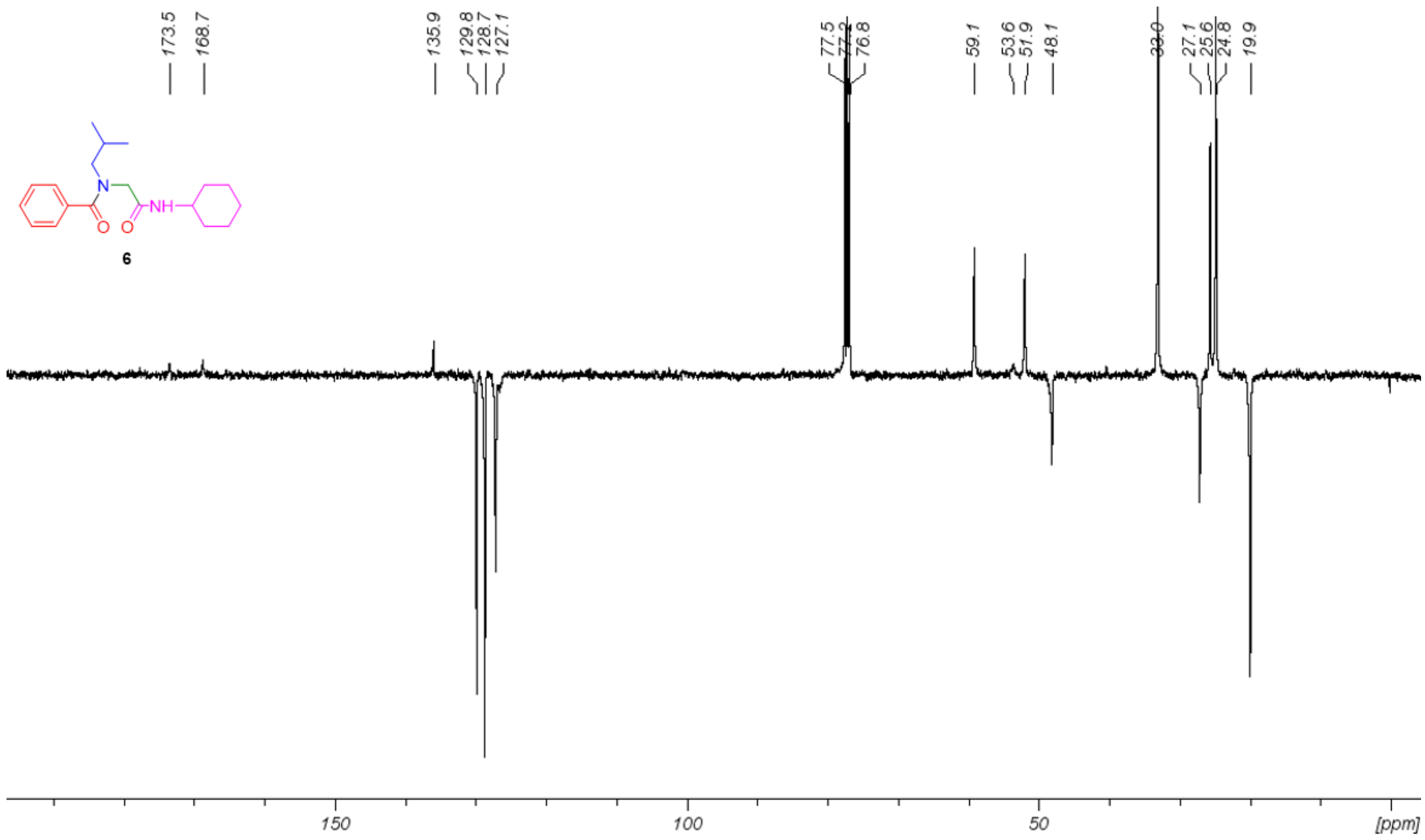

Figure S12. ^{13}C NMR (100 MHz, $CDCl_3$) of compound **6**

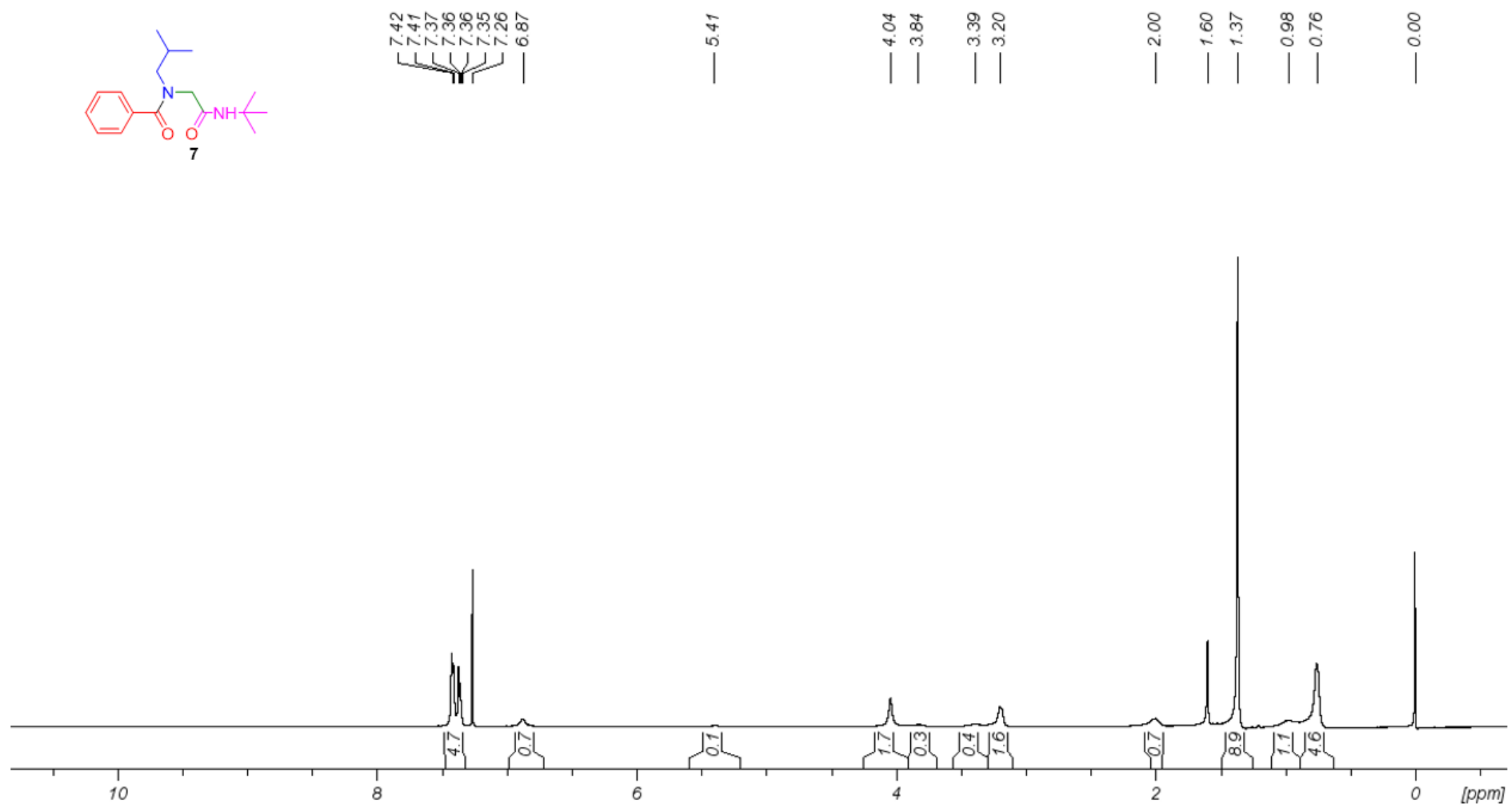

Figure S13. ^{1}H NMR (400 MHz, $CDCl_3$) of compound **7**

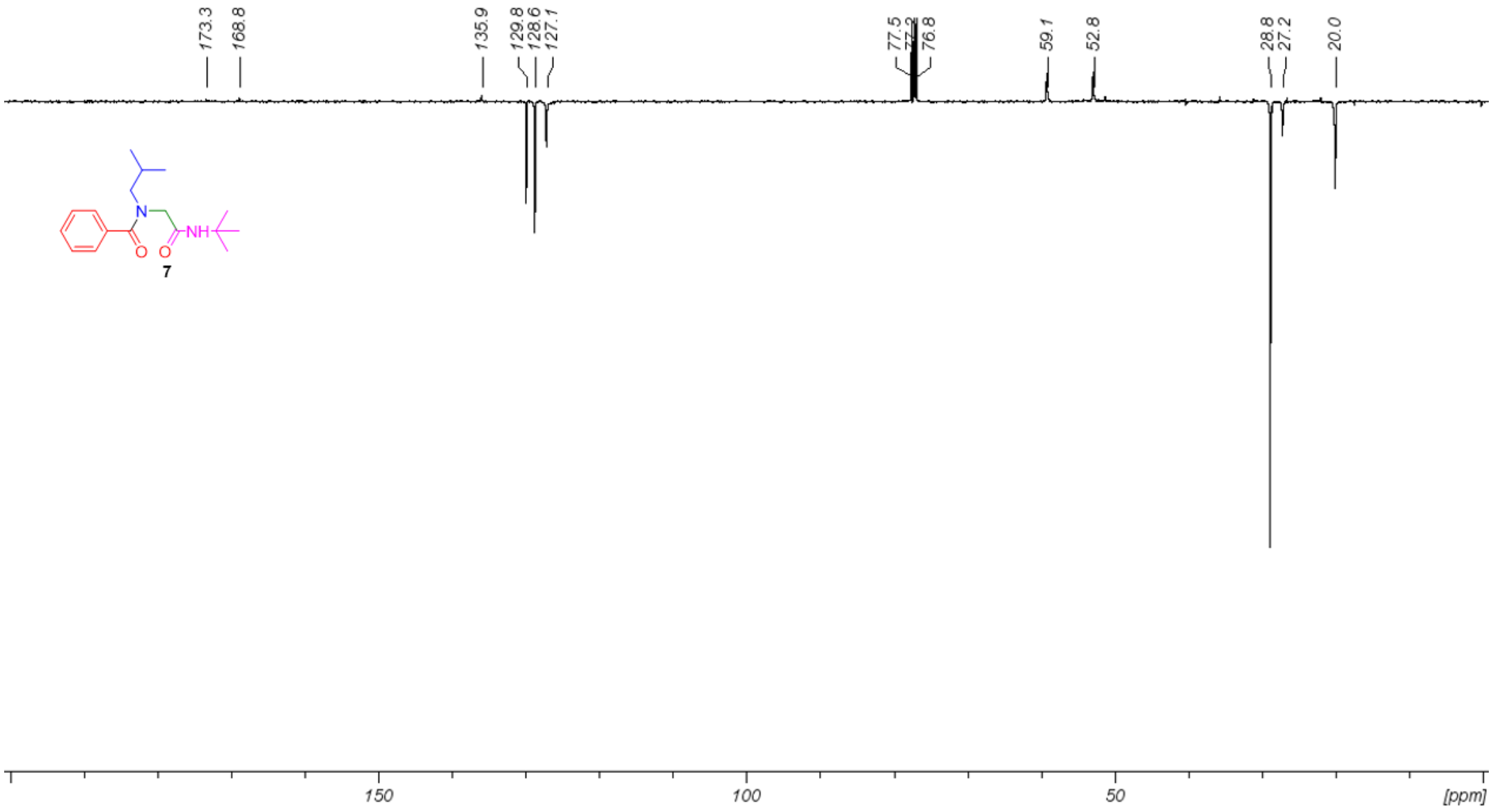

Figure S14. ^{13}C NMR (100 MHz, $CDCl_3$) of compound **7**

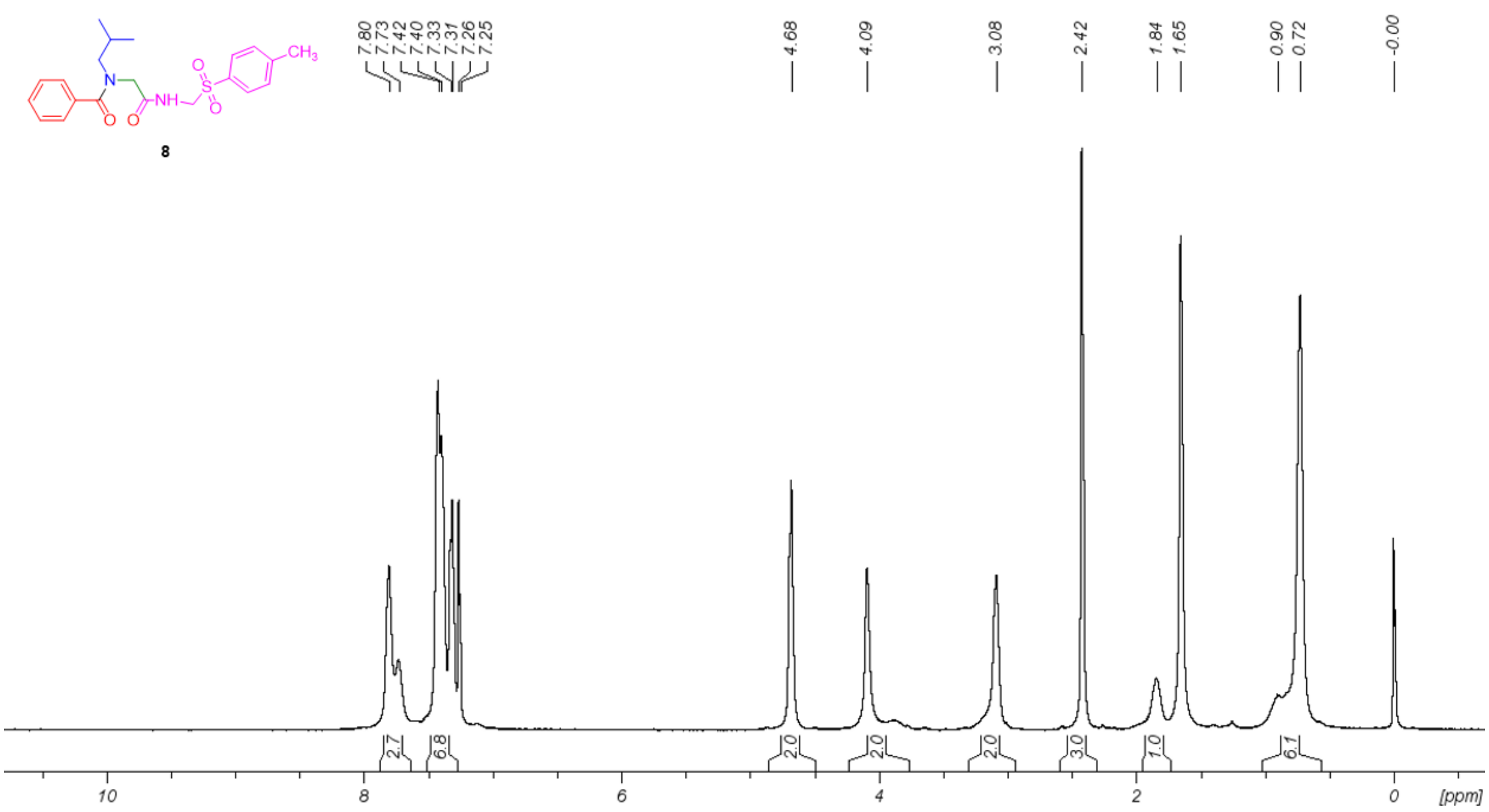

Figure S15. ^{1}H NMR (400 MHz, $CDCl_3$) of compound **8**

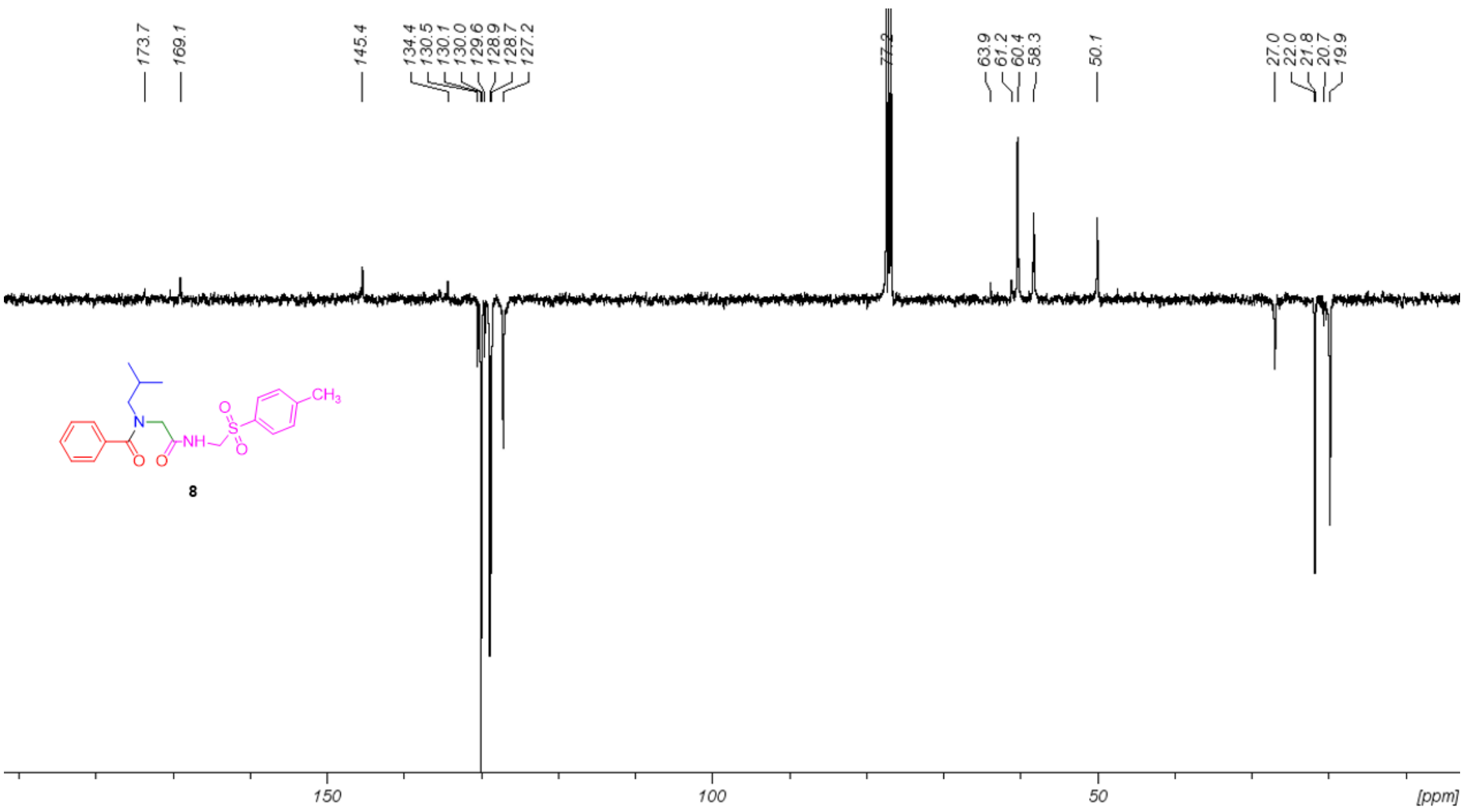

Figure S16. ^{13}C NMR (100 MHz, $CDCl_3$) of compound **8**

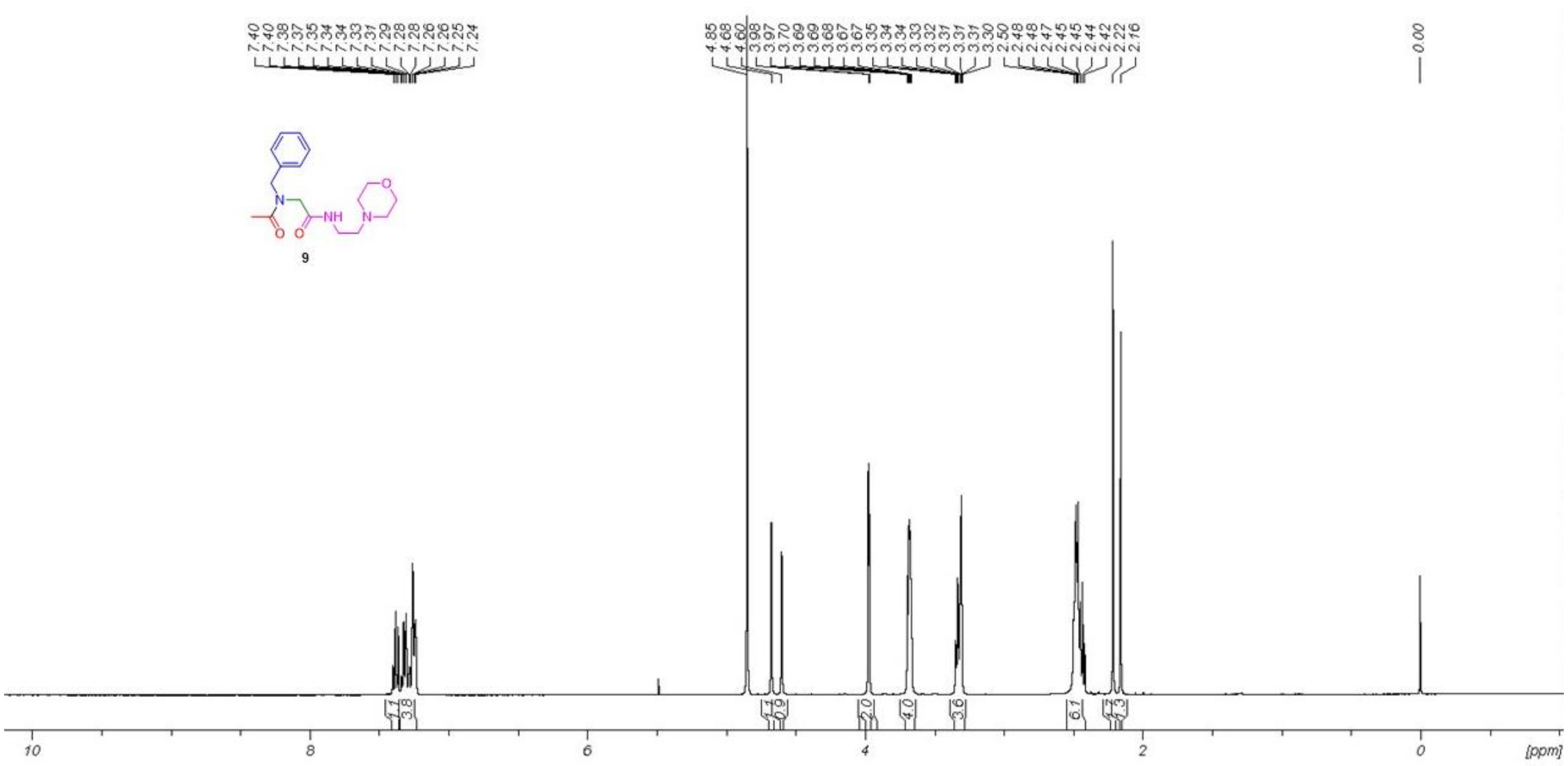

Figure S17. ^{1}H NMR (400 MHz, CD_3OH) of compound **9**

(cc) BY

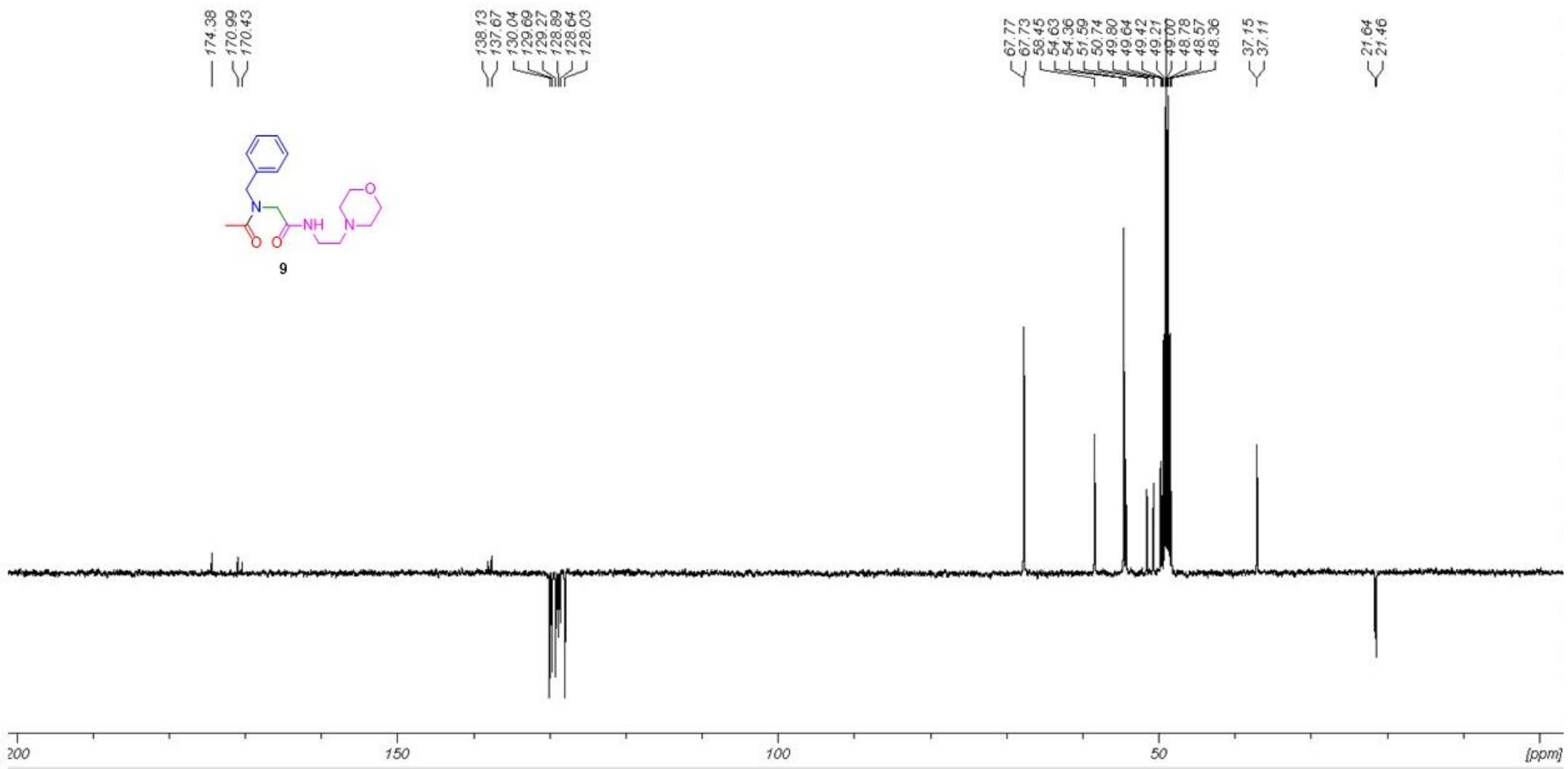

Figure S18. ^{13}C NMR (100 MHz, CD_3OH) of compound **9**

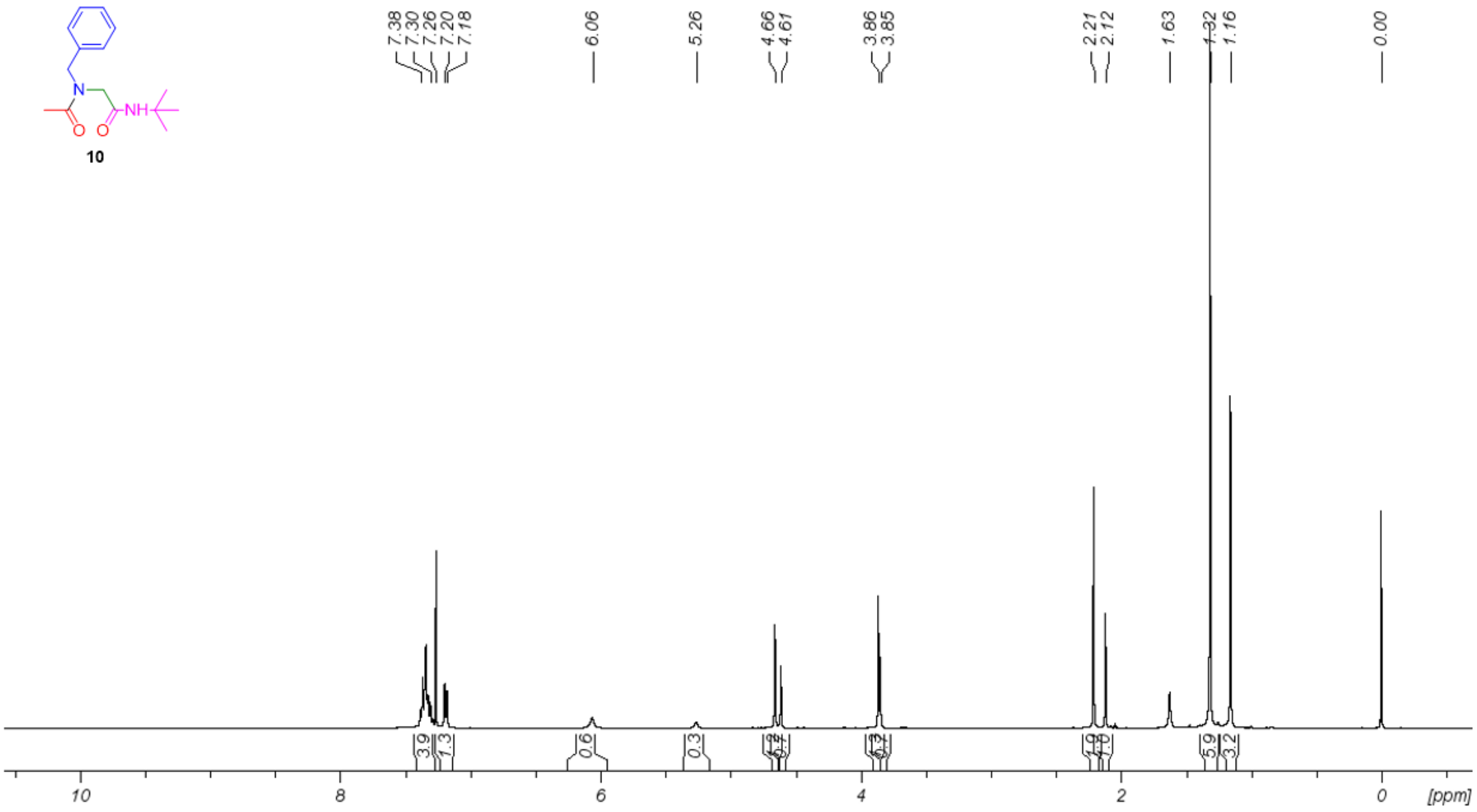

Figure S19. ^{1}H NMR (400 MHz, $CDCl_3$) of compound **10**

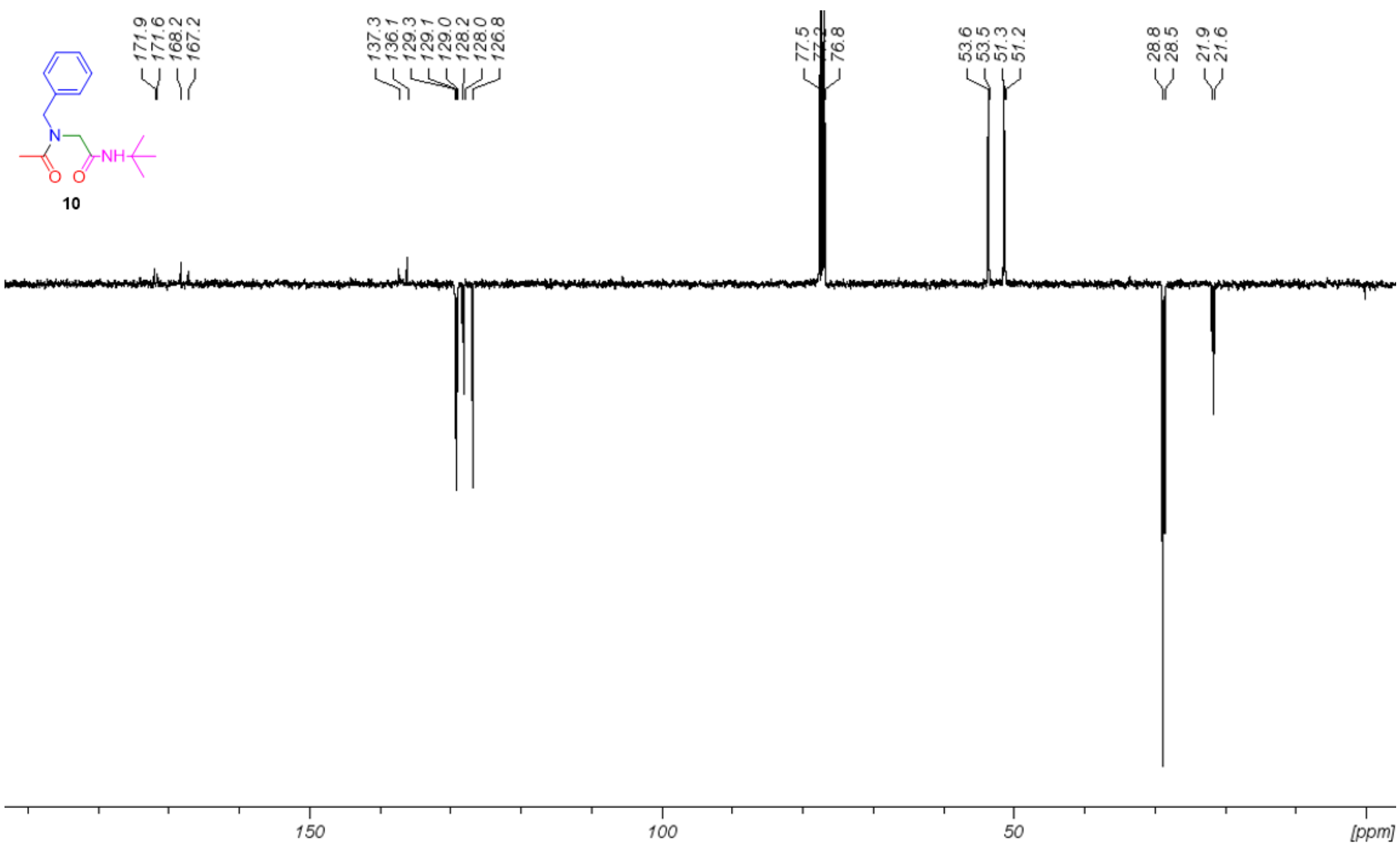

Figure S20. ^{13}C NMR (100 MHz, $CDCl_3$) of compound **10**

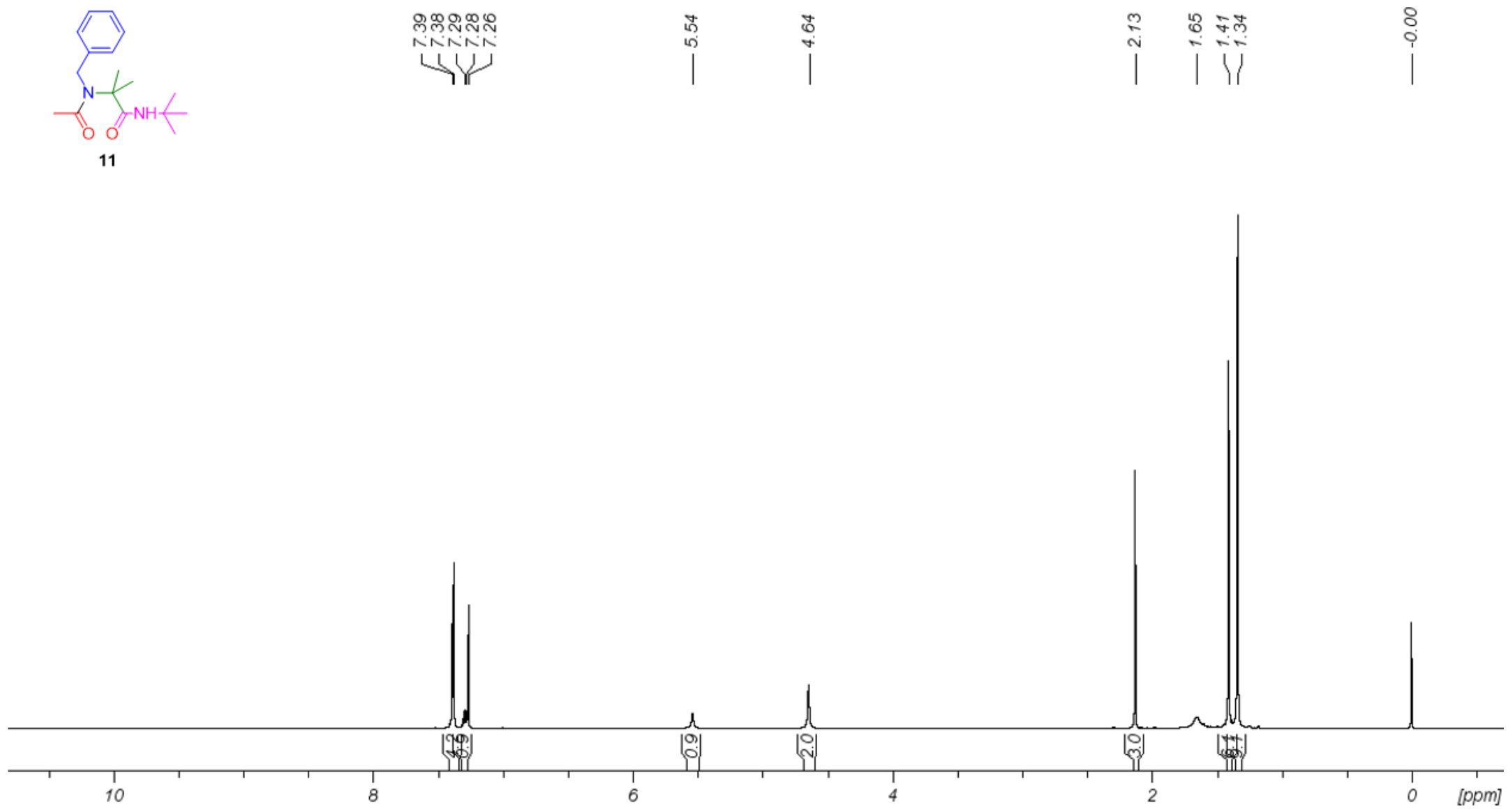

Figure S21. ^{1}H NMR (400 MHz, $CDCl_3$) of compound **11**

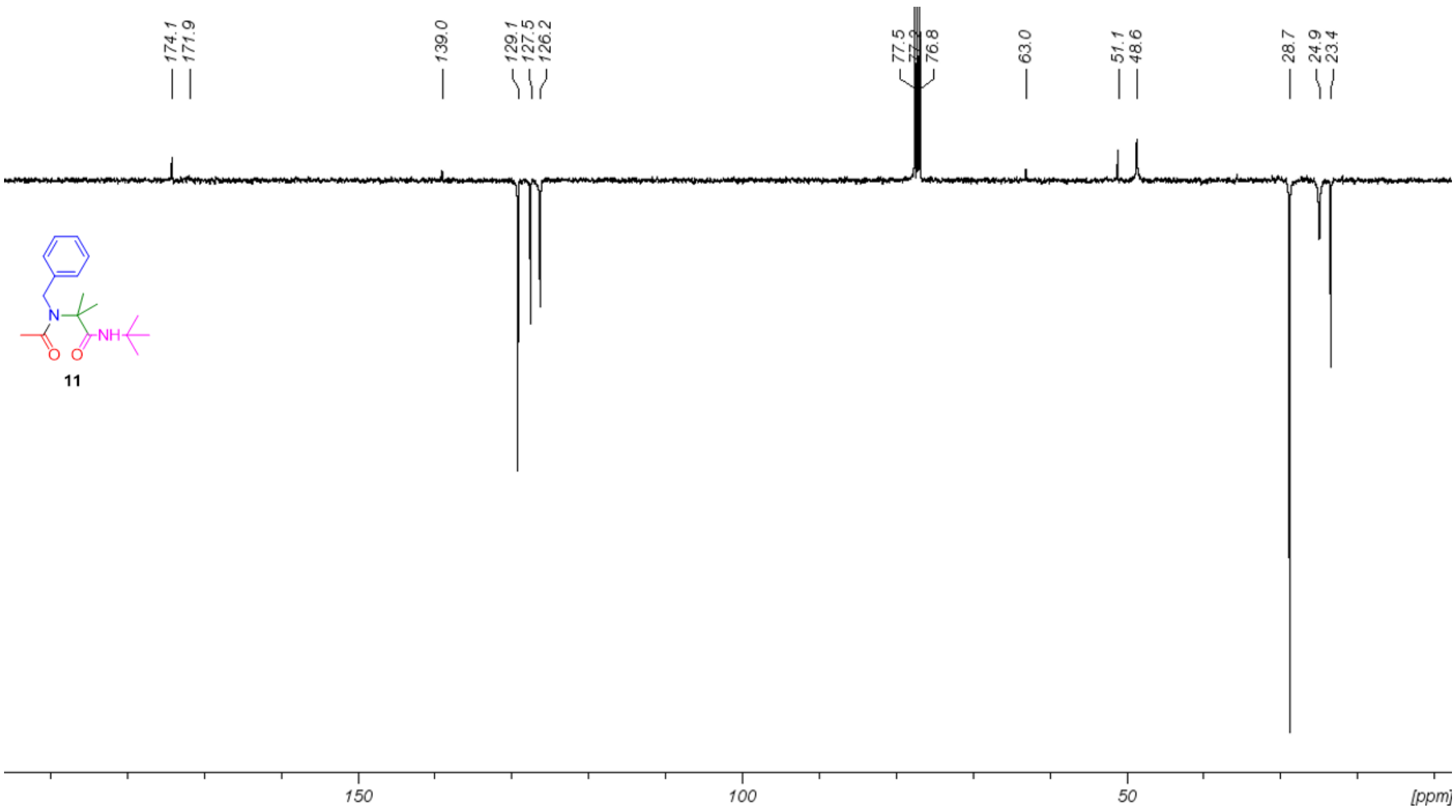

Figure S22. ^{13}C NMR (100 MHz, $CDCl_3$) of compound **11**

HPLC chromatograms

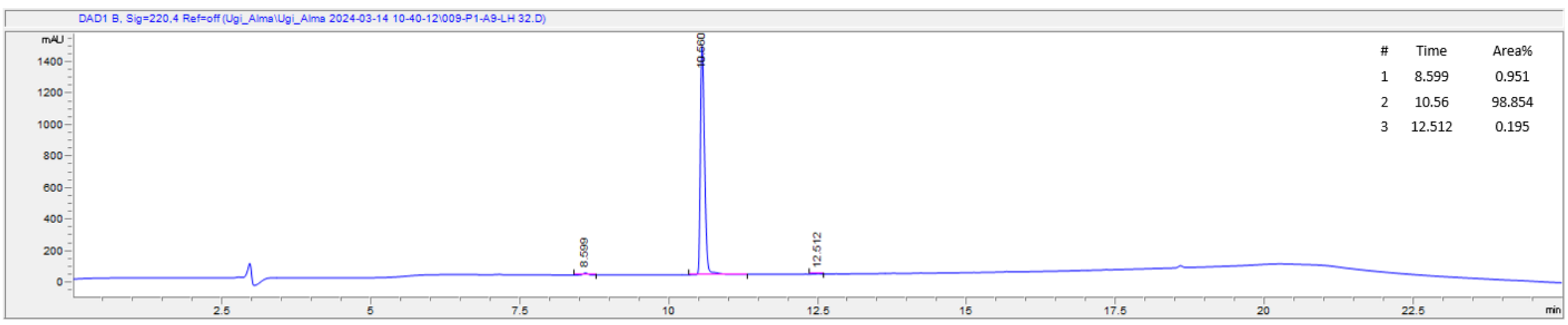

Figure S23. HPLC chromatogram of compound **1**

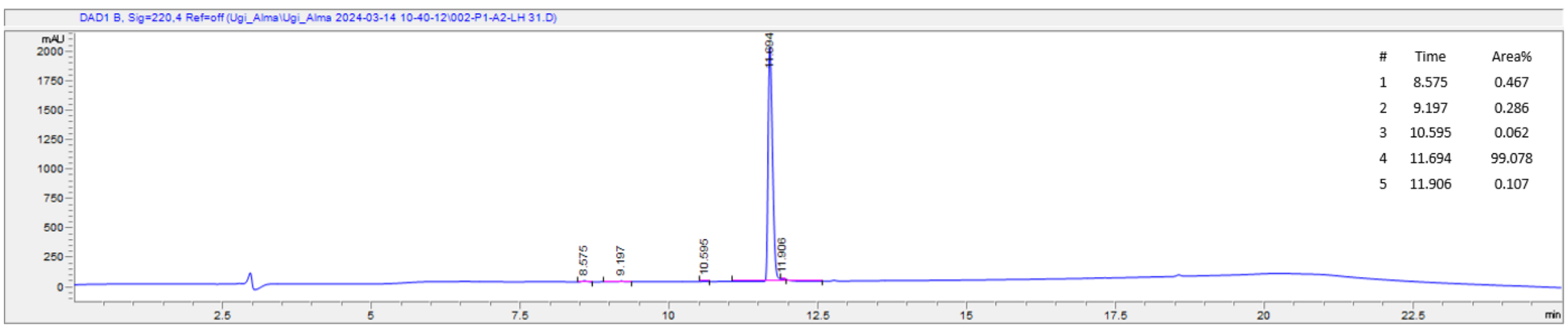

Figure S24. HPLC chromatogram of compound **2**

CC BY

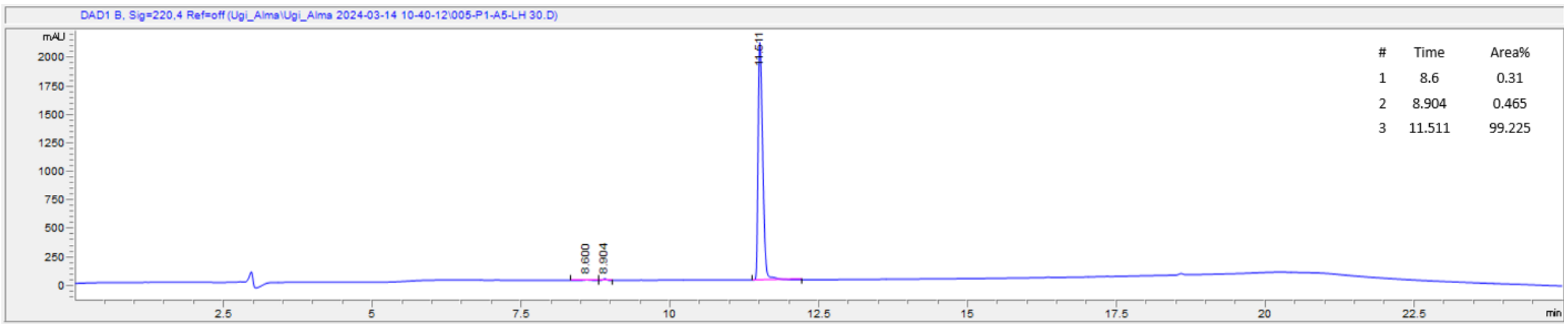

Figure S25. HPLC chromatogram of compound **3**

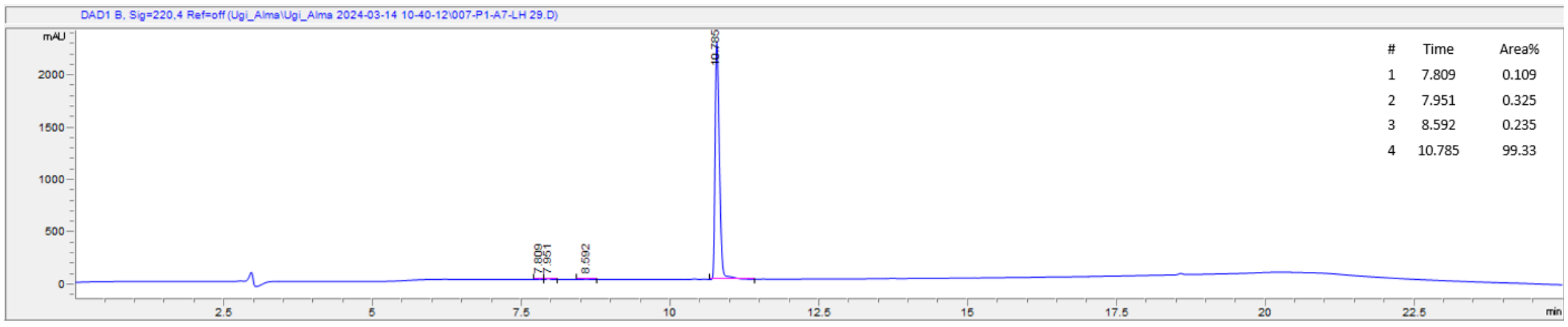

Figure S26. HPLC chromatogram of compound **4**

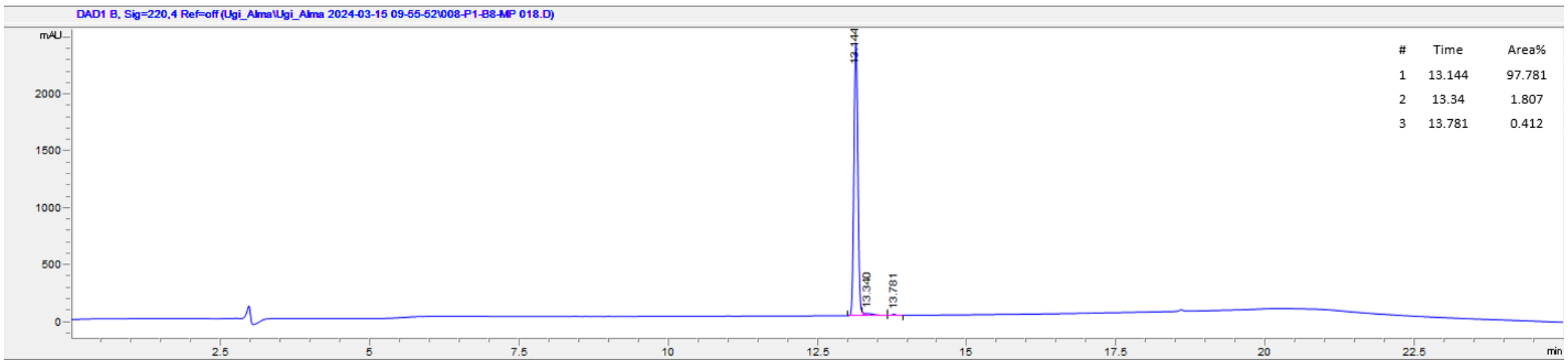

Figure S27. HPLC chromatogram of compound **5**

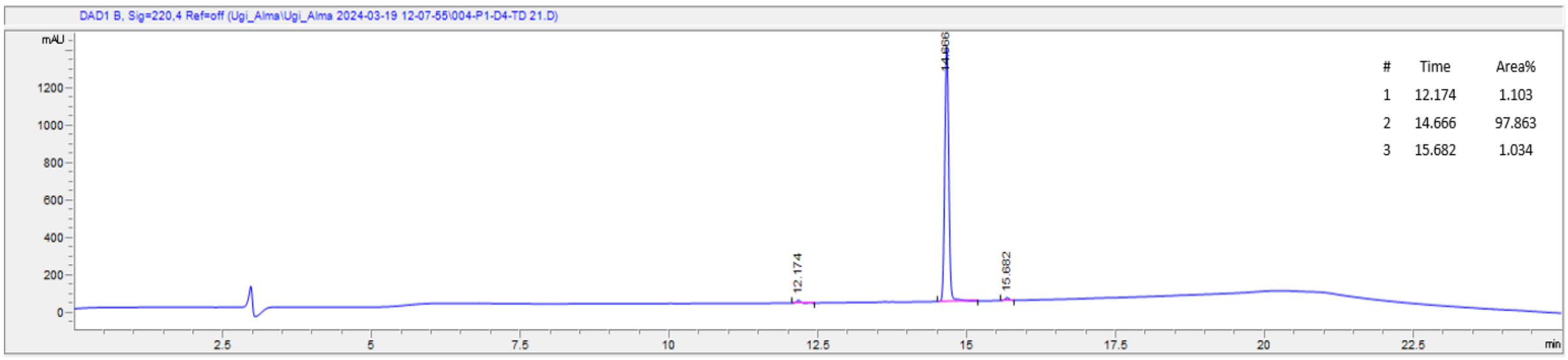

Figure S28. HPLC chromatogram of compound **6**

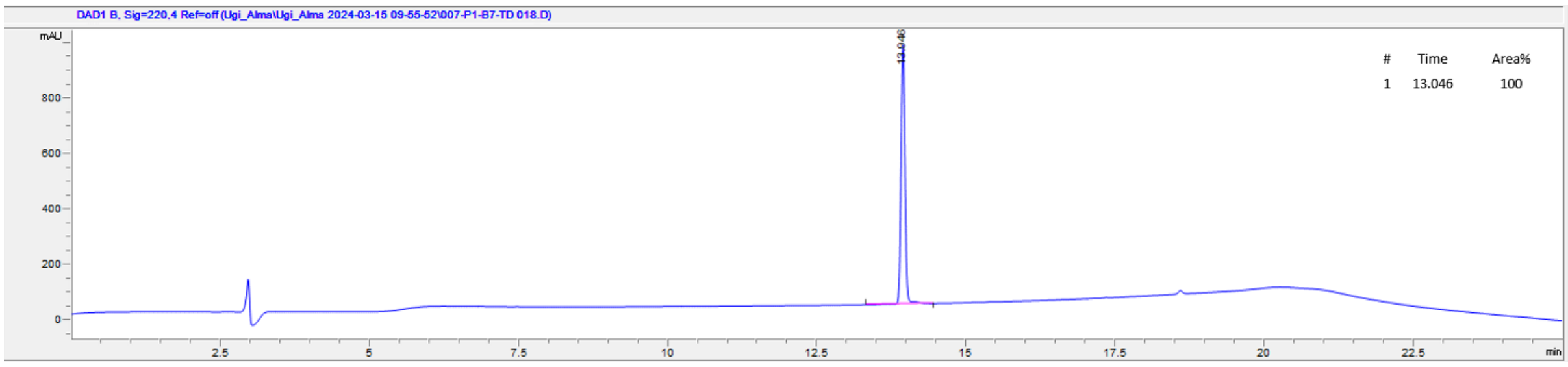

Figure S29. HPLC chromatogram of compound **7**

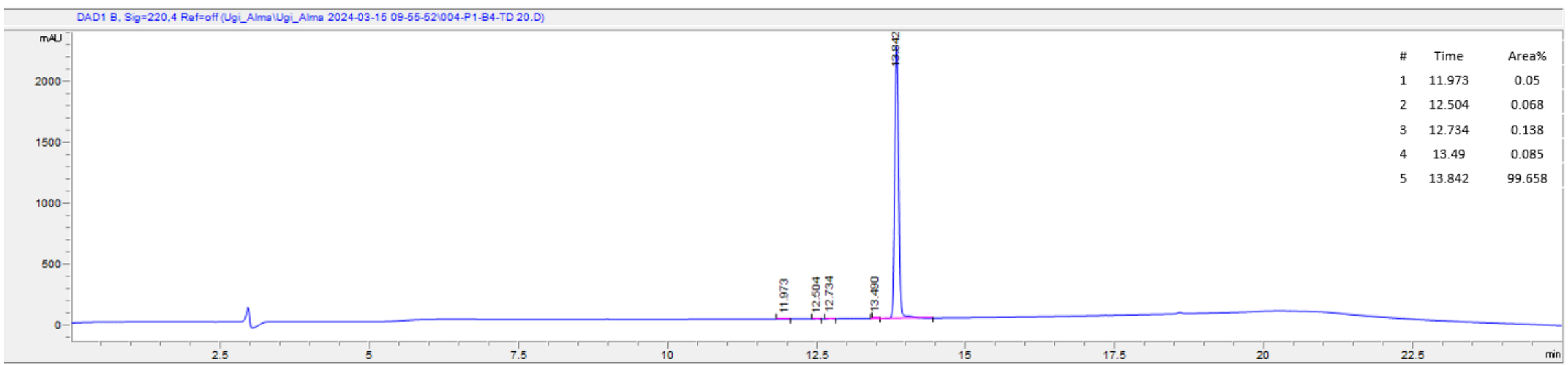

Figure S30. HPLC chromatogram of compound **8**

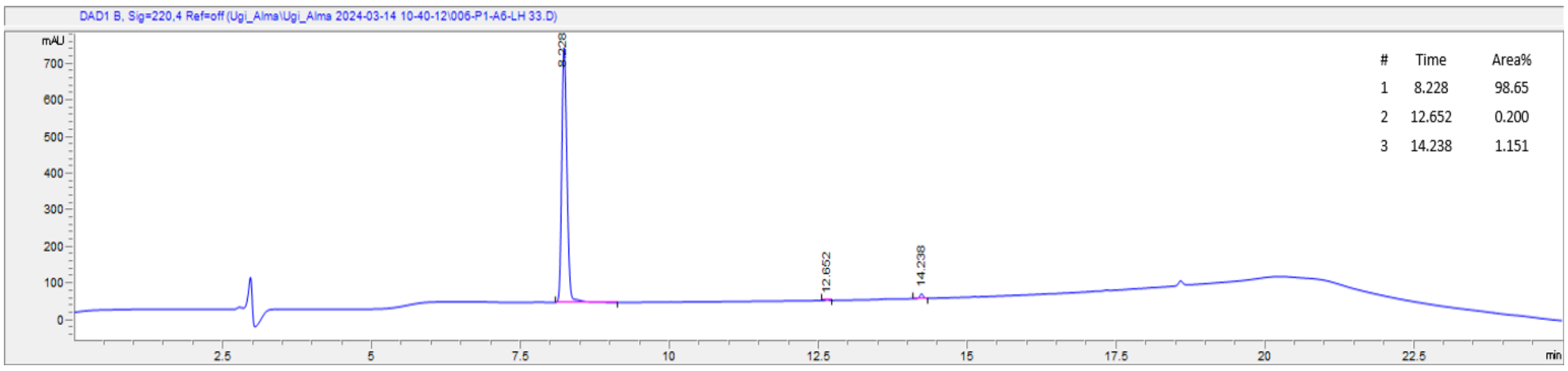

Figure S31. HPLC chromatogram of compound **9**

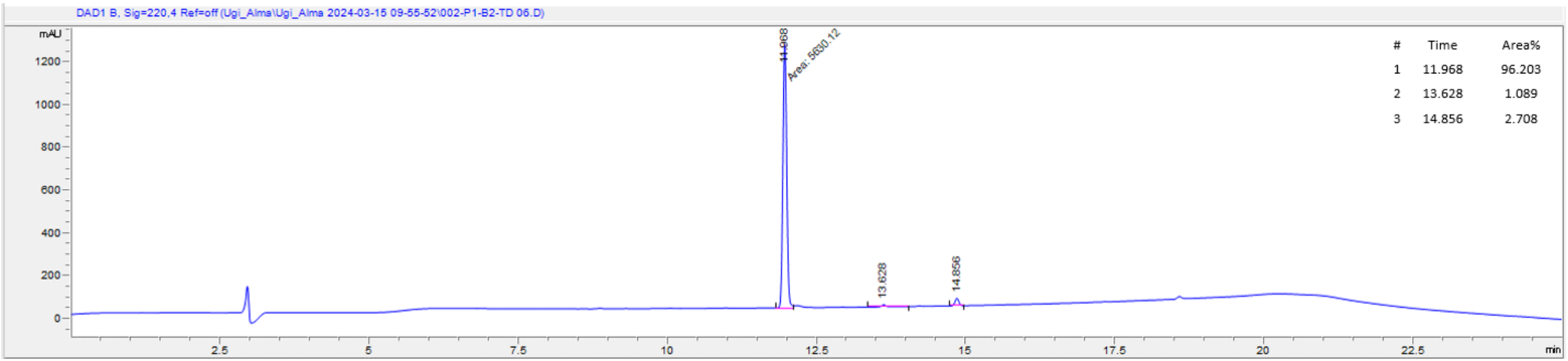

Figure S32. HPLC chromatogram of compound **10**

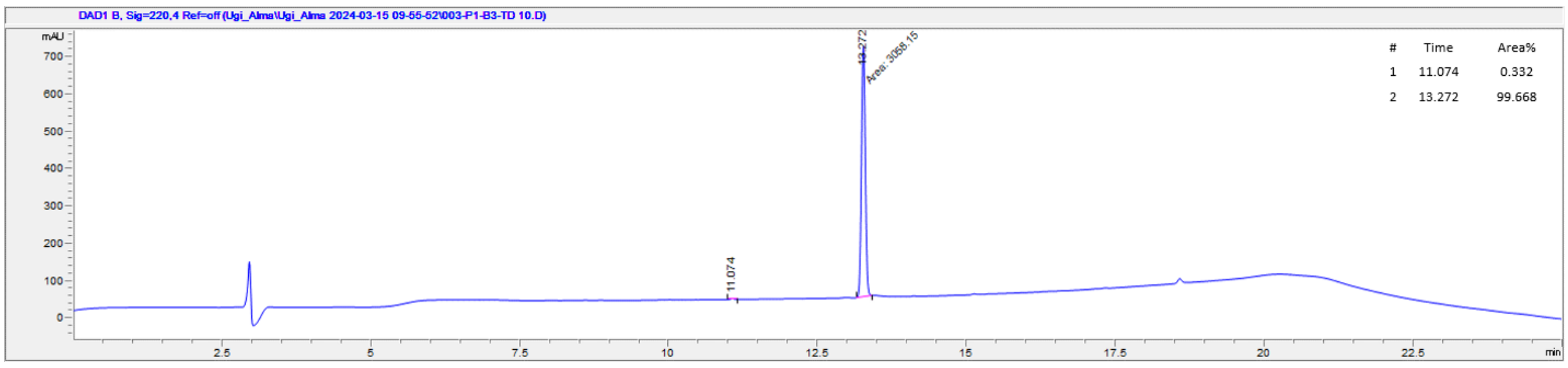

Figure S33. HPLC chromatogram of compound **11**

3.HRMS spectra

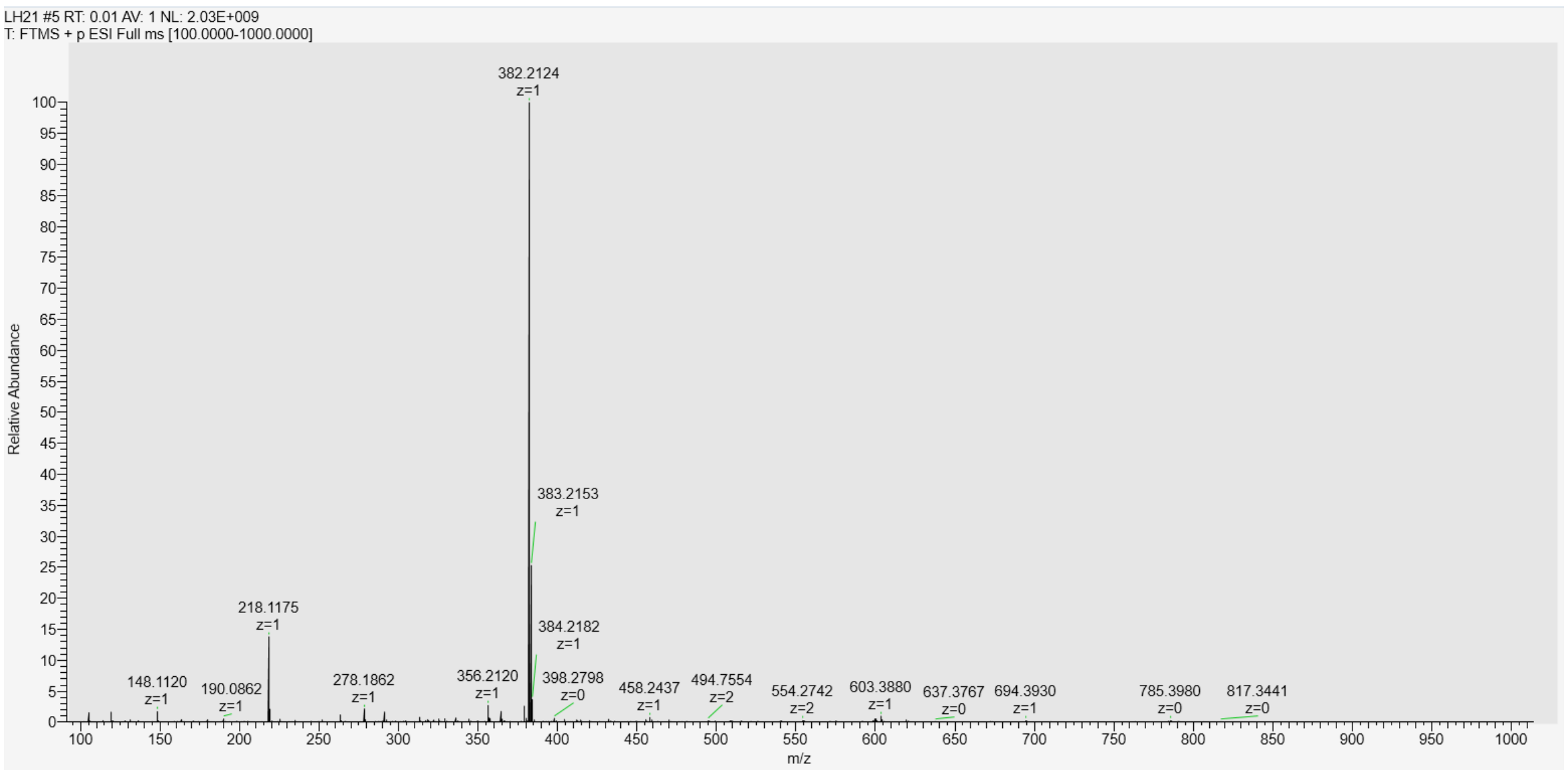

Figure S34. HRMS spectrum of compound **1**

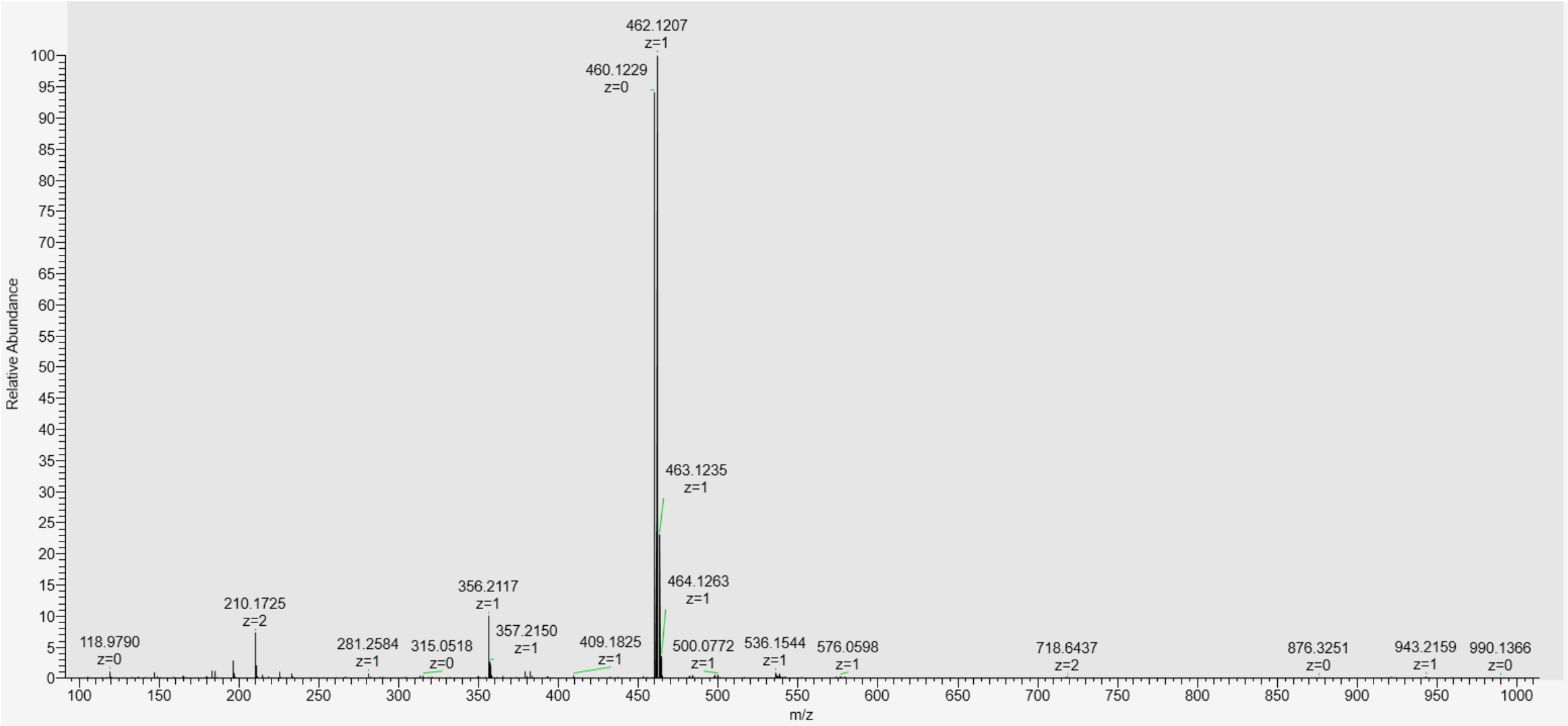

Figure S35. HRMS spectrum of compound **2**

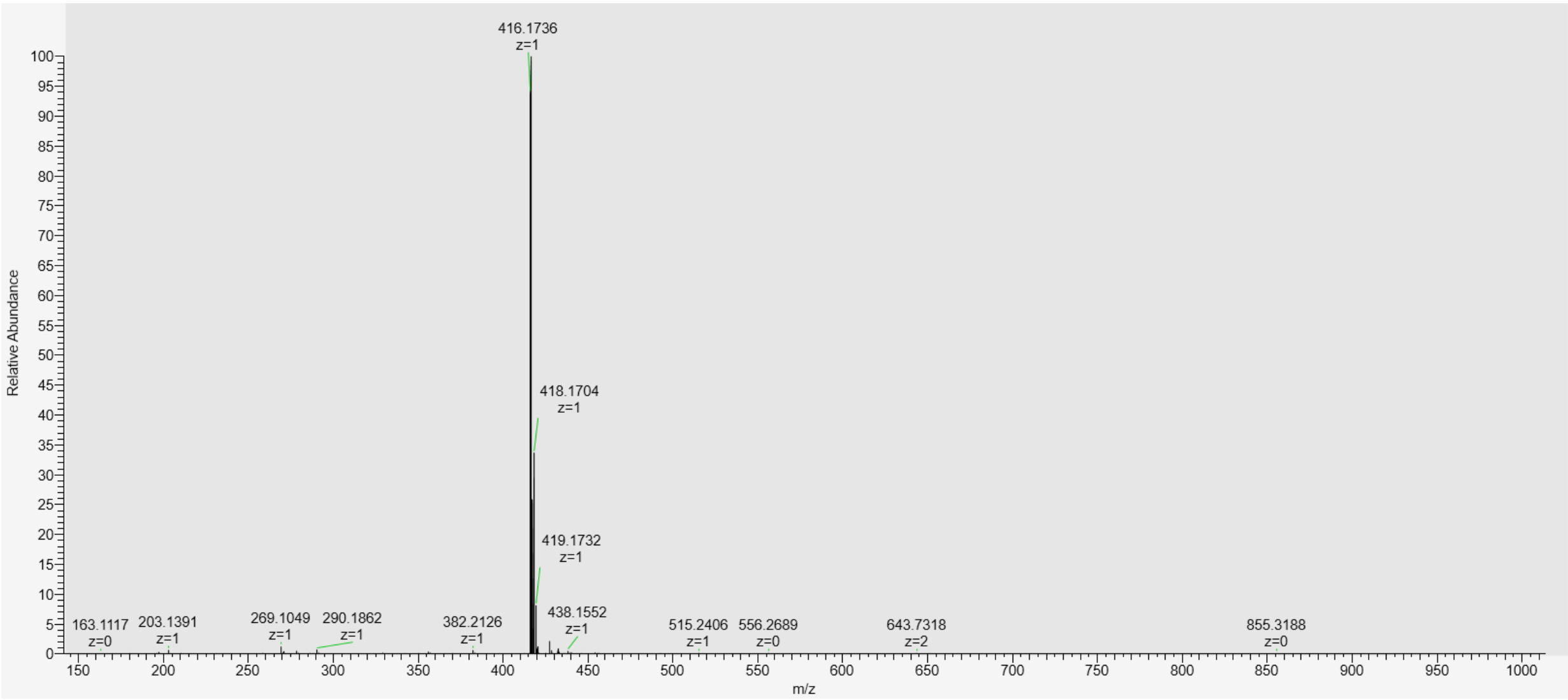

Figure S36. HRMS spectrum of compound **3**

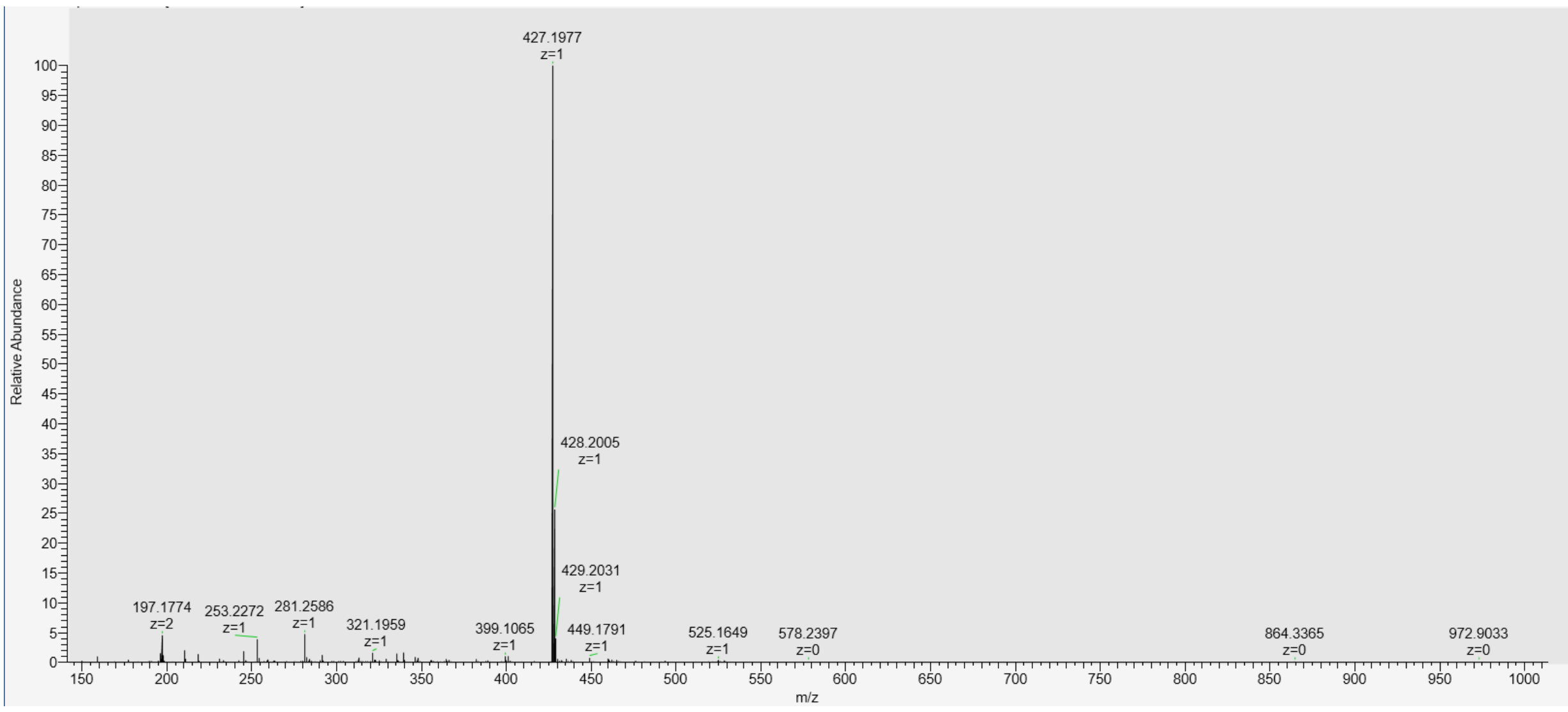

Figure S37. HRMS spectrum of compound **4**

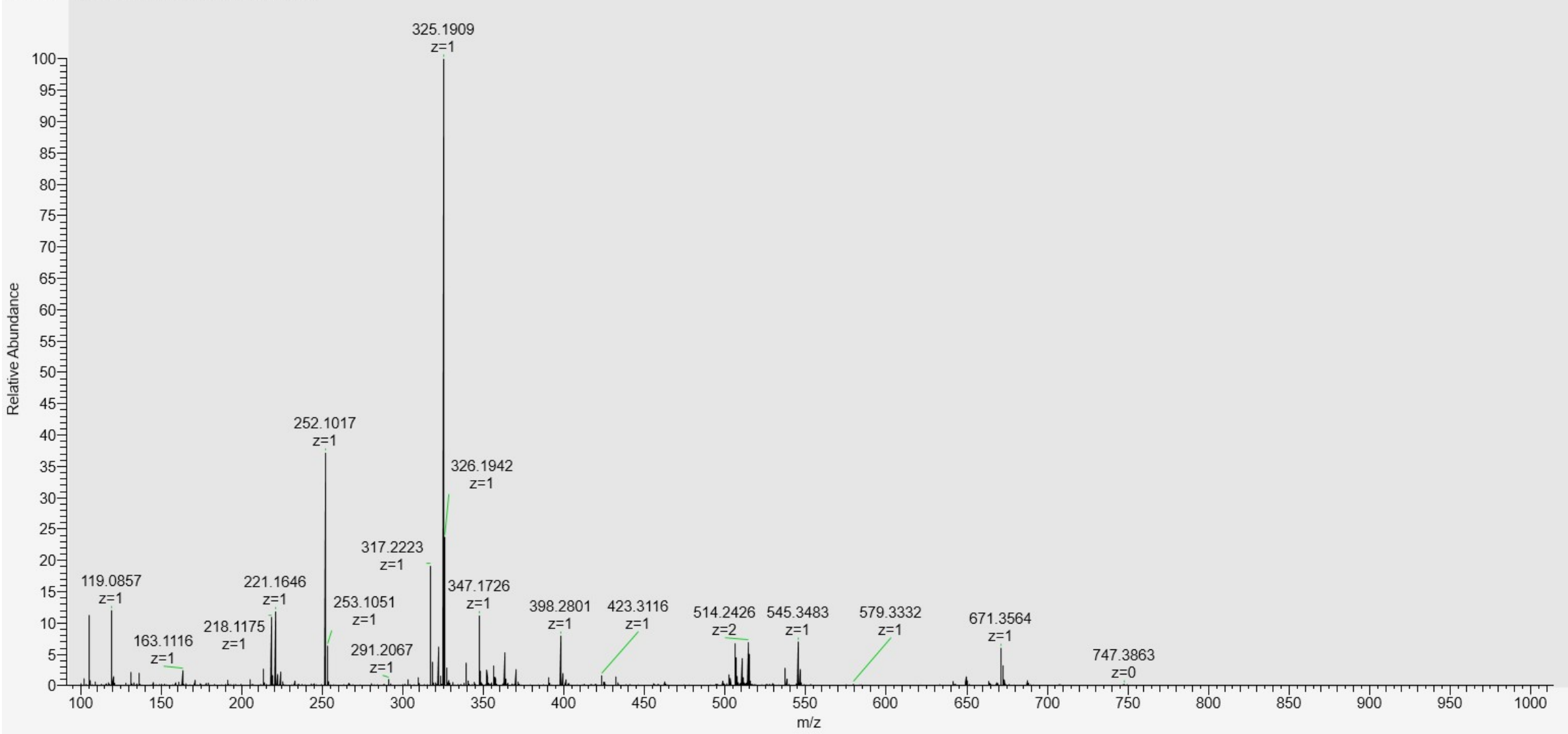

Figure S38. HRMS spectrum of compound **5**

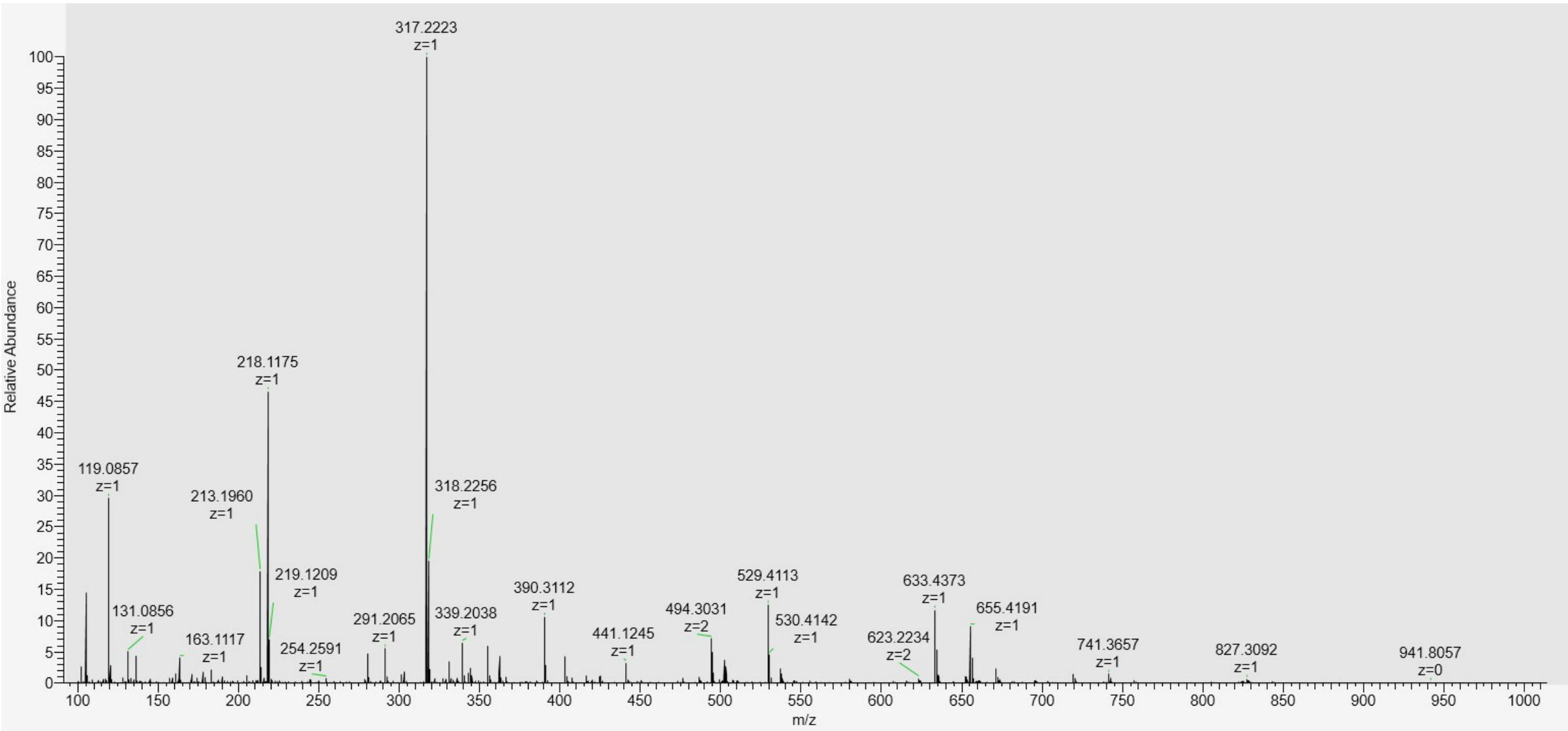

Figure S39. HRMS spectrum of compound **6**

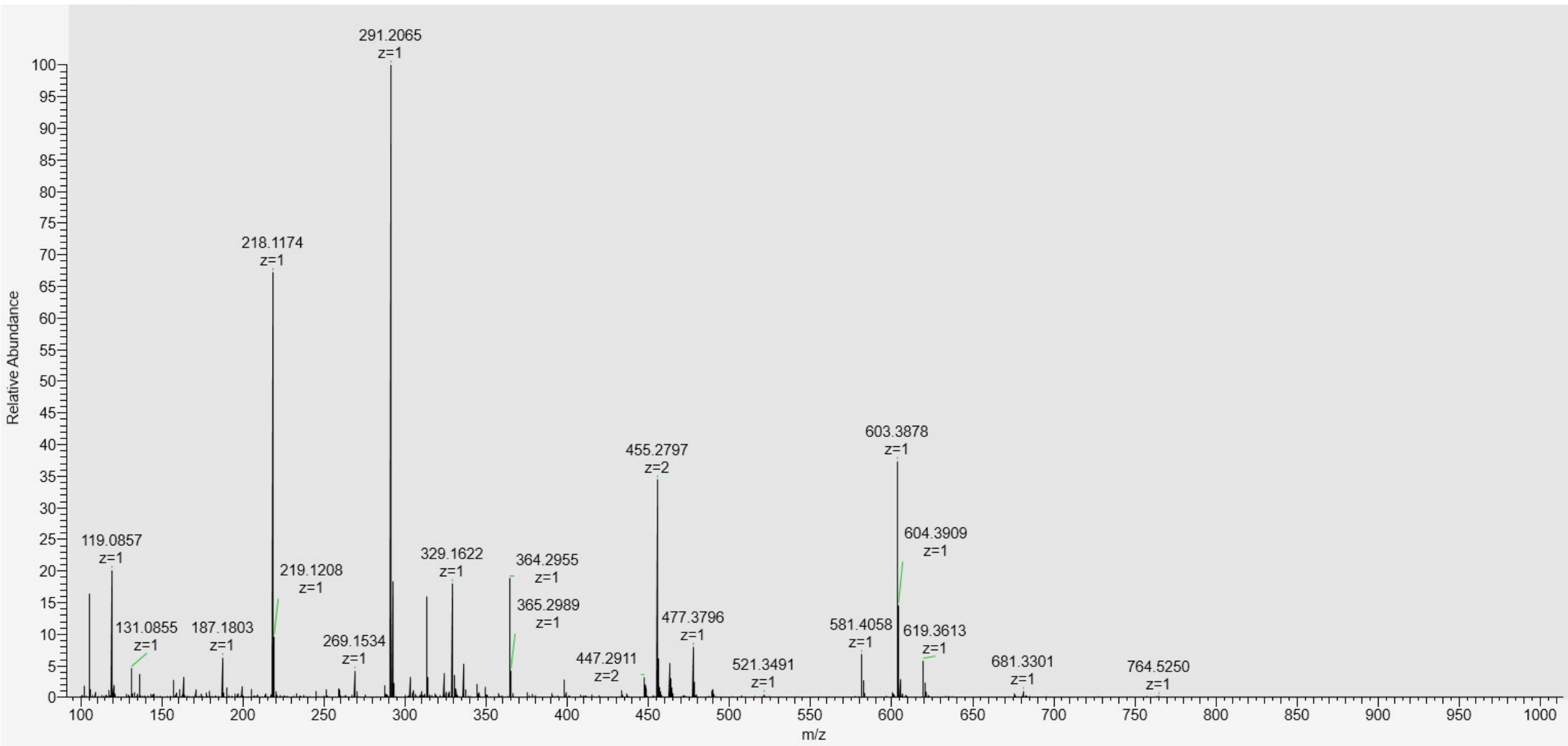

Figure S40. HRMS spectrum of compound **7**

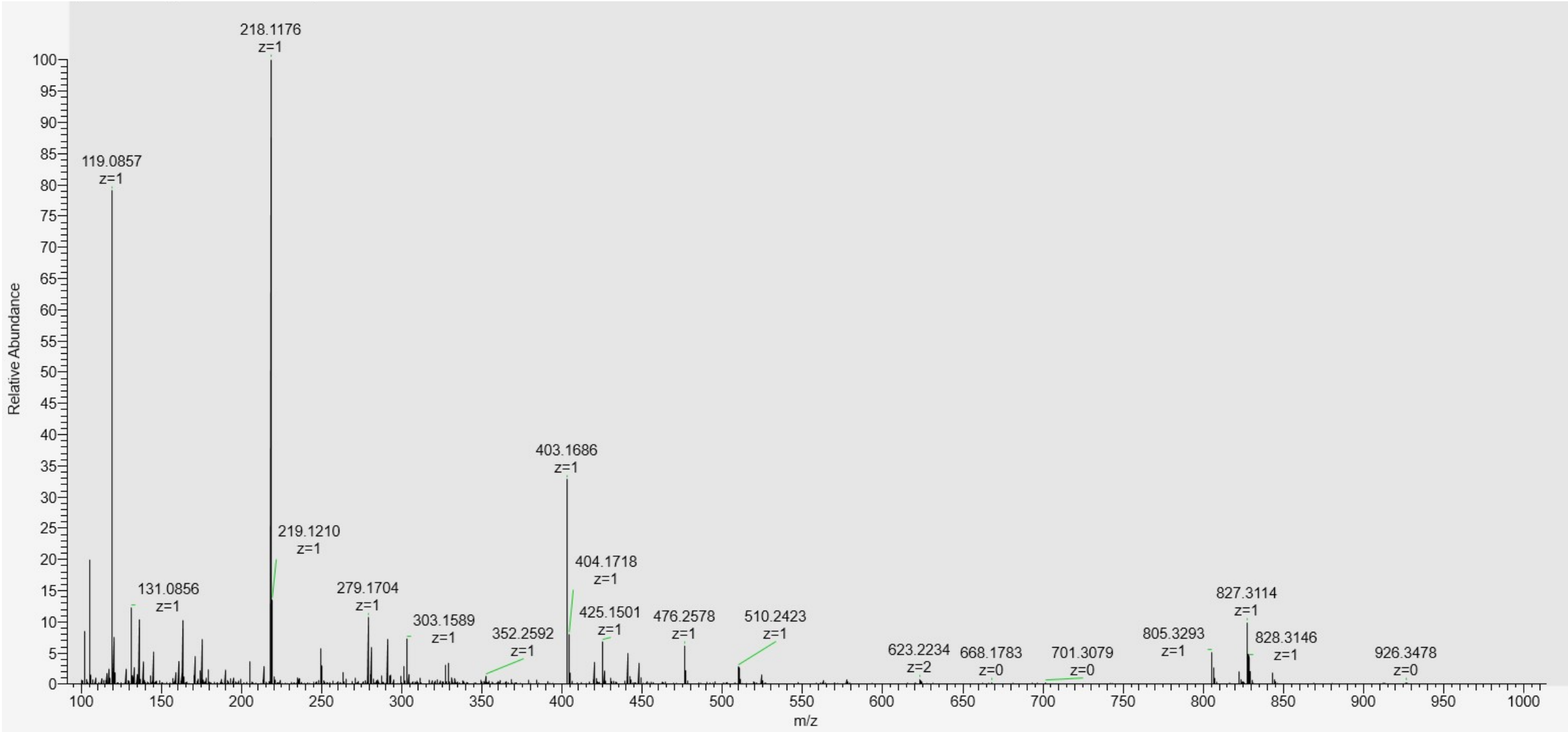

Figure S41. HRMS spectrum of compound **8**

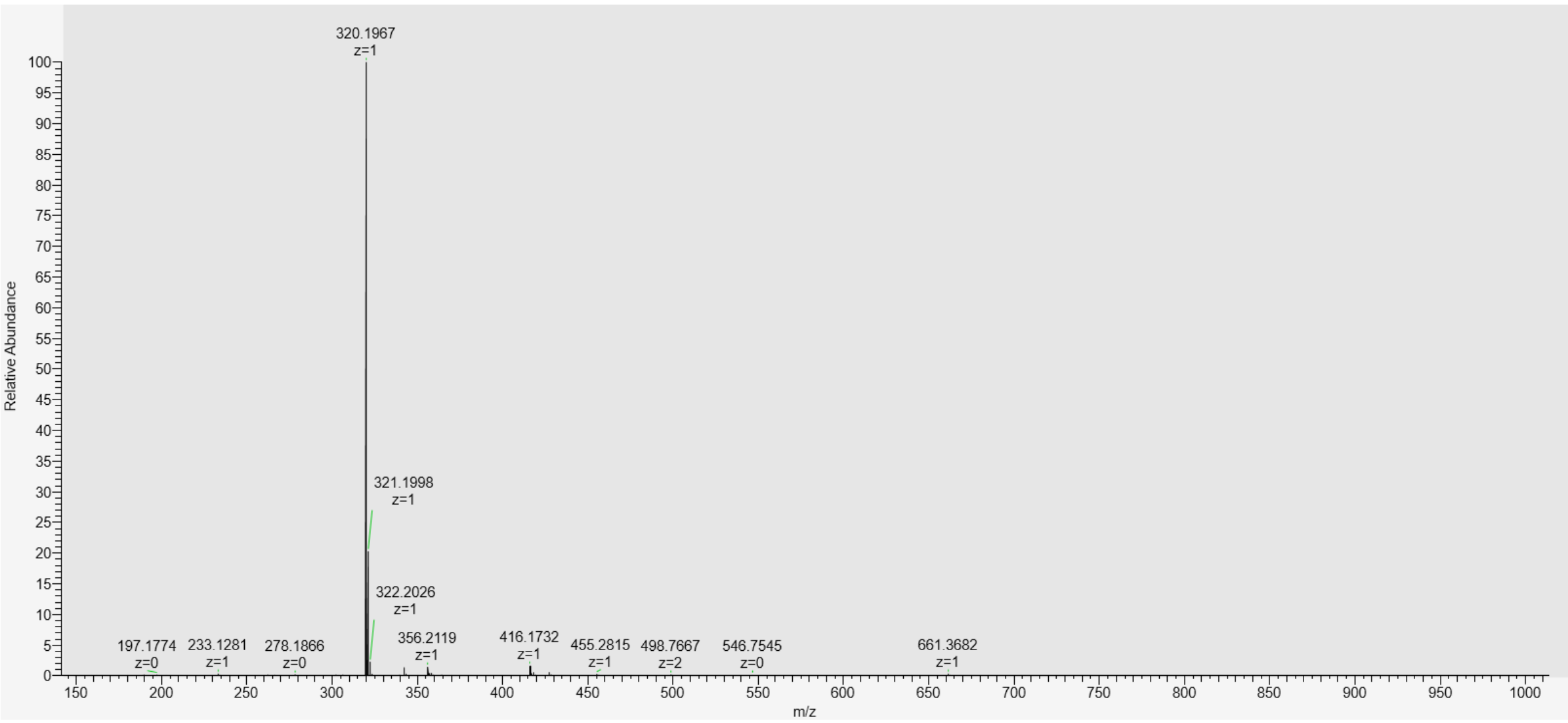

Figure S42. HRMS spectrum of compound **9**

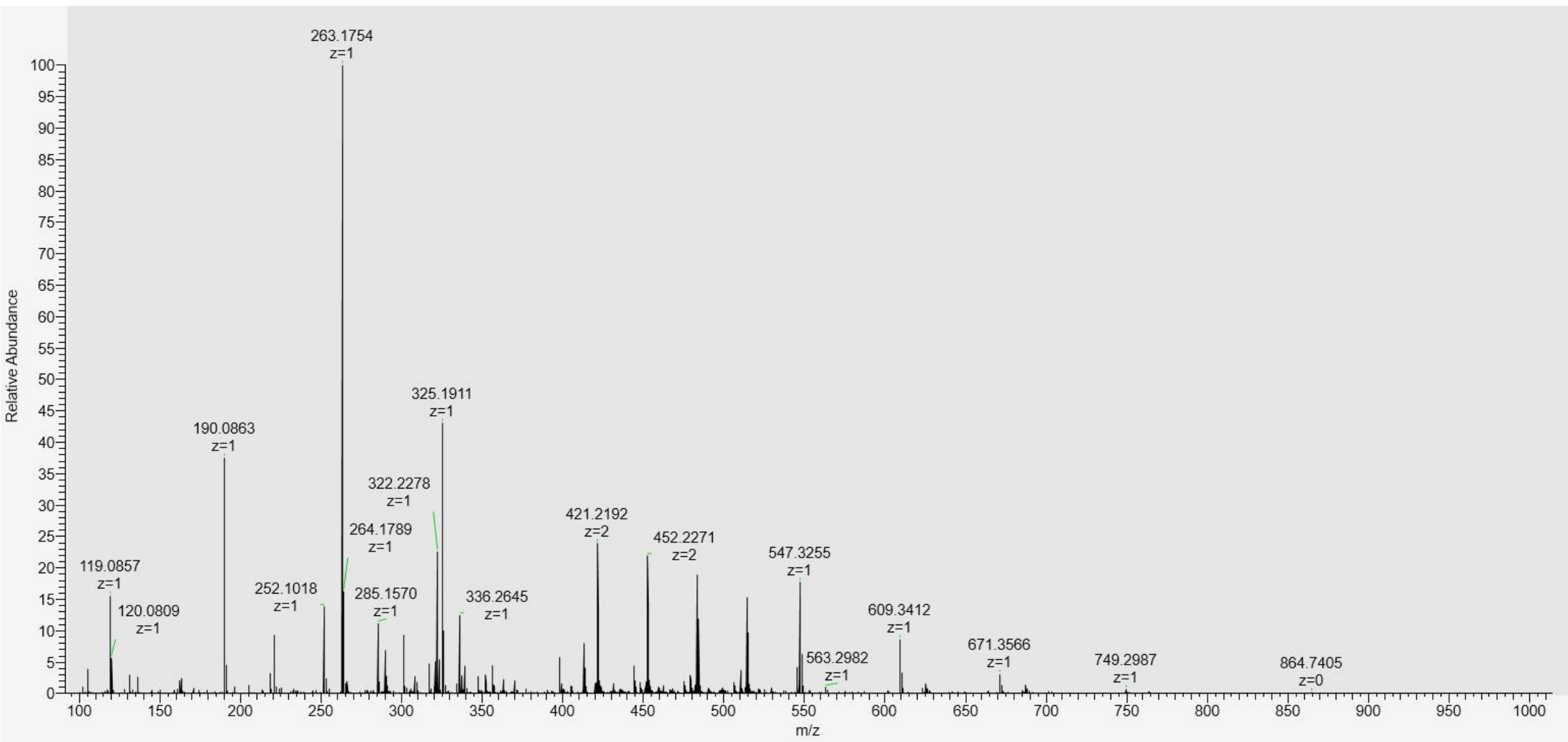

Figure S43. HRMS spectrum of compound **10**

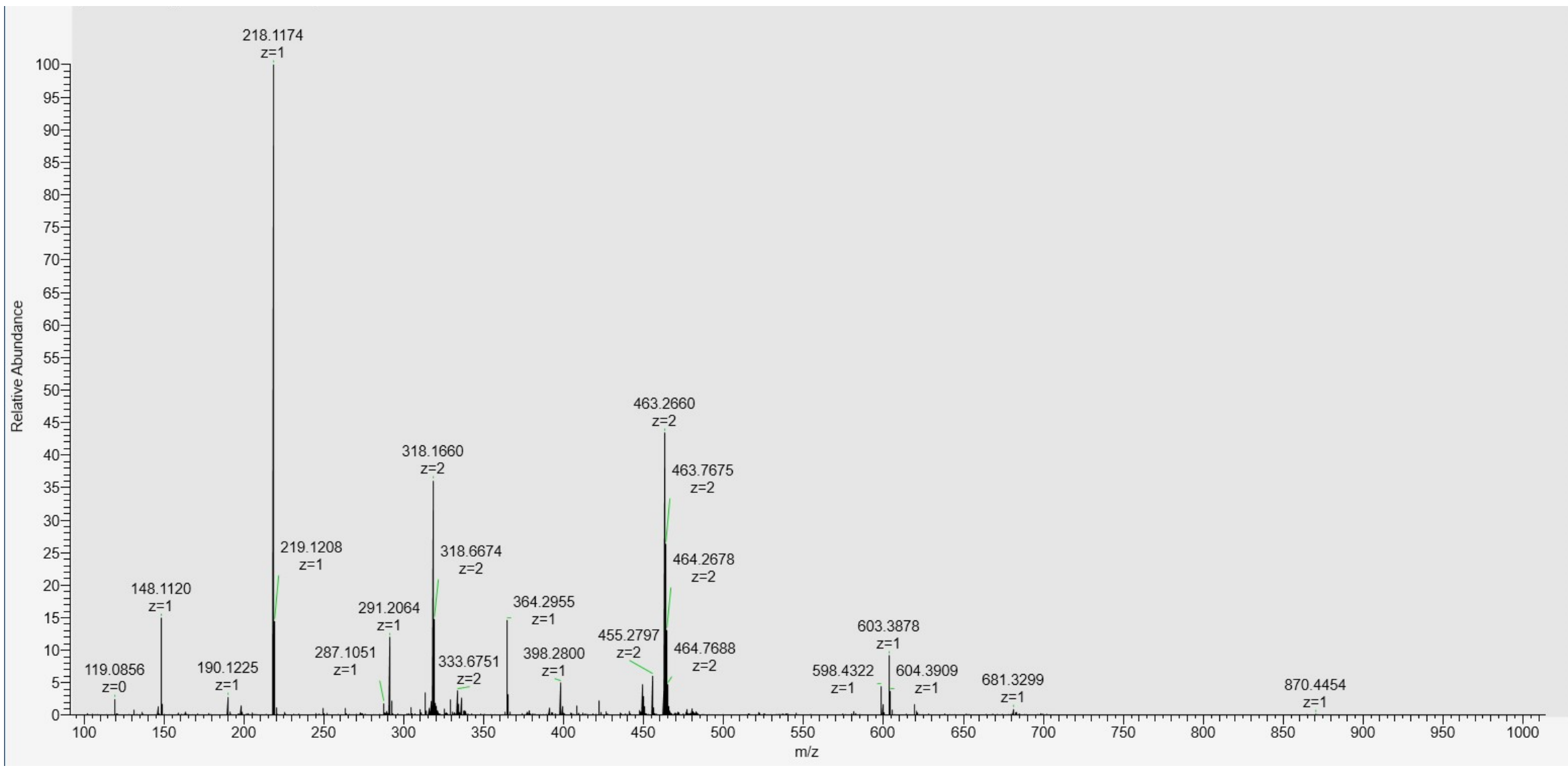

Figure S44. HRMS spectrum of compound **11**

Quantum chemical docking

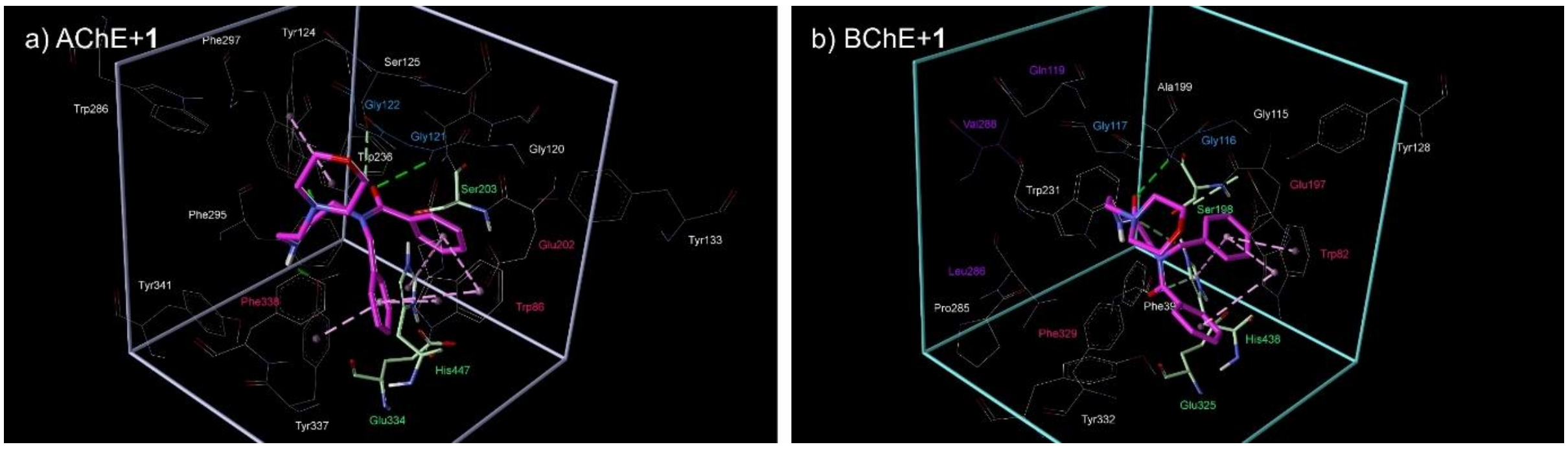

Figure S45. Placement of compound **1** inside the active site of **a)** AChE and **b)** BChE obtained by extensive quantum-chemical docking and geometry optimization at the PM7 semiempirical level of the theory. (Violet color for carbon atoms: one of the docked structures of minimal energy, non-polar hydrogen atoms are omitted for clarity)

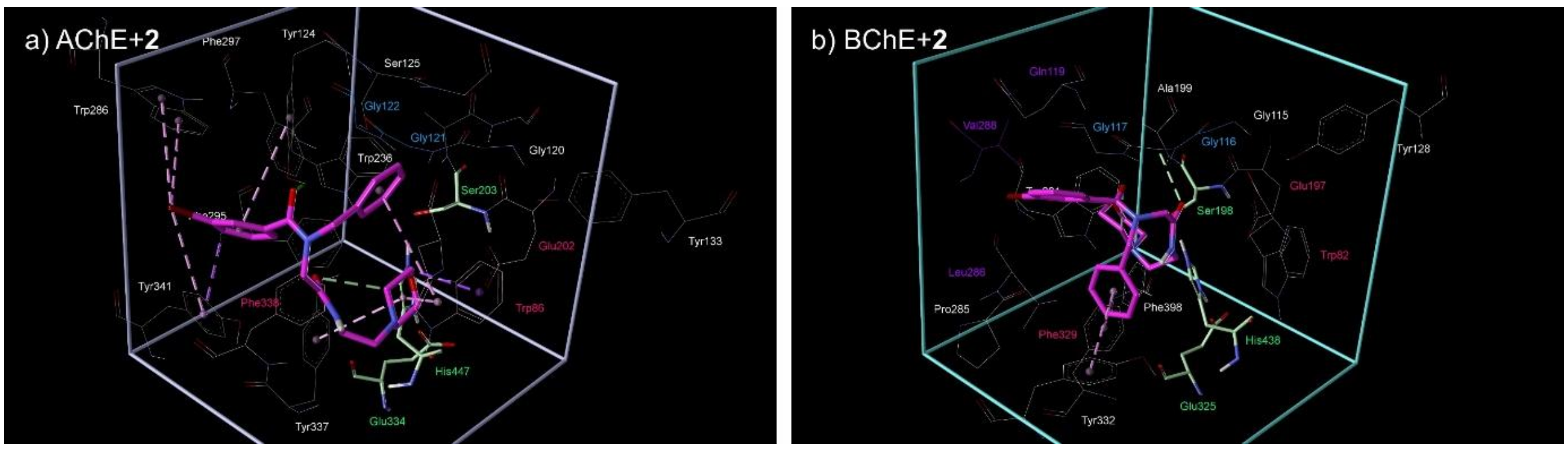

Figure S46. Placement of compound **2** inside the active site of **a)** AChE and **b)** BChE obtained by extensive quantum-chemical docking and geometry optimization at the PM7 semiempirical level of the theory. (Violet color for carbon atoms: one of the docked structures of minimal energy, non-polar hydrogen atoms are omitted for clarity)

(cc) BY

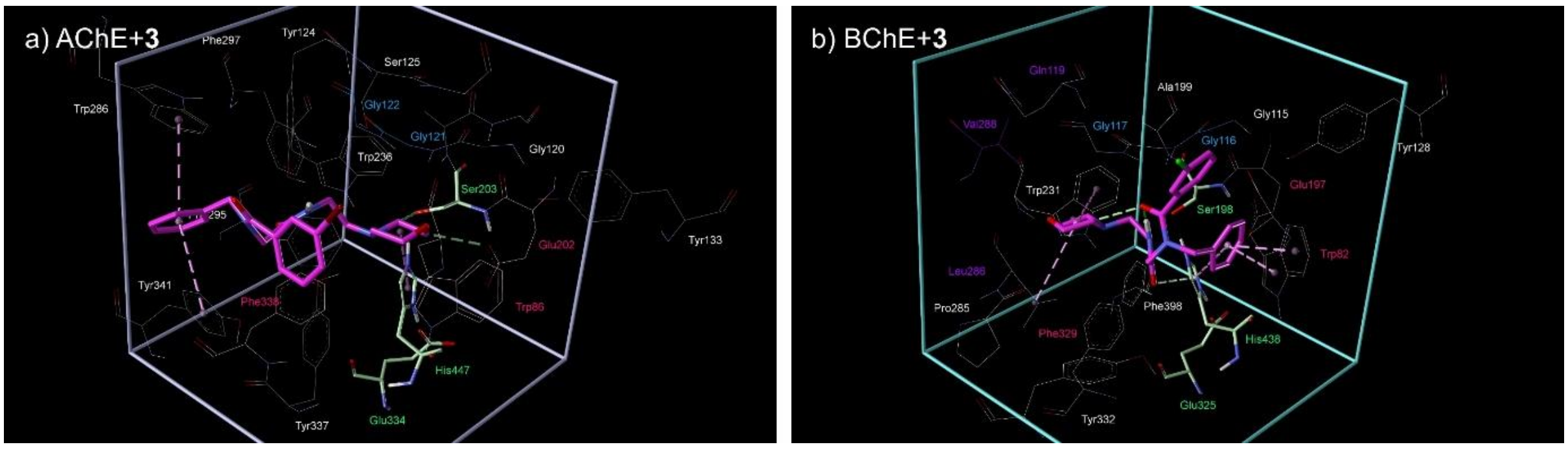

Figure S47. Placement of compound **3** inside the active site of **a)** AChE and **b)** BChE obtained by extensive quantum-chemical docking and geometry optimization at the PM7 semiempirical level of the theory. (Violet color for carbon atoms: one of the docked structures of minimal energy, non-polar hydrogen atoms are omitted for clarity)

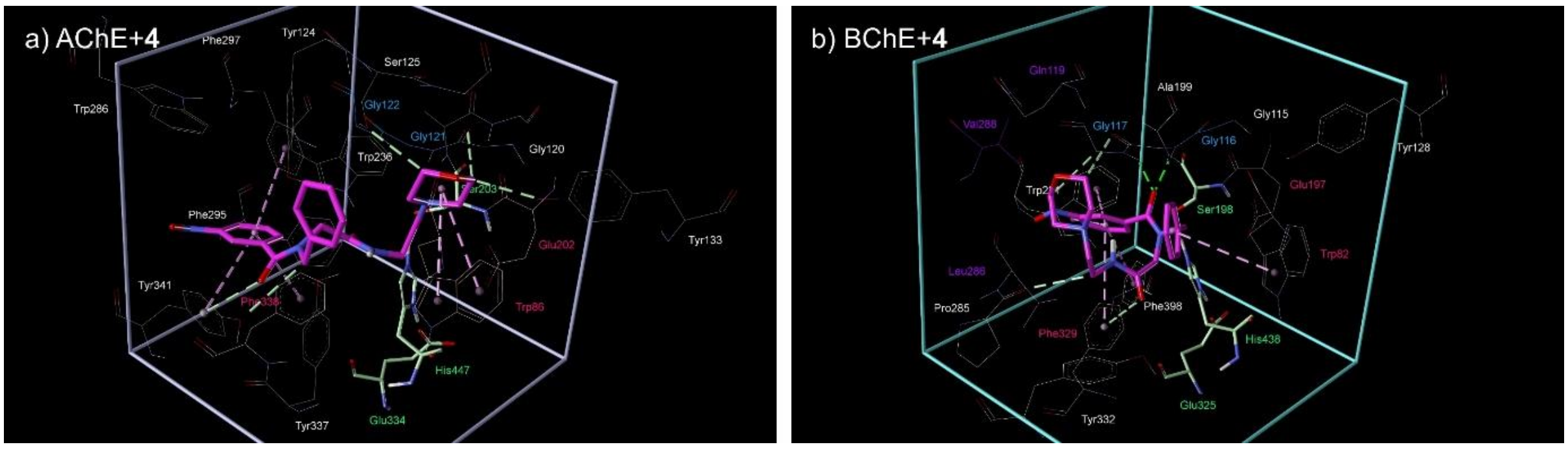

Figure S48. Placement of compound **4** inside the active site of **a)** AChE and **b)** BChE obtained by extensive quantum-chemical docking and geometry optimization at the PM7 semiempirical level of the theory. (Violet color for carbon atoms: one of the docked structures of minimal energy, non-polar hydrogen atoms are omitted for clarity)

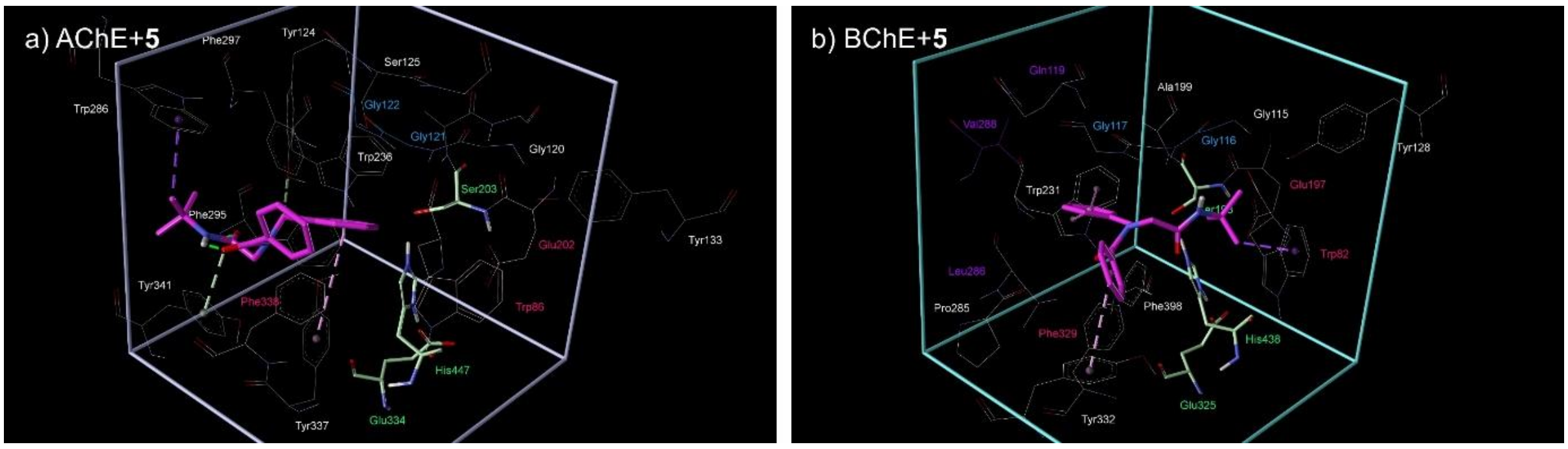

Figure S49. Placement of compound **5** inside the active site of **a)** AChE and **b)** BChE obtained by extensive quantum-chemical docking and geometry optimization at the PM7 semiempirical level of the theory. (Violet color for carbon atoms: one of the docked structures of minimal energy, non-polar hydrogen atoms are omitted for clarity)

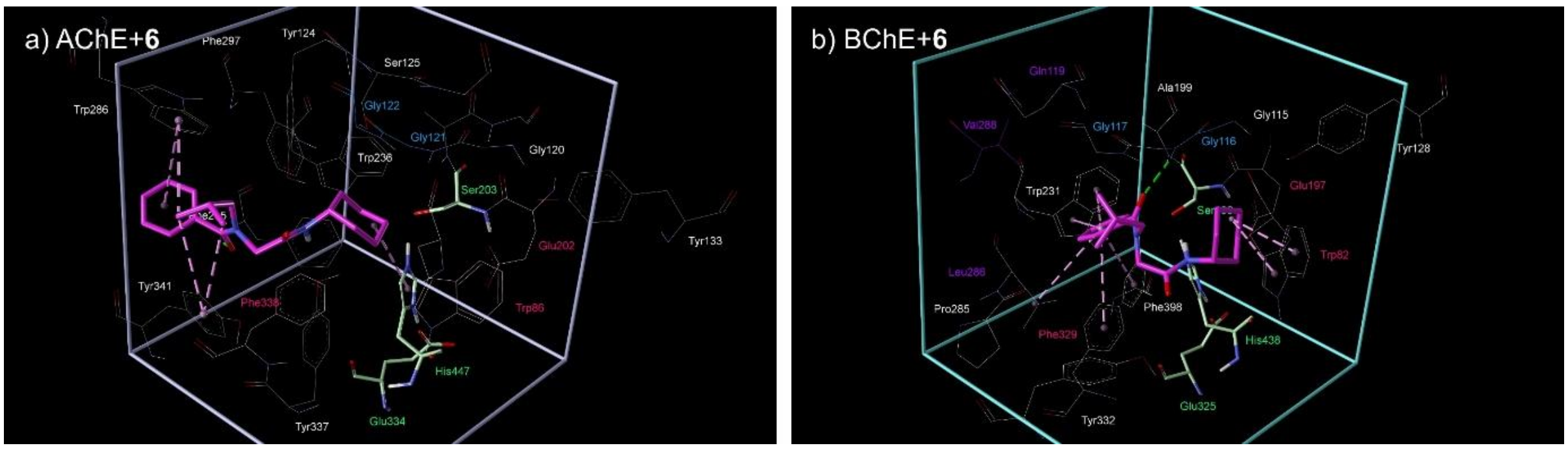

Figure S50. Placement of compound **6** inside the active site of **a)** AChE and **b)** BChE obtained by extensive quantum-chemical docking and geometry optimization at the PM7 semiempirical level of the theory. (Violet color for carbon atoms: one of the docked structures of minimal energy, non-polar hydrogen atoms are omitted for clarity).

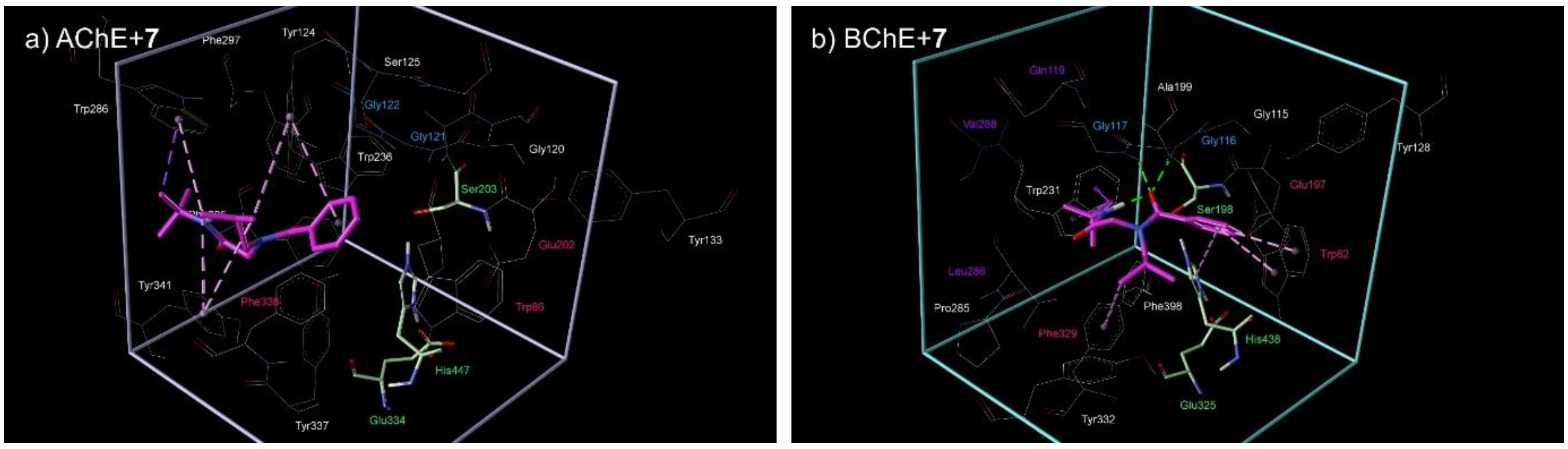

Figure S51. Placement of compound **7** inside the active site of **a)** AChE and **b)** BChE obtained by extensive quantum-chemical docking and geometry optimization at the PM7 semiempirical level of the theory. (Violet color for carbon atoms: one of the docked structures of minimal energy, non-polar hydrogen atoms are omitted for clarity)

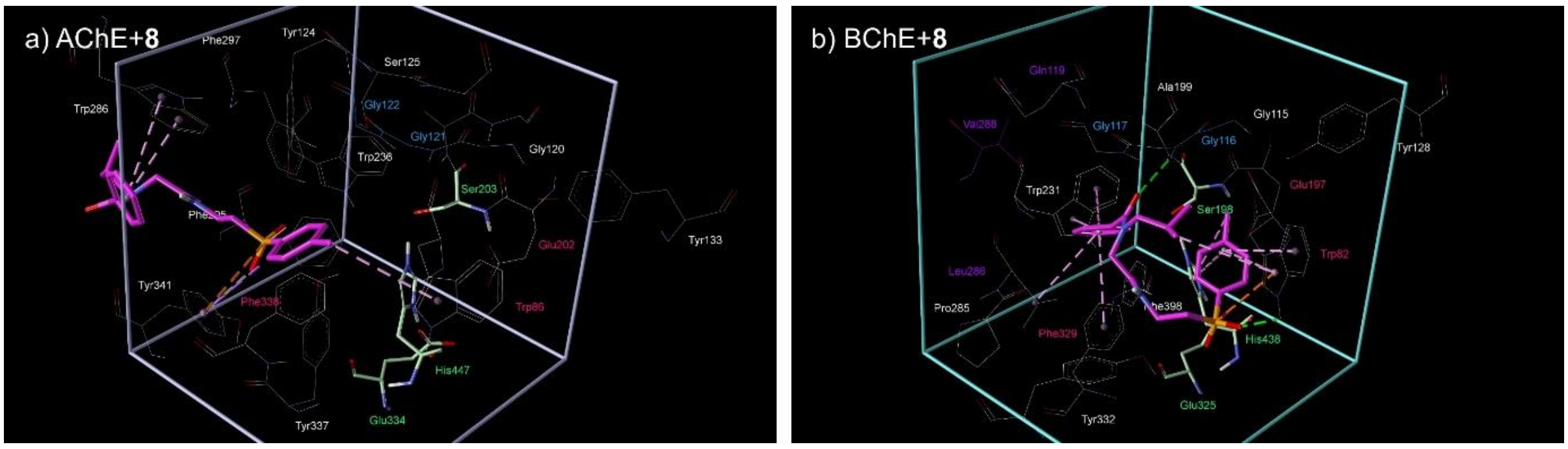

Figure S52. Placement of compound **8** inside the active site of **a)** AChE and **b)** BChE obtained by extensive quantum-chemical docking and geometry optimization at the PM7 semiempirical level of the theory. (Violet color for carbon atoms: one of the docked structures of minimal energy, non-polar hydrogen atoms are omitted for clarity)

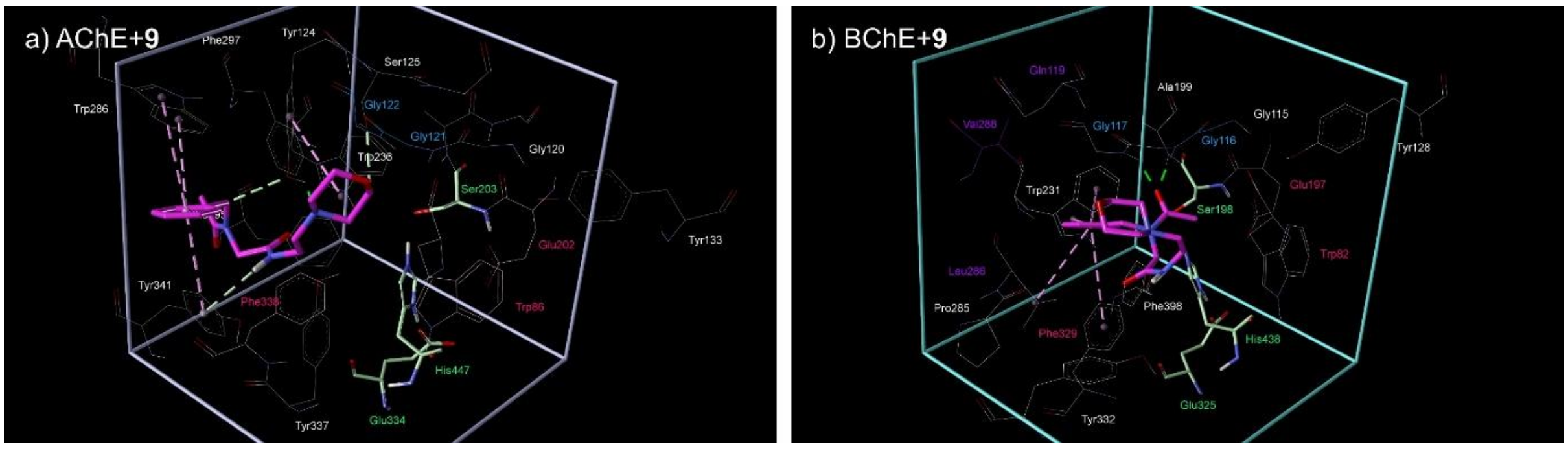

Figure S53. Placement of compound **9** inside the active site of **a)** AChE and **b)** BChE obtained by extensive quantum-chemical docking and geometry optimization at the PM7 semiempirical level of the theory. (Violet color for carbon atoms: one of the docked structures of minimal energy, non-polar hydrogen atoms are omitted for clarity)

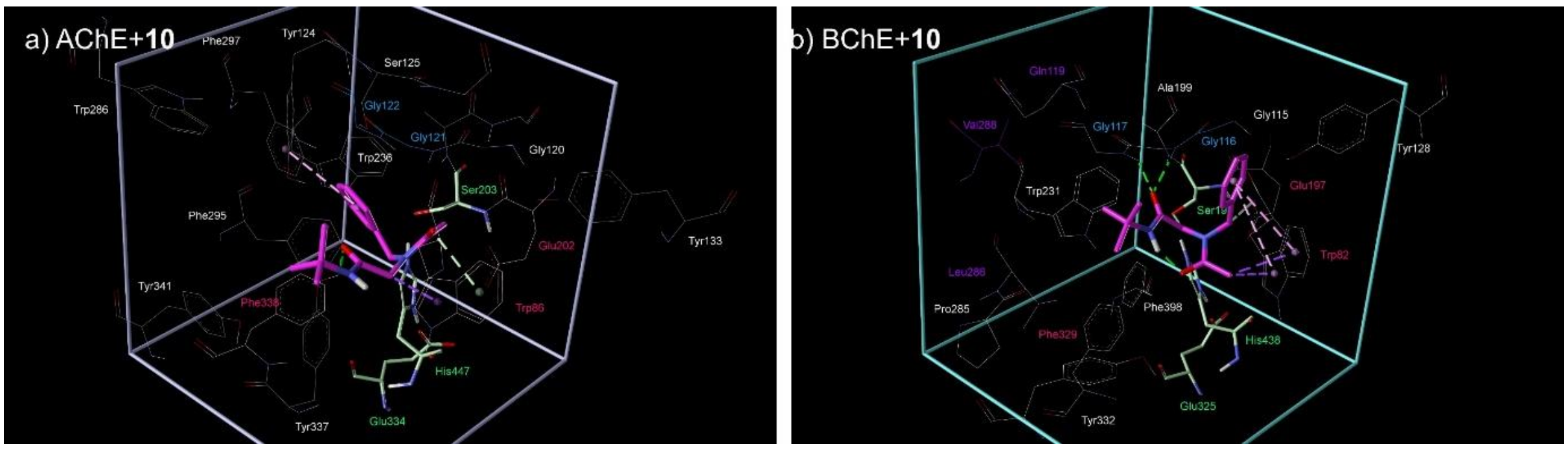

Figure S54. Placement of compound **10** inside the active site of **a)** AChE and **b)** BChE obtained by extensive quantum-chemical docking and geometry optimization at the PM7 semiempirical level of the theory. (Violet color for carbon atoms: one of the docked structures of minimal energy, non-polar hydrogen atoms are omitted for clarity)

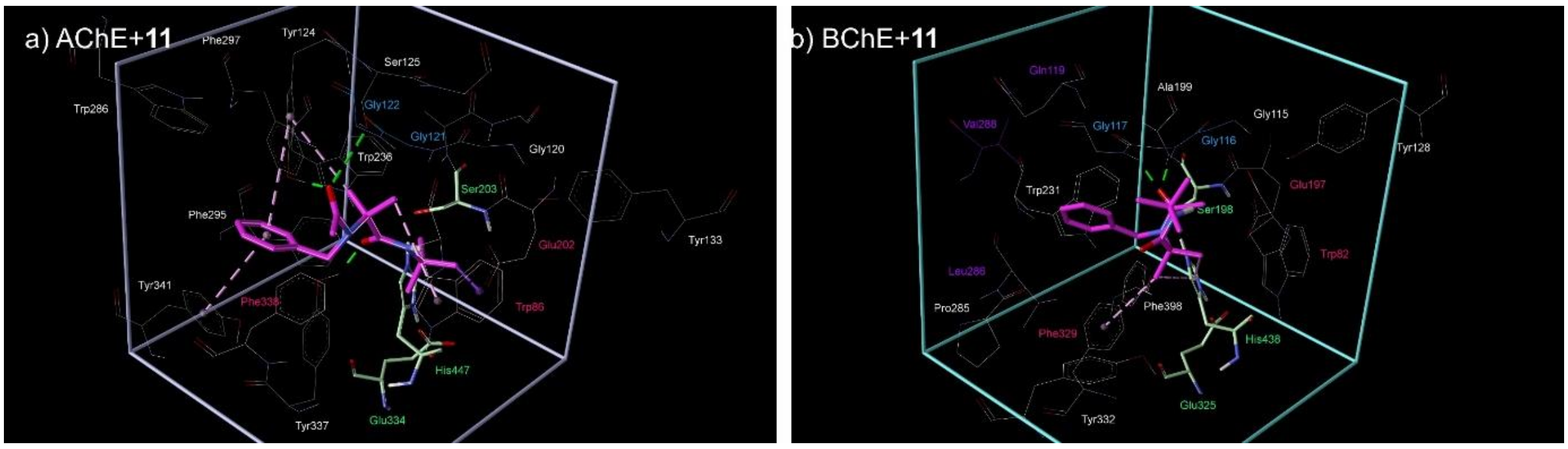

Figure S55. Placement of compound **11** inside the active site of **a)** AChE and **b)** BChE obtained by extensive quantum-chemical docking and geometry optimization at the PM7 semiempirical level of the theory. (Violet color for carbon atoms: one of the docked structures of minimal energy, non-polar hydrogen atoms are omitted for clarity)

Molecular charge distribution

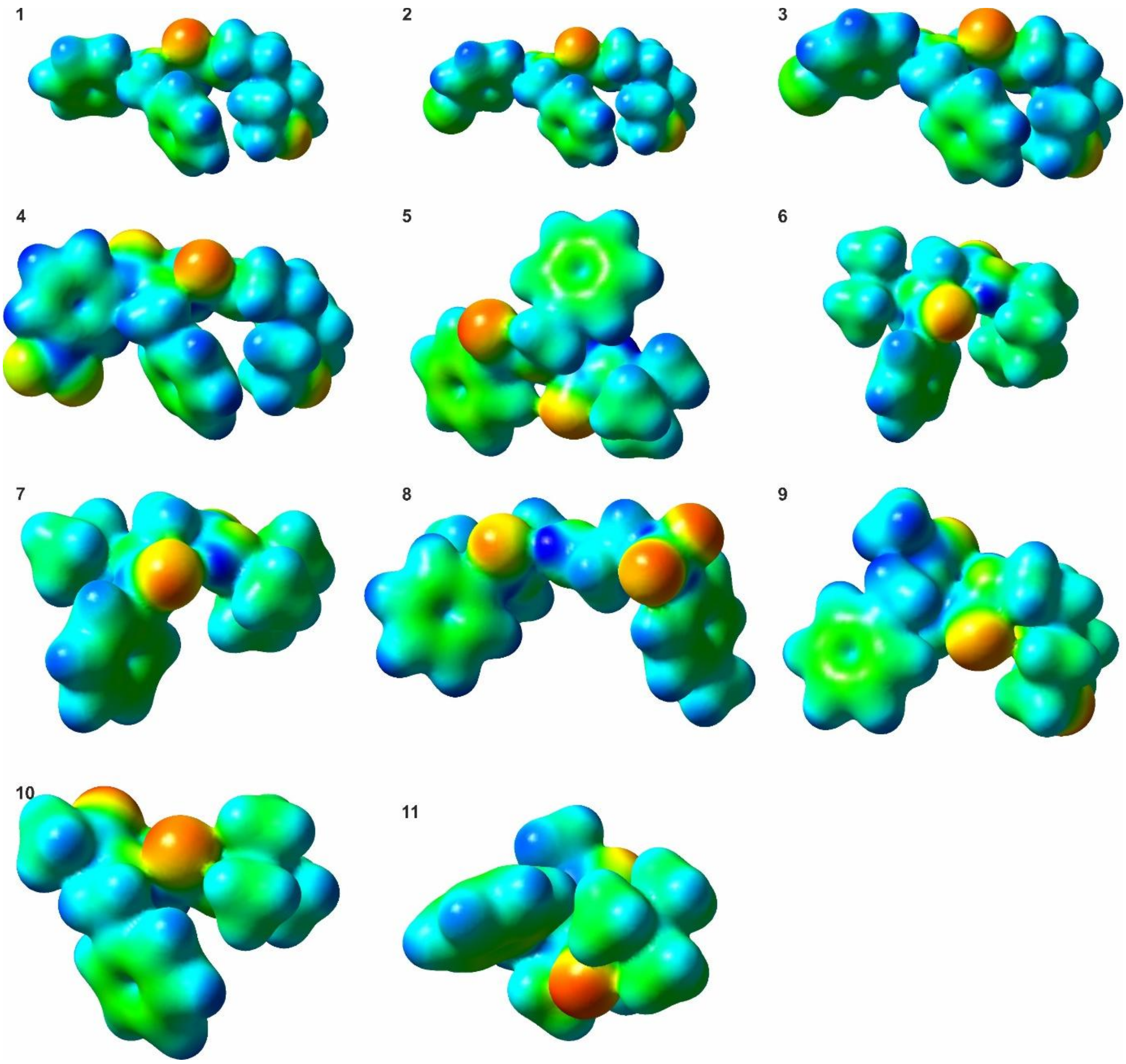

Figure S56. Molecular charge distribution for all compounds visualized by electrostatic potential mapped on the total electronic density calculated at B3LYP-D3BJ/6-31G(d) level of the theory (isovalue for density: 0.01)

Calculated physico-chemical properties

Table S1. Calculated physiochemical properties (molecular weight, MW; lipophilicity coefficient, logP; number of hydrogen bond donors, HBD, and acceptors, HBA; rotable bonds, RB; polar surface area, PSA) of compounds

Compound	MW/100	log*P*	HBD	HBA	RB	PSA/10	LLE hBChE	LLE hAChE	LE hBChE	LE hAChE
**1**	3.815	1.691	1	4	8	6.188	3.56	0.26	0.26	0.19
**2**	4.604	2.459	1	4	8	6.188	1.77	1.91	0.20	0.18
**3**	4.159	2.295	1	4	8	6.188	2.38	1.35	0.23	0.18
**4**	4.265	1.631	1	6	9	10.502	2.68	1.03	0.19	0.17
**5**	3.244	2.944	1	2	6	7.774	2.78	0.48	0.33	0.19
**6**	3.164	3.21	1	2	6	4.941	0.94	2.42	0.25	0.20
**7**	2.904	2.464	1	2	6	4.941	1.80	1.68	0.28	0.23
**8**	4.025	2.675	1	4	8	8.355	3.00	0.47	0.28	0.17
**9**	3.194	0.164	1	4	7	6.188	4.16	-0.48	0.26	0.22
**10**	2.624	1.09	1	2	5	4.941	3.61	0.09	0.35	0.27
**11**	2.904	2.092	1	2	5	4.941	2.20	1.66	0.29	0.26
**Recomm.**	4.5	5	3	7	8	7	≥5	≥5	≥0.3	≥0.3

(cc) BY
