# Supplementary material for: Cholinesterases inhibition profiles with Ugi-derived peptidemimetics: A combined experimental and computational study
Source: ADMET DMPK. 2026 Jun 23;14:3292. doi: 10.5599/admet.3292 (PMC13499666; doi:10.5599/admet.3292)
Supplement: Supplementary file 2 [file ADMET-14-3292-S1.docx]

ADMET & DMPK **14** (2026) S3292

**Open Access : ISSN : 1848-7718**
[***http://www.pub.iapchem.org/ojs/index.php/admet***](http://www.pub.iapchem.org/ojs/index.php/admet)

Supplementary material to

Cholinesterases inhibition profiles with Ugi-derived peptide­mimetics: A combined experimental and computational study

Alma Ramić^1^^<https://orcid.org/0000-0001-6003-7241>^, Toni Divjak^1^^<https://orcid.org/0009-0001-2981-066X>^, Lucija Hadrović^1^^<https://orcid.org/0009-0004-3740-1083>^, Matej Pavlinić^1^^<https://orcid.org/0009-0002-4872-1774>^, Ana Matošević^2^^<https://orcid.org/0009-0002-4459-7622>^, Anita Bosak^2^^<https://orcid.org/0000-0003-0164-4994>^, Jakov Borovec^1^^<https://orcid.org/0009-0005-2931-0381>^, Bruna Bakota^1^^<https://orcid.org/0009-0005-5309-2505>^, Tomica Hrenar^1^^<https://orcid.org/0000-0002-4570-0524>^ and
Ines Primožič1^1^^<https://orcid.org/0000-0003-1154-4735>^

^1^Department of Chemistry, Faculty of Science, University of Zagreb, Horvatovac 102a, Zagreb, Croatia
^2^Institute for Medical Research and Occupational Health, Ksaverska cesta 2, Zagreb, Croatia

ADMET & DMPK **14** (2026) 3292; <https://doi.org/10.5599/admet.3292>

NMR spectra

**Figure S1.** ^1^H NMR (400 MHz, CD_3_OH) of compound **1**

**Figure S2.** ^13^C NMR (100 MHz, CD_3_OH) of compound **1**

**Figure S3.** ^1^H NMR (400 MHz, CD_3_OH) of compound **2**

**Figure S4.** ^13^C NMR (100 MHz, CD_3_OH) of compound **2**

**Figure S5.** ^1^H NMR (400 MHz, CD_3_OH) of compound **3**

**Figure S6.** ^13^C NMR (100 MHz, CD_3_OH) of compound **3**

**Figure S7.** ^1^H NMR (400 MHz, CD_3_OH) of compound **4**

**Figure S8.** ^13^C NMR (100 MHz, CD_3_OH) of compound **4**

**Figure S9.** ^1^H NMR (400 MHz, CDCl_3_) of compound **5**

**Figure S10.** ^13^C NMR (100 MHz, CDCl_3_) of compound **5**

**Figure S11.** ^1^H NMR (400 MHz, CDCl_3_) of compound **6**

**Figure S12.** ^13^C NMR (100 MHz, CDCl_3_) of compound **6**

**Figure S13.** ^1^H NMR (400 MHz, CDCl_3_) of compound **7**

**Figure S14.** ^13^C NMR (100 MHz, CDCl_3_) of compound **7**

**Figure S15.** ^1^H NMR (400 MHz, CDCl_3_) of compound **8**

**Figure S16.** ^13^C NMR (100 MHz, CDCl_3_) of compound **8**

**Figure S17.** ^1^H NMR (400 MHz, CD_3_OH) of compound **9**

**Figure S18.** ^13^C NMR (100 MHz, CD_3_OH) of compound **9**

**Figure S19.** ^1^H NMR (400 MHz, CDCl_3_) of compound **10**

**Figure S20.** ^13^C NMR (100 MHz, CDCl_3_) of compound **10**

**Figure S21.** ^1^H NMR (400 MHz, CDCl_3_) of compound **11**

**Figure S22.** ^13^C NMR (100 MHz, CDCl_3_) of compound **11**

HPLC chromatograms

**Figure S23**. HPLC chromatogram of compound **1**

**Figure S24**. HPLC chromatogram of compound **2**

**Figure S25**. HPLC chromatogram of compound **3**

**Figure S26**. HPLC chromatogram of compound **4**

**Figure S27**. HPLC chromatogram of compound **5**

**Figure S28**. HPLC chromatogram of compound **6**

**Figure S29**. HPLC chromatogram of compound **7**

**Figure S30**. HPLC chromatogram of compound **8**

**Figure S31**. HPLC chromatogram of compound **9**

**Figure S32**. HPLC chromatogram of compound **10**

**Figure S33**. HPLC chromatogram of compound **11**

3.HRMS spectra

**Figure S34**. HRMS spectrum of compound **1**

**Figure S35**. HRMS spectrum of compound **2**

**Figure S36**. HRMS spectrum of compound **3**

**Figure S37**. HRMS spectrum of compound **4**

**Figure S38**. HRMS spectrum of compound **5**

**Figure S39**. HRMS spectrum of compound **6**

**Figure S40**. HRMS spectrum of compound **7**

**Figure S41**. HRMS spectrum of compound **8**

**Figure S42**. HRMS spectrum of compound **9**

**Figure S43**. HRMS spectrum of compound **10**

**Figure S44**. HRMS spectrum of compound **11**

Quantum chemical docking

**Figure S45.** Placement of compound **1** inside the active site of **a)** AChE and **b)** BChE obtained by extensive quantum-chemical docking and geometry optimization at the PM7 semiempirical level of the theory. (Violet color for carbon atoms: one of the docked structures of minimal energy, non-polar hydrogen atoms are omitted for clarity)

**Figure S46.** Placement of compound **2** inside the active site of **a)** AChE and **b)** BChE obtained by extensive quantum-chemical docking and geometry optimization at the PM7 semiempirical level of the theory. (Violet color for carbon atoms: one of the docked structures of minimal energy, non-polar hydrogen atoms are omitted for clarity)

**Figure S47.** Placement of compound **3** inside the active site of **a)** AChE and **b)** BChE obtained by extensive quantum-chemical docking and geometry optimization at the PM7 semiempirical level of the theory. (Violet color for carbon atoms: one of the docked structures of minimal energy, non-polar hydrogen atoms are omitted for clarity)

**Figure S48.** Placement of compound **4** inside the active site of **a)** AChE and **b)** BChE obtained by extensive quantum-chemical docking and geometry optimization at the PM7 semiempirical level of the theory. (Violet color for carbon atoms: one of the docked structures of minimal energy, non-polar hydrogen atoms are omitted for clarity)

**Figure S49.** Placement of compound **5** inside the active site of **a)** AChE and **b)** BChE obtained by extensive quantum-chemical docking and geometry optimization at the PM7 semiempirical level of the theory. (Violet color for carbon atoms: one of the docked structures of minimal energy, non-polar hydrogen atoms are omitted for clarity)

**Figure S50.** Placement of compound **6** inside the active site of **a)** AChE and **b)** BChE obtained by extensive quantum-chemical docking and geometry optimization at the PM7 semiempirical level of the theory. (Violet color for carbon atoms: one of the docked structures of minimal energy, non-polar hydrogen atoms are omitted for clarity).

**Figure S51.** Placement of compound **7** inside the active site of **a)** AChE and **b)** BChE obtained by extensive quantum-chemical docking and geometry optimization at the PM7 semiempirical level of the theory. (Violet color for carbon atoms: one of the docked structures of minimal energy, non-polar hydrogen atoms are omitted for clarity)

**Figure S52.** Placement of compound **8** inside the active site of **a)** AChE and **b)** BChE obtained by extensive quantum-chemical docking and geometry optimization at the PM7 semiempirical level of the theory. (Violet color for carbon atoms: one of the docked structures of minimal energy, non-polar hydrogen atoms are omitted for clarity)

**Figure S53.** Placement of compound **9** inside the active site of **a)** AChE and **b)** BChE obtained by extensive quantum-chemical docking and geometry optimization at the PM7 semiempirical level of the theory. (Violet color for carbon atoms: one of the docked structures of minimal energy, non-polar hydrogen atoms are omitted for clarity)

**Figure S54.** Placement of compound **10** inside the active site of **a)** AChE and **b)** BChE obtained by extensive quantum-chemical docking and geometry optimization at the PM7 semiempirical level of the theory. (Violet color for carbon atoms: one of the docked structures of minimal energy, non-polar hydrogen atoms are omitted for clarity)

**Figure S55.** Placement of compound **11** inside the active site of **a)** AChE and **b)** BChE obtained by extensive quantum-chemical docking and geometry optimization at the PM7 semiempirical level of the theory. (Violet color for carbon atoms: one of the docked structures of minimal energy, non-polar hydrogen atoms are omitted for clarity)

Molecular charge distribution

**Figure S56.** Molecular charge distribution for all compounds visualized by electrostatic potential mapped on the total electronic density calculated at B3LYP-D3BJ/6-31G(d) level of the theory (isovalue for density: 0.01)

Calculated physico-chemical properties

**Table S1.** Calculated physiochemical properties (molecular weight, MW; lipophilicity coefficient, logP; number of hydrogen bond donors, HBD, and acceptors, HBA; rotable bonds, RB; polar surface area, PSA) of compounds

| Compound | MW/100 | log*P* | HBD | HBA | RB | PSA/10 | LLE hBChE | LLE hAChE | LE hBChE | LE hAChE |
| --- | --- | --- | --- | --- | --- | --- | --- | --- | --- | --- |
| **1** | 3.815 | 1.691 | 1 | 4 | 8 | 6.188 | 3.56 | 0.26 | 0.26 | 0.19 |
| **2** | 4.604 | 2.459 | 1 | 4 | 8 | 6.188 | 1.77 | 1.91 | 0.20 | 0.18 |
| **3** | 4.159 | 2.295 | 1 | 4 | 8 | 6.188 | 2.38 | 1.35 | 0.23 | 0.18 |
| **4** | 4.265 | 1.631 | 1 | 6 | 9 | 10.502 | 2.68 | 1.03 | 0.19 | 0.17 |
| **5** | 3.244 | 2.944 | 1 | 2 | 6 | 7.774 | 2.78 | 0.48 | 0.33 | 0.19 |
| **6** | 3.164 | 3.21 | 1 | 2 | 6 | 4.941 | 0.94 | 2.42 | 0.25 | 0.20 |
| **7** | 2.904 | 2.464 | 1 | 2 | 6 | 4.941 | 1.80 | 1.68 | 0.28 | 0.23 |
| **8** | 4.025 | 2.675 | 1 | 4 | 8 | 8.355 | 3.00 | 0.47 | 0.28 | 0.17 |
| **9** | 3.194 | 0.164 | 1 | 4 | 7 | 6.188 | 4.16 | -0.48 | 0.26 | 0.22 |
| **10** | 2.624 | 1.09 | 1 | 2 | 5 | 4.941 | 3.61 | 0.09 | 0.35 | 0.27 |
| **11** | 2.904 | 2.092 | 1 | 2 | 5 | 4.941 | 2.20 | 1.66 | 0.29 | 0.26 |
| **Recomm.** | 4.5 | 5 | 3 | 7 | 8 | 7 | ≥5 | ≥5 | ≥0.3 | ≥0.3 |
